# AURKB inhibition induces rhabdomyosarcoma apoptosis and ferroptosis through NPM1/SP1/ACSL5 axis

**DOI:** 10.1172/jci.insight.182429

**Published:** 2025-02-10

**Authors:** Huimou Chen, Mengzhen Li, Yu Zhang, Mengjia Song, Yi Que, Juan Wang, Feifei Sun, Jia Zhu, Junting Huang, Juan Liu, Jiaqian Xu, Suying Lu, Yizhuo Zhang

**Affiliations:** 1 Department of Pediatric Oncology and; 2 State Key Laboratory of Oncology in South China, Guangdong Provincial Clinical Research Center for Cancer, Collaborative Innovation Center for Cancer Medicine, Sun Yat-sen University Cancer Center, Guangzhou, China.; 3 Department of Oncology, Sun Yat-sen Memorial Hospital of Sun Yat-sen University, Guangzhou, China.

**Keywords:** Oncology, Oncogenes

## Abstract

Rhabdomyosarcoma (RMS) is one of the most common solid tumors in children and adolescents. Patients with relapsed/refractory RMS have limited treatment options, highlighting the urgency for the identification of novel therapeutic targets for RMS. In the present study, aurora kinase B (AURKB) was found to be highly expressed in RMS and associated with unfavorable prognosis of patients. Functional experiments indicated that inhibition of AURKB significantly reduced RMS cell proliferation, induced apoptosis and ferroptosis, and suppressed RMS growth in vivo. The highly expressed AURKB in RMS contributes to the apoptosis and ferroptosis resistance of tumor cells through the nucleophosmin 1 (NPM1)/Sp1 transcription factor (SP1)/acyl-CoA synthetase long-chain family member 5 (ACSL5) axis. Furthermore, inhibition of AURKB exerted an anti-RMS effect together with vincristine both in vitro and in vivo, with tolerable toxicity. The above findings provide insights we believe are new into the tumorigenesis of RMS, especially with regard to apoptosis or ferroptosis resistance, indicating that AURKB may be a potential target for clinical intervention in patients with RMS.

## Abstract 2

The above findings provide new insights into the tumorigenesis especially the apoptosis or ferroptosis resistance of RMS, and indicated that AURKB may be a potential target for clinical intervention in RMS patients.

## Graphical abstract

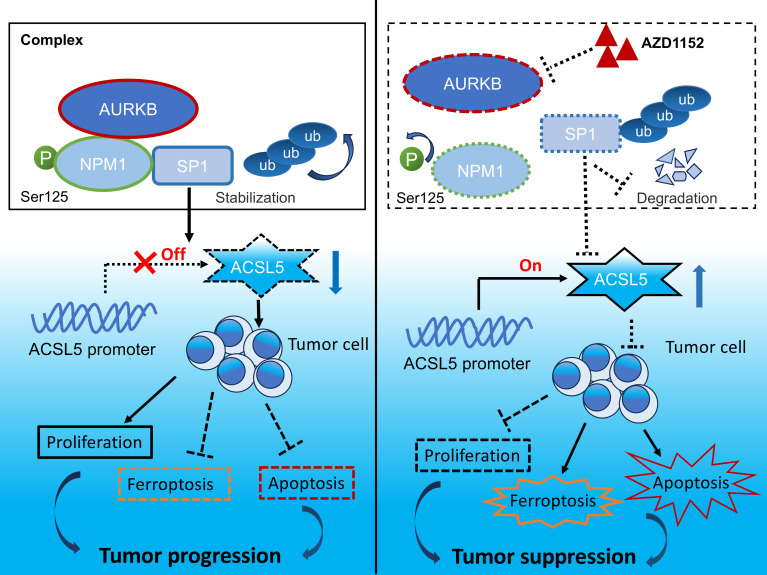

## Introduction

Rhabdomyosarcoma (RMS) is the most common pediatric soft tissue sarcoma, which arises from muscle precursor cells and accounts for about 50% of all pediatric soft tissue sarcoma (1). Over the past 30 years, the survival rate of patients with nonmetastatic diseases has been improved substantially. Approximately 75% of patients with localized diseases have been cured with conventional multimodal therapy, including chemotherapy coordinated with surgery and radiotherapy, but those with recurrent or metastatic diseases have the overall survival rates of only 17% and 30%, respectively (2, 3). Intrinsic or acquired resistance to apoptosis is one of the major factors leading to therapy failure (4), suggesting an urgent need to develop effective approaches to overcome apoptosis resistance or to trigger nonapoptotic forms of programmed cell death in RMS.

Ferroptosis is one of the nonapoptotic forms of programmed cell death, which is caused by massive lipid peroxidation-mediated membrane damage (5). Increasing research has revealed that dysregulation of ferroptosis is implicated in human cancers (5–7). Inhibition of antiferroptosis capacity can prevent acquired resistance of cancer cells to several cancer therapies, including chemotherapy, targeted therapy, and radiotherapy (8). Therefore, inducing ferroptosis may provide a novel potential strategy for cancer therapy.

Aurora kinase B (AURKB) is a serine-threonine kinase belonging to the highly conserved aurora family of mitotic kinases (9), which is involved in a variety of biological processes, including spindle checkpoint, chromosome segregation, and cytokinesis. An aberrant expression of AURKB in tumors causes chromosomal instability and aneuploidy, leading to tumorigenesis, tumor migration, and invasion (10–17). However, the biological role of AURKB and its regulatory mechanism in RMS remain unclear.

In the present study, the tumor-promoting role of AURKB in RMS was identified by genetic knockdown and using a highly selective AURKB inhibitor, AZD1152, and we found the regulatory effect of AURKB on ferroptosis in RMS. The exact molecular regulatory mechanism of AURKB in RMS was also demonstrated. Furthermore, AURKB inhibition could enhance the chemotherapeutic efficacy of vincristine (VCR), and this finding sheds light on the utilization of AURKB as a potential novel therapeutic target for RMS.

## Results

### Bioinformatics analysis of RMS tissues.

**Figure 1:**
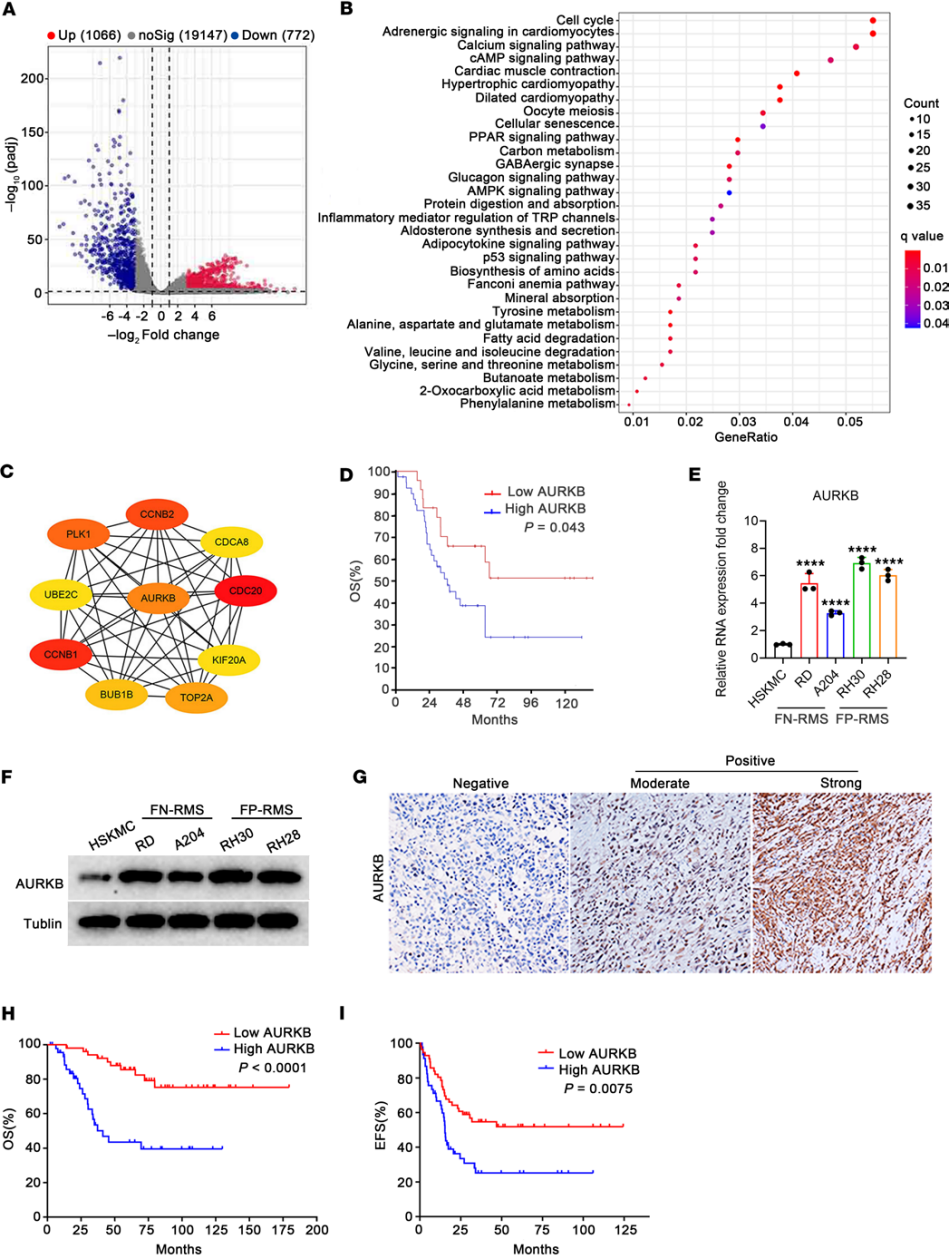
AURKB was upregulated in RMS and associated with poor prognosis of patients. (**A**) Volcano diagrams of DEGs from the GSE108022 dataset. (**B**) KEGG pathway analysis for DEGs in the GSE108022 dataset. (**C**) The top 10 hub genes from all of the DEGs were identified based on PPI analysis. (**D**) Kaplan-Meier survival analysis of AURKB expression based on genomics analysis with the R2 platform (https://hgserver1.amc.nl/cgi-bin/r2/main.cgi?open_page=login). (**E**) mRNA expression levels of AURKB in HSKMCs and 4 RMS cell lines. (**F**) Protein expression levels of AURKB in HSKMCs and 4 RMS cell lines. (**G**) Representative images of AURKB protein expression in patients with RMS detected by IHC (original magnification, ×200). (**H** and **I**) Overall survival (OS) curve and event-free survival (EFS) curve of patients with RMS expressing high levels or low levels of AURKB at Sun Yat-sen University Cancer Center. FN, fusion negative; FP, fusion positive. ****P < 0.0001, log-rank test (**D**, **H**, and **I**) and 1-way ANOVA (**E**).

Using the Gene Expression Omnibus (GEO) GSE108022 dataset, 1,838 differentially expressed genes (DEGs) (1,066 upregulated and 772 downregulated) in RMS samples were identified. Volcano plots of all DEGs are shown in Figure 1A. Results of Kyoto Encyclopedia of Genes and Genomes (KEGG) pathway analysis demonstrated that DEGs were particularly enriched in the “cell cycle” signaling pathway (Figure 1B), which had the highest gene ratio score. It is worth noting here that the next 5 top-scoring hits of the KEGG pathway analysis involved adrenergic signaling in cardiomyocytes, the calcium signaling pathway, the cAMP signaling pathway, hypertrophic cardiomyopathy, and dilated cardiomyopathy, which are all related to cardiac and skeletal muscle signaling, consistent with the potential pathogenesis origin of RMS, which was related to the process of muscle development (1, 3). The top 10 hub genes in tumors were CDCA8, PLK1, KIF20A, CDC20, AURKB, CCNB2, BUBIB, TOP2A, CCNB1, and UBE2C (Figure 1C), among which AURKB was found to have the highest expression level and to be closely associated with an unfavorable prognosis of patients with RMS (Figure 1D). Therefore, AURKB was selected for further study.

### AURKB was upregulated in RMS and associated with a poor prognosis of patients.

The results of quantitative real-time PCR (qRT-PCR) and Western blotting (WB) confirmed a significantly upregulated level of AURKB in RMS cell lines (Figure 1, E and F). IHC staining (Figure 1G) on RMS tissue sections revealed a high AURKB expression rate of 46.9% in 98 RMS cases, which was significantly associated with the advanced IRS stage (*P* = 0.022, Table 1). Among patients with RMS with low or high expression of AURKB, the 5-year overall survival rate was 85.6% versus 43.5% (*P* < 0.0001, Figure 1H) and the 5-year event-free survival rate was 47.9% versus 25.3% (*P* = 0.0075, Figure 1I).

### Anticancer activity of AURKB inhibition in RMS cell lines.

**Figure 2:**
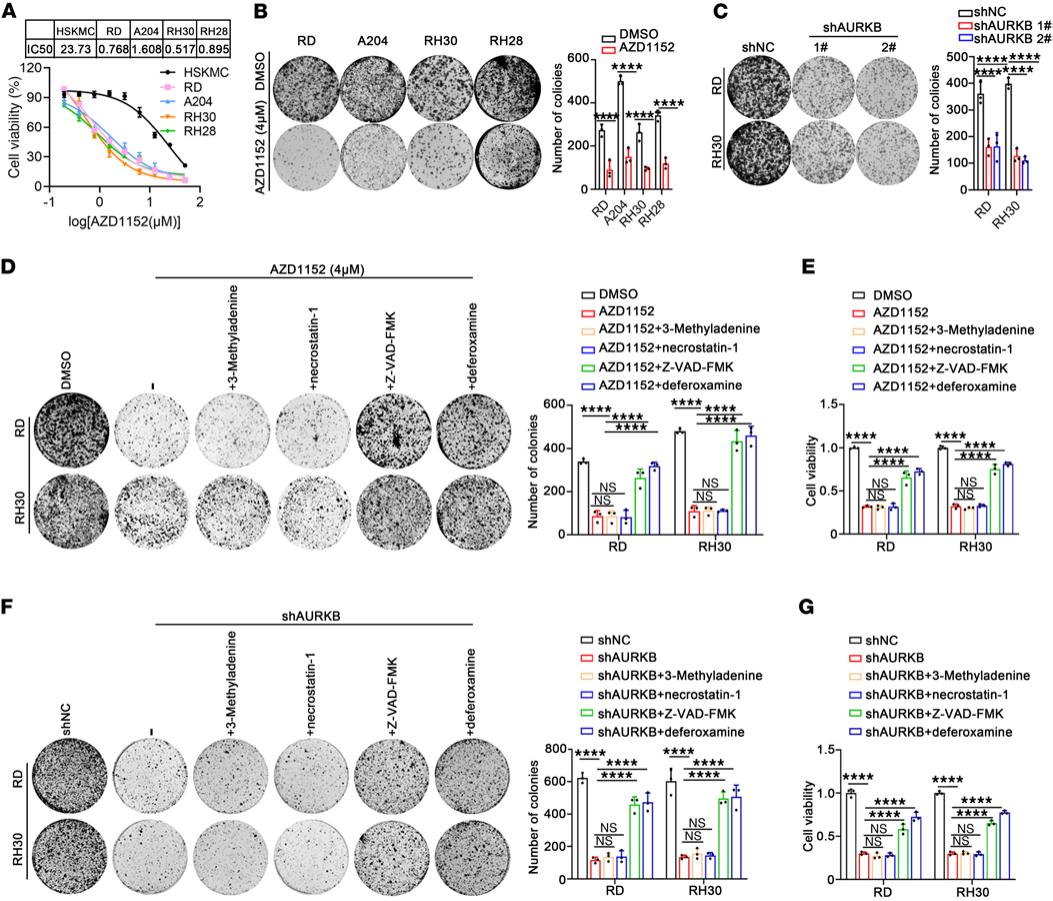
AURKB inhibition exerted an anticancer effect in RMS cell lines. (**A**) Cell viability analysis by CCK-8 assay in HSKMCs and 4 RMS cell lines after treatment with different concentrations of AZD1152 (with the initially maximum concentration [50 μM] diluted at a gradient at a ratio of 2:1). (**B**) Colony formation assay of 4 RMS cell lines after treatment with 4 μM AZD1152 for 48 hours. (**C**) Colony formation assay of RD and RH30 cells after AURKB knockdown with shRNA. (**D**) Colony formation assay of RD and RH30 cells treated with AZD1152 in the absence or presence of indicated cell death inhibitors (50 μM 3-methyladenine, 10 μM necrostatin-1, 10 μM Z-VAD-FMK, and 10 μM deferoxamine) for 48 hours. (**E**) Viability of RD and RH30 cells treated with AZD1152 was evaluated in the absence or presence of indicated cell death inhibitors for 48 hours. (**F**) Colony formation assay in RMS cells with AURKB genetic knockdown in the absence or presence of indicated cell death inhibitors for 48 hours. (**G**) Cell viability of RD and RH30 cells after AURKB knockdown was evaluated in the absence or presence of indicated cell death inhibitors for 48 hours. ****P < 0.0001, 2-way ANOVA.

Next, the role of AURKB in RMS was preliminarily explored using the AURKB inhibitor AZD1152 or genetic knockdown. AZD1152 killed human RMS cell lines, including RD, RH30, A204, and RH28, in vitro in a dose-dependent manner. In contrast, normal human skeletal muscle cells (HSKMCs) were resistant to AZD1152 (Figure 2A). The reproductive integrity of RMS cells after AZD1152 (4 μM) treatment or AURKB genetic knockdown was significantly reduced (Figure 2, B and C). To sum up, AURKB inhibition has an anticancer activity in human RMS cells.

To investigate the mechanism in suppressing RMS proliferation after AURKB inhibition, the cell death mode was analyzed. The apoptosis inhibitor Z-VAD-FMK, cell necrosis inhibitor necrostatin-1, cell autophagy inhibitor 3-methyladenine, and the ferroptosis inhibitor deferoxamine were used. The results showed that there was no significant change in the colony-formation capacity and cell viability after treatment with AZD1152 plus necrostatin-1 or 3-methyladenine, indicating that AZD1152 cannot induce cell necrosis or cell autophagy in RMS. However, the effect of AZD1152 was partially inhibited by Z-VAD-FMK or deferoxamine (Figure 2, D and E). Consistently, Z-VAD-FMK or deferoxamine partially eliminated the inhibitory effect of AURKB knockdown (Figure 2, F and G). Taken together, the effect of AURKB inhibition may be partly achieved by inducing apoptosis and ferroptosis of RMS cells.

### AURKB inhibition induced apoptosis and ferroptosis in RMS cell lines.

**Figure 3:**
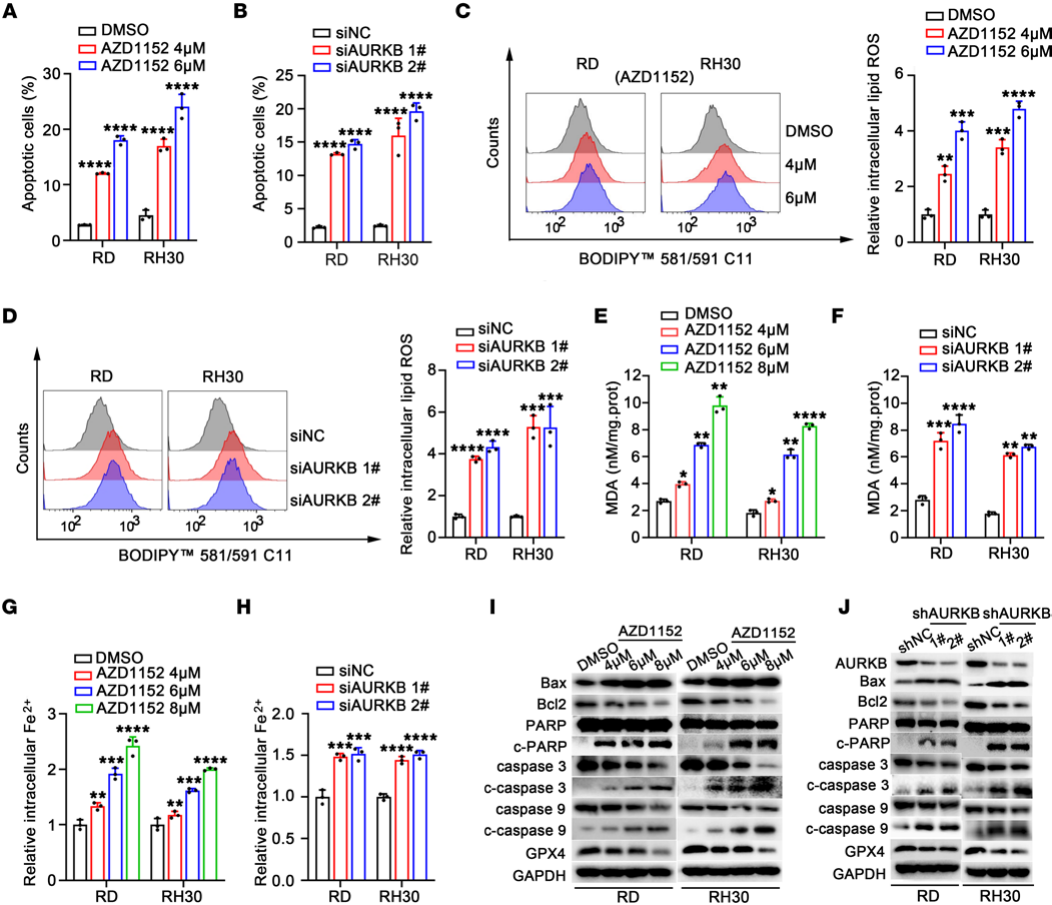
AURKB inhibition induced apoptosis and ferroptosis in RMS cell lines. (**A** and **B**) Columnar statistical charts revealed changes in the apoptosis proportion of RMS cell lines after AURKB inhibition with different concentrations of AZD1152 or siRNA after transfection for 72 hours. (**C** and **D**) The intracellular lipid ROS levels in RMS cells after treatment with different concentrations of AZD1152 or AURKB genetic knockdown with siRNA transfection for 72 hours. (**E** and **F**) The intracellular MDA levels in RMS cells after treatment with AZD1152 at different concentrations or AURKB genetic knockdown with siRNA transfection for 72 hours. (**G** and **H**) Columnar statistical charts revealed changes of the relative intracellular Fe2+ levels in RMS cells after treatment with AZD1152 at different concentrations or AURKB RNA silencing with siRNA transfection for 72 hours. (**I** and **J**) WB analysis showed changes of indicated apoptosis-related gene markers and the ferroptosis-related marker GPX4 after AURKB inhibition with AZD1152 treatment at different concentrations or genetic knockdown with shRNA in RMS cells. *P < 0.05, **P < 0.01, ***P < 0.001, ****P < 0.0001, *t* test.

The exact role of AURKB inhibition in inducing apoptosis and ferroptosis of RMS cells was then investigated. As expected, AZD1152 significantly induced RMS cell apoptosis in a dose-dependent manner (Figure 3A). Consistently, similar results were observed after AURKB knockdown (Figure 3B). Additionally, the accumulation of intracellular lipid ROS and malondialdehyde (MDA) and the cellular Fe2^+^ content were increased synchronously (Figure 3, C–H). The WB results (Figure 3, I and J) indicated that antiapoptotic protein Bcl2 was decreased and proapoptotic protein Bax was dramatically increased following AURKB inhibition. Suppressing AURKB could also cause the cleavage of PARP, caspase-9, and caspase-3. Additionally, AZD1152 treatment and genetic knockdown of AURKB decreased the expression of GPX4, a protein marker for ferroptosis.

### AURKB exerted a tumor-promoting effect in vivo.

**Figure 4:**
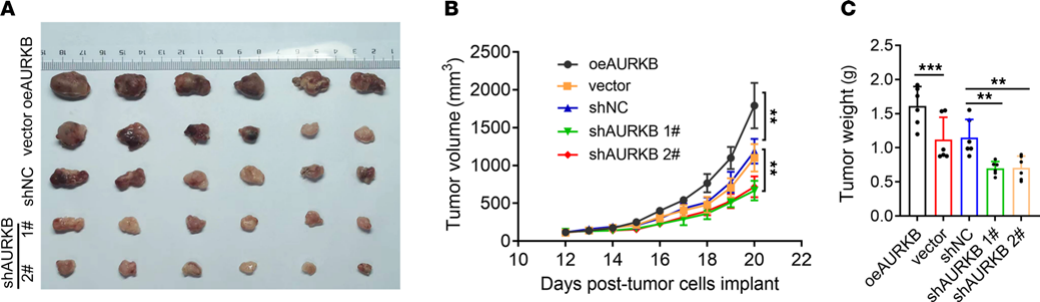
AURKB promoted tumorigenesis in RMS xenograft mouse models. (**A**) Images of harvested tumors of RH30 tumor-bearing mice. (**B**) Tumor volumes of RH30 xenograft models with genetic overexpression or knockdown of AURKB. (**C**) Tumor weight of independent groups was recorded at the endpoint of the experiment. Data are shown as mean ± SD of 6 mice in each group. **P < 0.01, ***P < 0.001, 1-way ANOVA (**B**) and *t* test (**C**).

The xenograft model in mice further verified the tumor-promoting effect of AURKB in vivo. The tumor growth rate in the oeAURKB group was significantly higher than that in the vector group and shAURKB group, with the shAURKB group having the lowest rate (Figure 4, A and B). Accordingly, tumor weight in the oeAURKB group was significantly larger than that in the vector group and shAURKB group, with the shAURKB group having the smallest weight (Figure 4C). Consistently, the AURKB inhibitor AZD1152 suppressed RMS growth in vivo (Supplemental Figure 1; supplemental material available online with this article; https://doi.org/10.1172/jci.insight.182429DS1).

### AURKB promoted apoptosis and ferroptosis resistance of RMS via phosphorylating NPM1 at Ser125.

**Figure 5:**
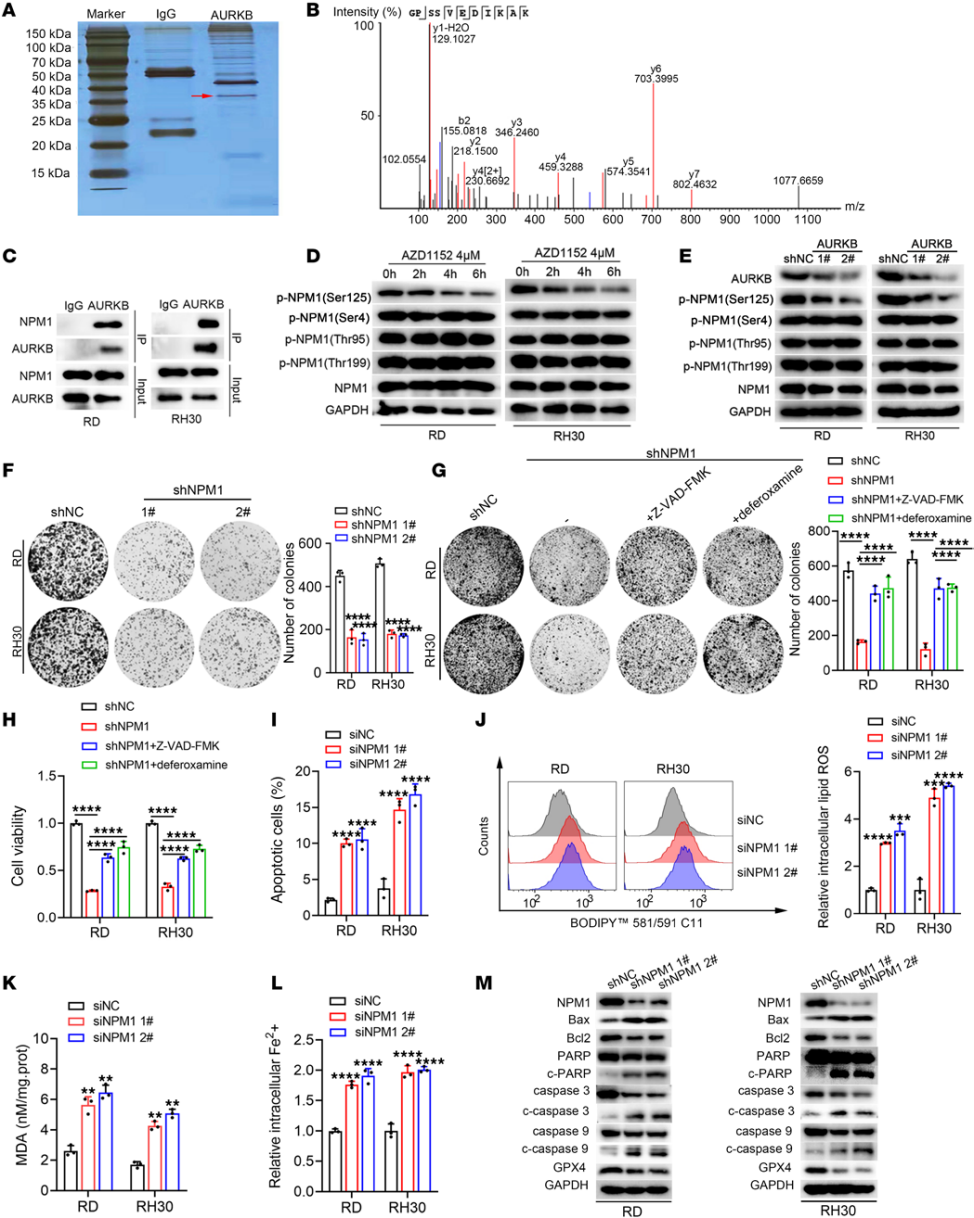
AURKB promoted apoptosis and ferroptosis resistance of RMS via phosphorylating Ser125 of NPM1. (**A**) Silver staining after coimmunoprecipitation with AURKB antibody. The arrow denotes the distinct bands between 35 kDa and 40 kDa. (**B**) Results of protein mass spectrometry indicated the physical interaction of AURKB and NPM1. (**C**) Results of immunoprecipitation to verify the interaction of AURKB and NPM1. (**D**) Time-course analysis of WB identified expression changes of p-NPM1 at Ser125, Ser4, Thr95, Thr199, and the total NPM1 in RMS lines treated with AZD1152. (**E**) WB analysis identified expression changes of the p-NPM1 at Ser125, Ser4, Thr95, Thr199, and the total NPM1 in RMS lines after genetic knockdown of NPM1. (**F**) Colony formation assay after genetic knockdown of NPM1 with shRNA in RMS cell lines. (**G**) Colony formation assay evaluating proliferation ability after shRNA knockdown of NPM1 in RMS cells treated with or without apoptosis inhibitor (10 μM Z-VAD-FMK) or ferroptosis inhibitor (10 μM deferoxamine) for 48 hours. (**H**) Cell viability evaluated in RD and RH30 cell lines after knocking down NPM1 with shRNA in RMS cells treated with or without Z-VAD-FMK or deferoxamine treatment for 48 hours. (**I**) The apoptosis rate changes after NPM1 genetic knockdown with siRNA transfected for 72 hours in RMS cells. (**J**) Changes of the intracellular lipid ROS levels after NPM1 suppression with siRNA transfected for 72 hours. (**K**) Changes of the intracellular MDA levels after NPM1 suppression with siRNA transfected for 72 hours. (**L**) Changes of relative intracellular Fe2+ levels in RMS cells after NPM1 was silenced by siRNA transfection for 72 hours. (**M**) WB analysis revealed the changes of indicated apoptosis-related gene markers and the ferroptosis-related marker GPX4 after NPM1 inhibition with shRNA in RMS cells. **P < 0.01, ***P < 0.001, ****P < 0.0001, 2-way ANOVA (**F**–**H**) and *t* test (**I**–**L**).

It has been previously reported that AURKB mainly modulates its target genes posttranscriptionally (18), so mass spectrometry was then conducted in RMS cells. In detail, the silver staining results revealed that the anti-AURKB antibody pulled down distinct bands between 35 kDa and 40 kDa, among which nucleophosmin 1 (NPM1) was identified to have the highest intensity (Figure 5, A and B). Considering the high intensity of NPM1 and its potential role in regulating ferroptosis (19, 20), it was speculated that AURKB might modulate ferroptosis in RMS through NPM1. As expected, AURKB interacted with NPM1 with an interaction coefficient of 0.648 (http://www.hitpredict.org/), which was further confirmed by immunoprecipitation (Figure 5C). Interestingly, AZD1152 treatment caused a notable decrease in the phosphorylation level of NPM1 at Ser125 (p-NPM1 Ser125), while the phosphorylation levels of other common phosphorylation sites, Ser4, Thr95, and Thr199, and the total NPM1 had no obvious changes (Figure 5D). Similar results were found after genetic knockdown of AURKB (Figure 5E).

NPM1 was found to be highly expressed in RMS and associated with a worse prognosis based on the bioinformatic analysis (Supplemental Figure 2). Knockdown of NPM1 significantly weakened proliferation (Figure 5, F–H) and induced apoptosis and ferroptosis of RMS cells (Figure 5, I–M). Moreover, it was verified that the phosphorylated status of NPM1 at Ser125 was important for RMS cell proliferation (Supplemental Figure 3). Further functional assays demonstrated that the reintroduction of NPM1 partly reversed the growth arrest, apoptosis, and ferroptosis of tumor cells induced by AURKB knockdown (Supplemental Figure 4). These findings provided strong evidence that AURKB regulates proliferation, apoptosis, and ferroptosis resistance of RMS cells via phosphorylating NPM1 at Ser125.

### AURKB inhibited ACSL5 expression through NPM1.

**Figure 6:**
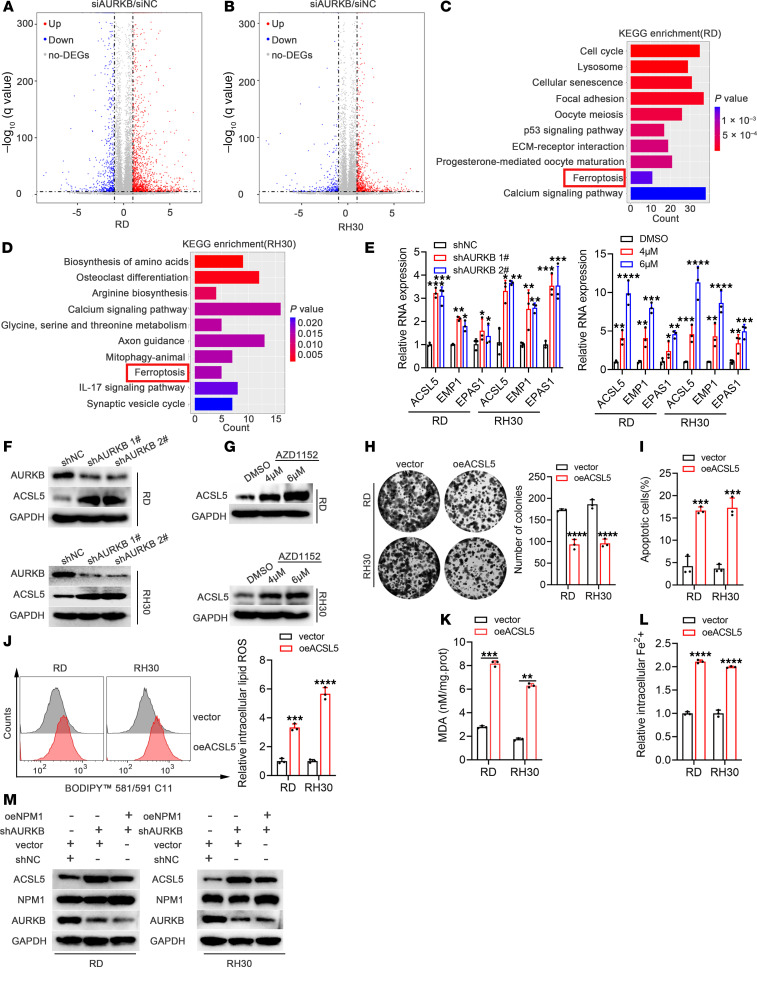
AURKB inhibited ACSL5 expression through NPM1. (**A** and **B**) Volcano diagrams of DEGs from RD and RH30 cells with AURKB genetic knockdown by siRNA transfection for 72 hours. (**C** and **D**) KEGG pathway analysis enriched by DEGs from RD and RH30 cells after AURKB suppression by siRNA transfection for 72 hours. (**E**) mRNA expression levels of DEGs in the ferroptosis pathway validated in RMS cells after AURKB genetic knockdown by shRNA or treated with AZD1152 at different concentrations for 48 hours. (**F** and **G**) Results of WB analysis to confirm the protein expression changes of ACSL5 in the ferroptosis pathway after AURKB inhibition by shRNA or treated with AZD1152 at different concentrations for 48 hours. (**H**) Colony formation assay after overexpression of ACSL5 in RMS cells. (**I**) Changes of apoptosis rates after overexpression of ACSL5 in RMS cells. (**J**) Changes of the intracellular lipid ROS levels after ACSL5 overexpression in RMS cells. (**K**) Changes of the MDA levels after ACSL5 overexpression in RMS cells. (**L**) Changes of the relative intracellular Fe2+ levels in RMS cells after ACSL5 overexpression. (**M**) WB analysis of the ACSL5 protein expression in siAURKB RMS cells treated with or without overexpressing NPM1. *P < 0.05, **P < 0.01, ***P < 0.001, ****P < 0.0001, *t* test (**E** and **I**–**L**) and 2-way ANOVA (**H**).

To further understand the molecular mechanism underlying AURKB regulation of proliferation, apoptosis, and ferroptosis of RMS cells, RNA-Seq analysis was performed on RMS cells transfected with siNC or siAURKB. Volcano plots of DEGs are shown in Figure 6, A and B. KEGG pathway analysis demonstrated that DEGs were enriched in the ferroptosis pathway (Figure 6, C and D). qRT-PCR verified that AURKB inhibition upregulated the expression of genes in the ferroptosis pathway, including acyl-CoA synthetase long-chain family member 5 (ACSL5) (21), EMP1 (22), and EPAS1 (23), with ACSL5 being the most upregulated gene (Figure 6E). WB further confirmed that inhibition of AURKB upregulated the expression of ACSL5 (Figure 6, F and G).

Moreover, overexpression of ACSL5 caused significant growth arrest (Figure 6H), apoptosis (Figure 6I), and ferroptosis of RMS cells (Figure 6, J–L). Therefore, it was hypothesized that AURKB inhibition of ACSL5 expression might be regulated through NPM1. Consistently, the reintroduction of NPM1 partially reversed the upregulation of ACSL5 induced by AURKB knockdown (Figure 6M).

### AURKB inhibited ACSL5 expression through the NPM1/SP1 axis.

**Figure 7:**
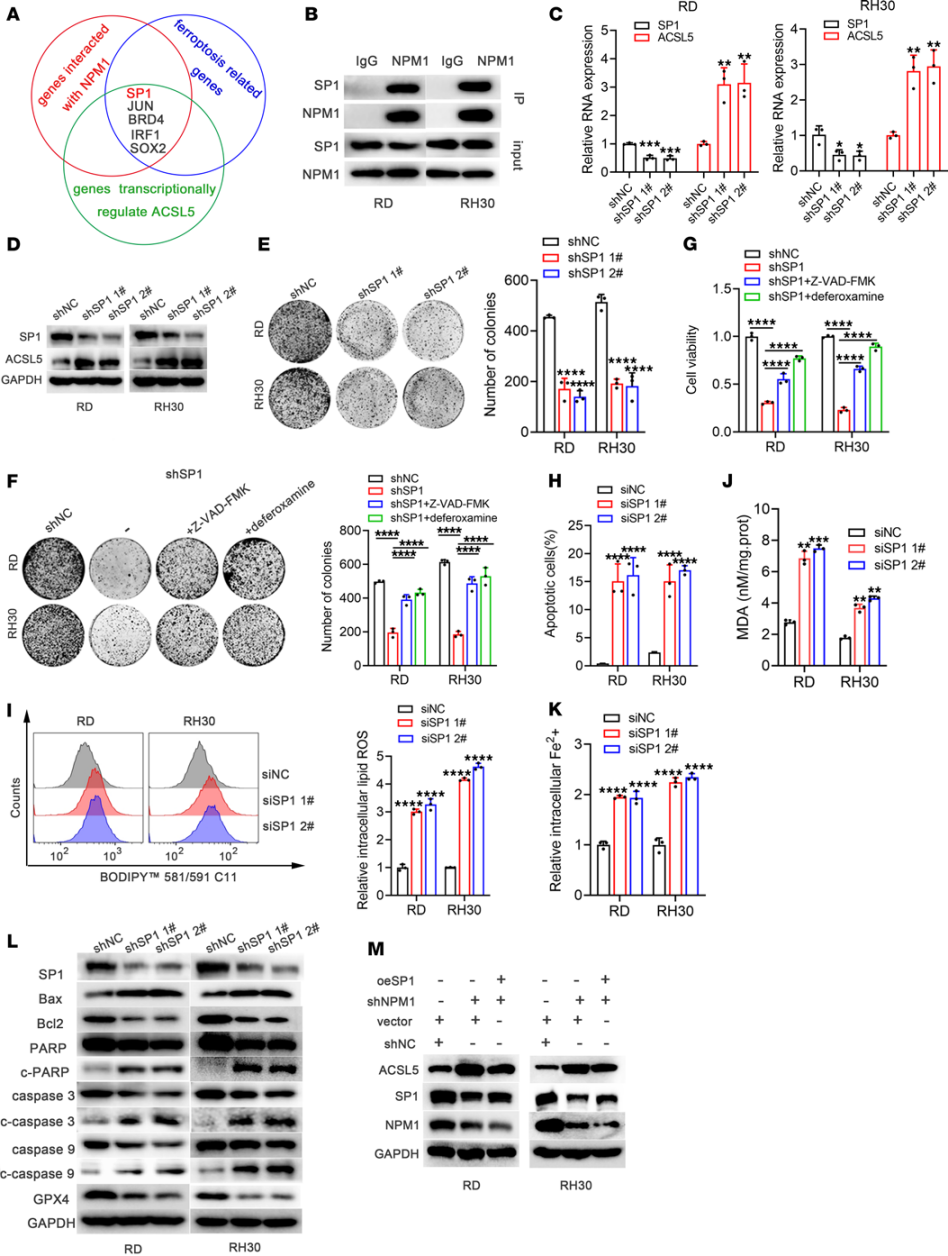
NPM1 inhibited ACSL5 expression by binding SP1. (**A**) The 5 overlapped genes (including SP1, JUN, BRD4, IRF1, and SOX2), which can interact with NPM1 and were related to ferroptosis, can transcriptionally regulate ACSL5 as well. (**B**) Immunoprecipitation in RD and RH30 cells to verify the interaction of NPM1 and SP1. (**C**) mRNA expression levels of ACSL5 validated by qRT-PCR in RD and RH30 cells after genetic knockdown of SP1 with shRNA. (**D**) WB to identify protein expression levels of ACSL5 in RD and RH30 cells after genetic knockdown of SP1 with shRNA. (**E**) Colony formation assay in RMS cells after SP1 knockdown by shRNA. (**F**) Colony formation assay in RMS cells after knocking down SP1 with shRNA in RMS cells treated with or without apoptosis inhibitor (10 μM Z-VAD-FMK) or ferroptosis inhibitor (10 μM deferoxamine) for 48 hours. (**G**) Cell viability in RMS cells with SP1 genetic knockdown with shRNA in the absence or presence of Z-VAD-FMK or deferoxamine for 48 hours. (**H**) Changes of apoptosis rates after knocking down SP1 with siRNA transfection for 72 hours in RMS cells. (**I**–**K**) Changes of the intracellular lipid ROS levels, MDA, and the relative intracellular Fe2+ levels in RMS cells after SP1 knockdown with siRNA transfection for 72 hours. (**L**) WB analysis revealed changes in indicated apoptosis-related gene markers and the ferroptosis-related marker GPX4 after SP1 inhibition with shRNA in RMS cells. (**M**) WB analysis of the ACSL5 protein expression in shNPM1 RMS cells treated with or without overexpression of SP1. *P < 0.05, **P < 0.01, ***P < 0.001, ****P < 0.0001, *t* test (**C** and **H**–**K**) and 2-way ANOVA (**E**–**G**).

It can be concluded that NPM1 promoted apoptosis and ferroptosis resistance in RMS by inhibiting the expression of ACSL5. However, the detailed molecular mechanism remains unclear. To solve this problem, the bioinformatics analysis was conducted. The overlapped genes interacting with NPM1 (HitPredict database; https://www.hitpredict.org/), genes related to ferroptosis (GeneCards database; https://www.genecards.org/), and genes that can transcriptionally regulate ACSL5 (animal TFDB database; https://guolab.wchscu.cn/AnimalTFDB#!/tfbs_predict) contained 5 known oncogenes in RMS, including Sp1 transcription factor (SP1), JUN, BRD4, IRF1, and SOX2 (Figure 7A). The SP1 score was the highest in Animal TFDB database (Supplemental Table 2). Therefore, it was inferred that NPM1 may inhibit ACSL5 transcription by binding SP1 protein in RMS. The immunoprecipitation results confirmed that NPM1 interacted with SP1 (Figure 7B).

It’s worth noting that, knockdown of AURKB or NPM1 significantly decreased SP1 at the protein rather than the mRNA level (Supplemental Figure 5, A–D), which indicated the stability of the SP1 protein is affected by AURKB/NPM1 rather than the posttranscriptional-regulation model of SP1. To identify the exact posttranslational regulation method of SP1, we then investigated the effect of lysosomal inhibitor (NH4Cl, 3-MA), apoptosis inhibitor Z-VAD-FMK, or proteasome inhibitor MG132 on the overall level of SP1. It was found that only MG132 increased the level of SP1, indicating the manner of degradation of SP1 was ubiquitin-proteasome system dependent in RD cells (Supplemental Figure 5E). Knockdown of AURKB or NPM1 could significantly increase the protein ubiquitination of SP1 (Supplemental Figure 5, F–K).

Furthermore, knockdown of SP1 significantly upregulated ACSL5 at both the mRNA and protein levels (Figure 7, C and D), suppressed cell proliferation (Figure 7E), and induced apoptosis and ferroptosis (Figure 7, F–L).

SP1 is a well-known transcription factor, which can transcriptionally activate or silence the downstream target molecules. We speculated that its modulation of ACSL5 was dependent on its transcriptional regulatory function. As expected, SP1 was demonstrated to enrich at the promoter region of ACSL5 and transcriptionally silenced the expression of ACSL5 (Supplemental Figure 6). NPM1 knockdown–induced upregulation of ACSL5 could be partially reversed by overexpression of SP1 (Figure 7M).

**Figure 8:**
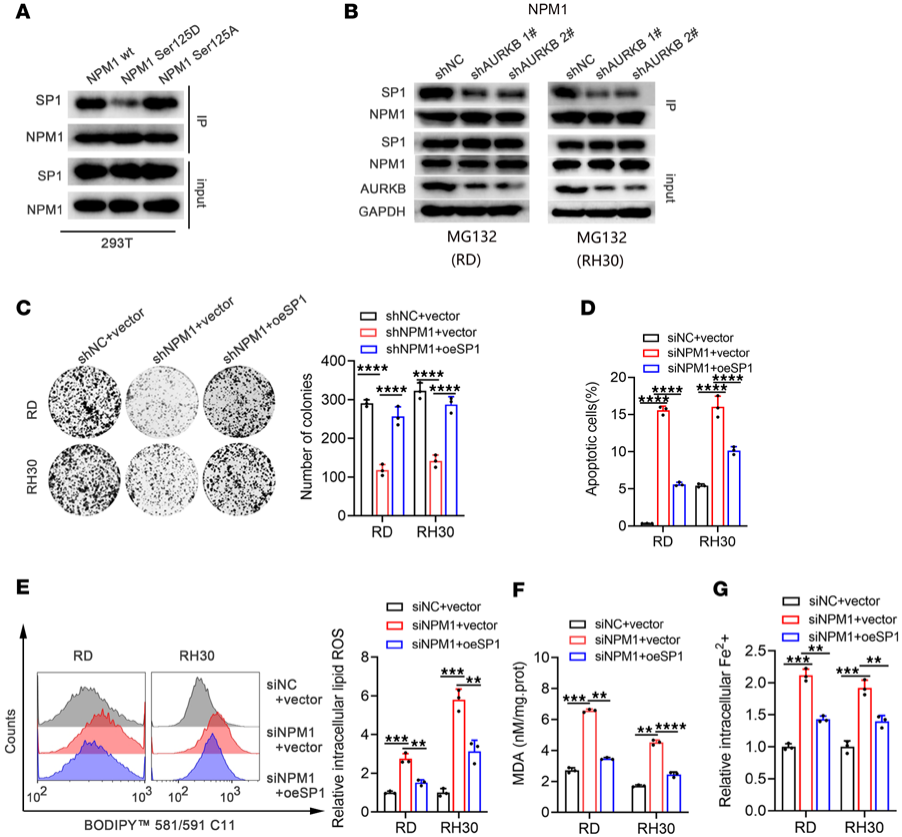
The phosphorylated NPM1 at Ser125 by AURKB was important for the binding of NPM1 to SP1. (**A**) Coimmunoprecipitation of SP1 and NPM1 (WT, Ser125D, or Ser125A) from lysates of 293T cells. (**B**) Coimmunoprecipitation analysis to detect the influence of the interaction of NPM1 and SP1 after AURKB knockdown. (**C**–**G**) Colony formation assay, apoptosis assay, intracellular lipid ROS level, and MDA and intracellular Fe2+ level detection in siNPM1/shNPM1 RMS cells with or without reintroduction of SP1. **P < 0.01, ***P < 0.001, ****P < 0.0001, *t* test (**D**–**G**) and 2-way ANOVA (**C**).

In this study, it was found that NPM1 interacted with AURKB and SP1 (Supplemental Figure 7), which motived us to wonder about the role of NPM1 phosphorylation in affecting the binding of NPM1 to SP1. The results showed that the phosphorylated status of NPM1 at Ser125 by AURKB was important for the binding of NPM1 to SP1 (Figure 8A). Consistently, knockdown of AURKB impaired the interaction of NPM1 and SP1 (Figure 8B). The reintroduction of SP1 could partially reverse the apoptosis and ferroptosis of tumor cells induced by NPM1 knockdown (Figure 8, C–G). Therefore, the above results revealed that AURKB inhibited ACSL5 expression through the NPM1/SP1 axis.

### AURKB inhibition enhanced anti-RMS activity of VCR.

**Figure 9:**
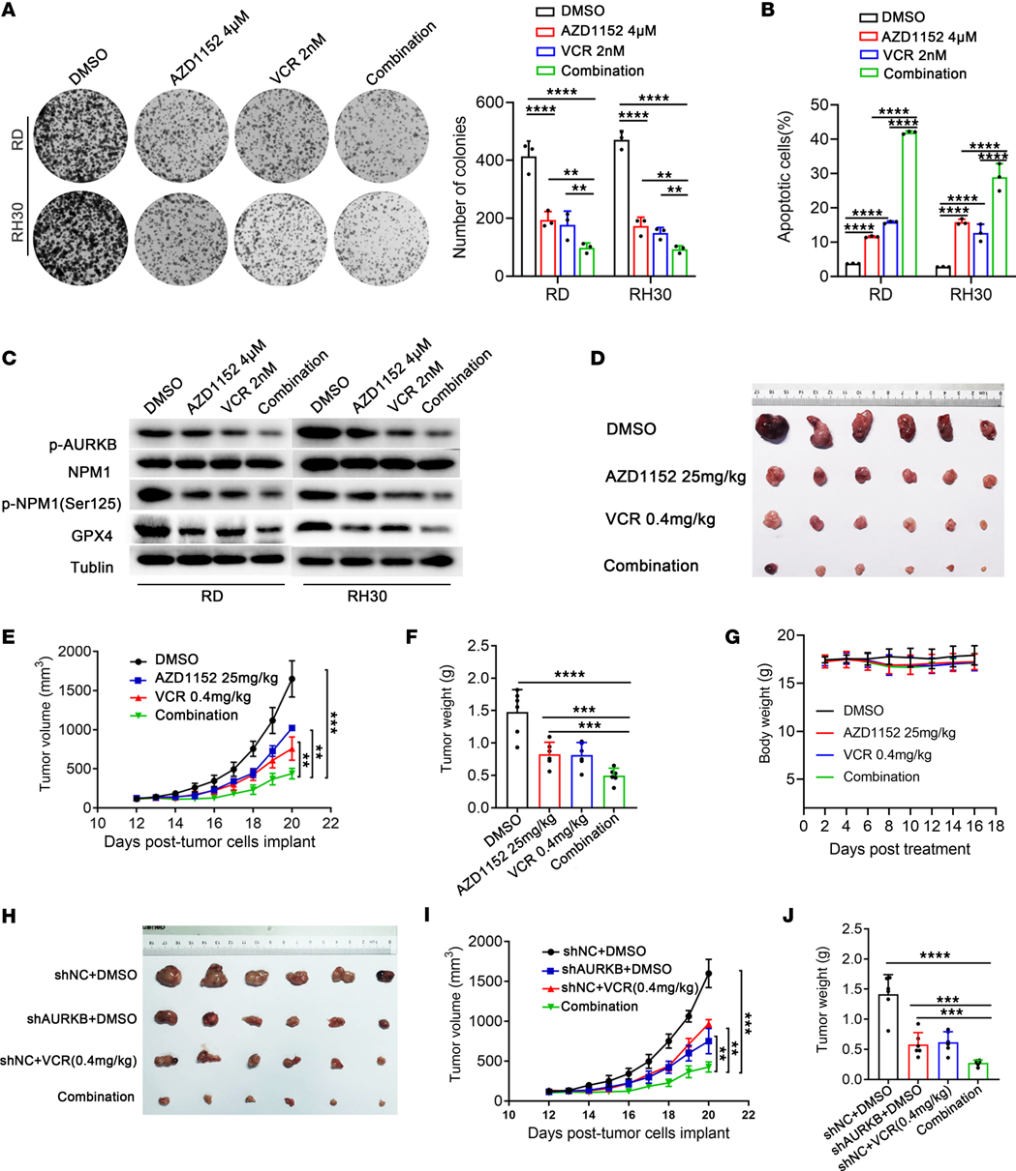
AURKB inhibition enhanced the anti-RMS activity of VCR. 4 μM AZD1152 and 2 nM VCR treated for 48 hours were used for the in vitro experiment, and 25 mg/kg AZD1152 for day 1, day 3, day 5, and day 7 with and without 0.4 mg/kg VCR for day 1 were used for the in vivo experiment. (**A**) Colony formation assay of RD and RH30 cells after different treatments. (**B**) Apoptosis changes in RD and RH30 cells under different treatments. (**C**) WB identifying the synergistic changes of p-AURKB, p-NPM1(Ser 125), and GPX4 in different groups after treatment for 48 hours. (**D**) Images of harvested tumors of RH30 tumor-bearing mice in indicated groups. (**E**) Tumor volumes of RH30 xenograft models in different groups. (**F**) Weight of tumors treated with AZD1152 and VCR alone or in combination was recorded at the endpoint of the experiment. (**G**) Body weight of mice from 4 individual groups. (**H**) Images of harvested tumors of RH30 tumor-bearing mice after treatment with shAURKB and VCR alone or in combination. (**I**) Tumor volumes of RH30 xenograft models after treatment with shAURKB and VCR alone or in combination. (**J**) Weight of tumors after treatment with shAURKB and VCR alone or in combination. ^**^P < 0.01, ^***^P < 0.001, ^****^P < 0.0001, 2-way ANOVA (**A**), *t* test (**B**, **F**, and **J**), and 1-way ANOVA (**E** and **I**).

As a highly selective inhibitor of AURKB, AZD1152 can overcome resistance to EGFR inhibitors in lung cancer via enhancing apoptosis, modulate paclitaxel response in non–small cell lung cancer, and enhance chemotherapeutic efficacy in pancreatic and colon cancers (15, 16, 24). Therefore, we wondered whether AZD1152 can synergistically enhance the therapeutic efficacy of the first-line chemotherapy agents for RMS. Combination indexes, calculated by CompuSyn software, for AZD1152 combined with VCR, doxorubicin, and actinomycin were less than 1, with the combination with VCR having the lowest index (Supplemental Tables 3 and 4). Therefore, VCR was selected for further investigation. AZD1152 combined with VCR could synergistically induce growth arrest and cell apoptosis in vitro (Figure 9, A and B). In terms of the mechanism, AZD1152 could work synergistically with VCR to inhibit phosphorylated AURKB (p-AURKB) and p-NPM1(Ser125), suppressing the antiferroptosis marker GPX4 (Figure 9C). Consistently, the synergistically anti-RMS effect of AURKB inhibition (AZD1152, Figure 9, D–G, or genetic knockdown with shRNA, Figure 9, H–J) combined with VCR was also verified in vivo. Moreover, IHC staining also showed that AURKB inhibition could repress GPX4 expression and RMS growth together with VCR (Supplemental Figure 8). Taken together, AURKB inhibition in combination with VCR can synergistically impede RMS growth.

**Figure 10:**
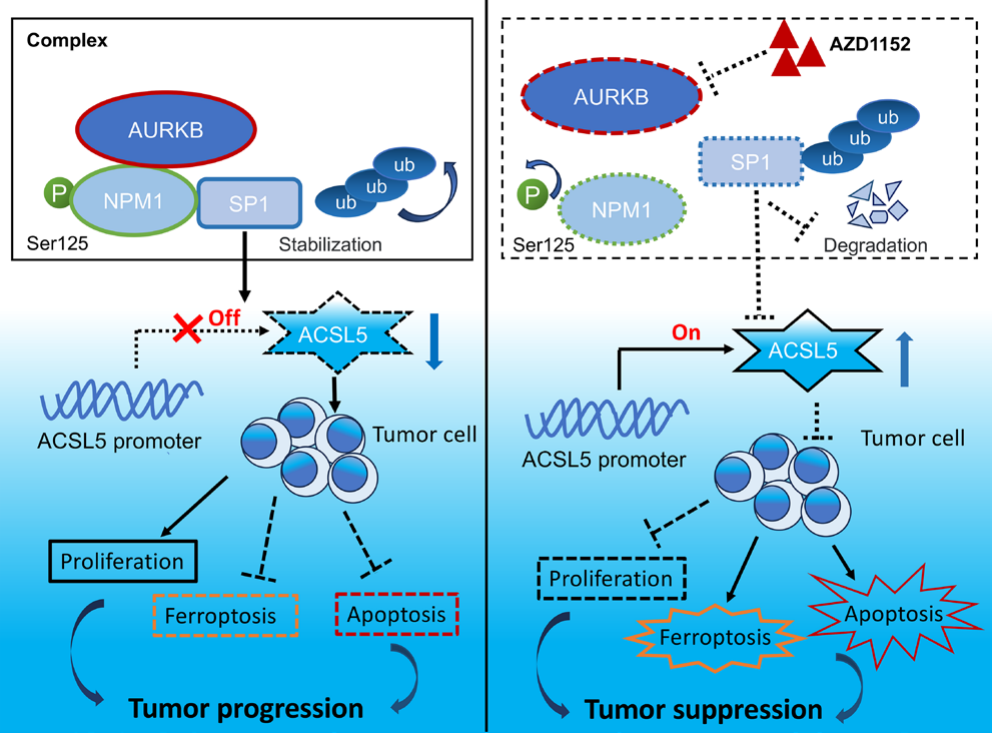
Illustration of the oncogenic pathway regulated by AURKB in RMS.

In conclusion, the present study comprehensively assessed the oncogenic role of AURKB in RMS. Phosphorylation of NPM1 at Ser125 by AURKB stabilizes SP1, thus decreasing the expression of ACSL5 and promoting the apoptosis and ferroptosis resistance of RMS cell. In another word, AURKB inhibition induces apoptosis and ferroptosis through the NPM1/SP1/ACSL5 axis in RMS (Figure 10). Therefore, AURKB serves as a potential prognostic biomarker and therapeutic target in the treatment of RMS, particularly when combined with VCR.

## Discussion

Despite a growing understanding of the oncogenic mechanism of RMS, there is still a lack of new candidate drug targets. Previous studies revealed that AURKB is overexpressed in various human cancers, and high expression of AURKB is associated with a poorly differentiated phenotype, enhancing cell proliferation, and lymph node metastasis (16, 24–26). High expression of AURKB is positively associated with poor outcomes (17). However, little is known about the clinical significance of AURKB in RMS. In this study, AURKB was overexpressed and high expression of AURKB was reported to be positively correlated with poor survival outcomes in RMS.

After clinical samples were analyzed, models were utilized to search for further evidence. Knockdown of AURKB or pharmacological inhibition of AURKB with AZD1152 significantly decreased proliferation and induced apoptosis and ferroptosis of RMS cells in vitro and in vivo, but the exact mechanism remains unclear. Jiang et al. revealed that AURKB results in the phosphorylation of PKM2 at T45, which is required for the interaction of PKM2 with myosin light chain 2 during cytokinesis (27). This team also found that AURKB phosphorylated c-Myc at S67, enhancing T cell leukemogenesis (18). In addition, knockdown of AURKB can reduce the phosphorylation level of NPM1 at Ser125, thus promoting cytokinesis in HEK293 and NIH3T3 (28). Similarly, AURKB was found to phosphorylate NPM1 at Ser125 in our study, which was required for the interaction of NPM1 with SP1, leading to apoptosis and ferroptosis resistance of RMS cells. This is the first report to our knowledge to reveal the apoptosis and ferroptosis resistance role of AURKB in RMS cells by regulating the phosphorylation of NPM1 at Ser125.

SP1 is a well-known member of the transcription factor family, which is implicated in plenty of essential biological processes and crucial in regulating cancer cell proliferation, apoptosis, ferroptosis, and drug resistance (29–31). SP1 was reported to transcriptionally regulate fibroblast growth factor receptor 4 and act as an oncogene in RMS (32). According to our study, SP1 was further revealed to be stabilized by the AURKB/NPM1 axis through the ubiquitin-proteasome system, modulating apoptosis and ferroptosis resistance in RMS. ACSL5 mainly acts as a tumor suppressor in a variety of cancers, with an unclear role in RMS (33). Our findings revealed for the first time to our knowledge that ACSL5 induced the apoptosis and ferroptosis in RMS, which was transcriptionally suppressed by SP1.

AZD1152, a highly selective AURKB inhibitor, is under multiple clinical trials; however, results demonstrate that AZD1152 alone may not produce durable clinical responses (34, 35). Combination therapy may be more effective. In the present study, the anti-RMS effect of VCR was augmented when combined with AZD1152, which was consistent with previous studies. Ikezoe et al. demonstrated that VCR activates AURKB by increasing the level of p-AURKB, which is reversed by AZD1152 (36). In our study, however, the molecular mechanism by which AZD1152 synergizes VCR in RMS was that VCR could significantly reduce the expression of p-AURKB. The possible reason for this discrepancy might be due to the synergistic mechanisms of these two drugs, which vary from tumor to tumor.

As a fusion gene found in alveolar rhabdomyosarcoma (ARMS), the PAX3-FOXO1 fusion occurs in about 60% of patients with ARMS, causing the 5-year survival rate of less than 30%–50% for patients with this fusion-positive RMS (FP-RMS) (37). This underscores the critical need to explore targeted therapies aimed at PAX3-FOXO1, a transcriptional activator that affects multiple oncogenic pathways in RMS (37). It was reported that aurora kinase A (AURKA) inhibition can destabilize PAX3-FOXO1 and induce RMS cell death (38). Similarly, whether AURKB could perform similar functions in modulating the PAX3-FOXO1 fusion gene is an interesting question worth exploring here, as it is another kind of aurora kinase that exerts important roles in cell cycle regulation. In our present study, we also analyzed the association between AURKB expression level and RMS classification based on the fusion gene type. We observed a worse prognosis of patients with FP-RMS than that of the patients with fusion-negative RMS (FN-RMS) (Supplemental Figure 9A). In patients with FN-RMS, the high AURKB expression level was also related to a poor prognosis (Supplemental Figure 9B). However, in the patients with FP-RMS, there was no significant difference in survival between the high and low AURKB expression groups (Supplemental Figure 9C). A possible reason for this could be the limited number of FP-RMS cases available for analysis in this study.

Furthermore, we treated the ARMS cell lines with the AURKB inhibitor AZD1152 or through AURKB genetic knockdown and found that both pharmacological inhibition of AURKB and genetic suppression of AURKB resulted in substantial downregulation of this fusion gene (Supplemental Figure 10). This suggests that AURKB may regulate PAX3-FOXO1 fusion gene expression, indicating that AURKB inhibition could be a potential targeted therapy for PAX3-FOXO1 fusion gene–positive RMS. However, the precise mechanisms by which AURKB regulates the expression and stability of the PAX3-FOXO1 fusion gene need to be discussed in depth and require further investigation through additional experiments.

Considering the unsatisfactory results of AZD1152 in clinical trials (34, 35, 39), novel dosage forms of AURKB inhibitor are still needed for further exploration. In recent years, many clinical trials have been conducted on aurora kinase inhibitors, including pan-selective aurora kinase inhibitors, AURKA inhibitors, and AURKB inhibitors. Among them, CS2164 is a novel AURKB inhibitor that has entered a phase II clinical trial in recent years, which may help to provide more AURKB-targeted treatment strategies (40) that need exploration in the future.

## Methods

### Sex as a biological variable.

Sex was not considered as a biological variable in this study, as experiments were only performed in female mice. Female mice were selected, as they are more docile during experimental procedures.

### Bioinformatics analysis.

The original data were obtained from the GEO dataset GSE108022 (https://www.ncbi.nlm.nih.gov/geo/). The clusterProfiler package in R software was used to perform KEGG analysis. The STRING (https://string-db.org/) tool and Cytoscape software were used for filtering hub genes to obtain genes of high connectivity. Detailed information for bioinformatics analysis can be found in the Supplemental Methods.

### Cell culture.

The human RMS cell lines RD (FN) and RH30 (FP) (42) were obtained from the ATCC, and RH28 (FP) was obtained from the Guandao Biological Engineering Co. LTD. The human RMS cell lines A204 (FN) and the normal HSKMCs were purchased from Jinio Biological Science and Technology LTD. The human RMS cells were cultured in recommended medium supplemented with 10% FBS and 1% antibiotics. For the HSKMCs, cells were cultured in DMEM medium with 10% FBS and 1% antibiotics. After reaching about 80%–90% confluency, myoblasts were differentiated in DMEM with 2% horse serum (16050122, Gibco) for about 4 days (43). All cells were cultured in a humidified 5% CO_2_ incubator at 37°C.

### Tissue sample collection and ethics.

A total of 98 paraffin-embedded RMS samples were collected for this study. Detailed information can be found in the Supplemental Methods.

### IHC assay.

The IHC assay was performed following the methods of a previous study (41). Anti-AURKB mouse monoclonal antibody (Santa Cruz) and goat anti-mouse secondary antibody (ZSGB BIO) were used. Detailed information on IHC can be found in the Supplemental Methods.

### RNA isolation and qRT-PCR.

Total RNA was extracted from the cultured cells with TRIzol reagent (Takara). Then, 0.5 μg total RNA was reversely transcribed into cDNA using the PrimeScript RT Master Mix (Takara), followed by qRT-PCR using primers listed in Supplemental Table 1. Three replicates of each sample were amplified in a 10 μL reaction mixture using the SYBR Green Kit (Dongsheng Biotech).

### Cell counting kit-8 assay.

Cells were seeded in 96-well plates and added with Cell counting kit-8 (CCK-8) solution (APExBIO) in 3 replicated wells of each sample, followed by incubation for 2.5 hours at 37°C in a 5% CO_2_ humidified atmosphere. Spectrophotometry was performed in each well at 450 nm.

### Colony formation assay.

Three thousand cells were cultured in a 6-well plate. About 10–14 days later, colonies were fixed in 4% polyformaldehyde and stained with 0.1% crystal violet. After staining for about 15 minutes, the colonies were washed with tap water and then dried in the air. The images for these colonies were taken by a camera, and the corresponding numbers were quantified by Image J (NIH) software.

### Cell apoptosis analysis.

Cell apoptosis was analyzed using the Annexin V–Alexa Fluor 647/7–AAD Apoptosis Detection Kit (4A Biotech Co. Ltd.). Detailed information regarding this procedure can be found in the Supplemental Methods.

### WB and mass spectrometry analysis.

The WB assay was performed following methods described in a previous study (41). AURKB (3094, Cell Signaling Technology), NPM1 (60096-1-Ig, Proteintech), SP1 (10306-1-AP, Proteintech), and homologous immunoglobulin G (IgG, 3900, Cell Signaling Technology) antibodies were used for immunoprecipitation analysis. Detailed information regarding this experiment can be found in the Supplemental Methods.

### Xenograft tumorigenesis in vivo.

Indicated RH30 cells were preadjusted to viable cells using trypan blue dye, and then RH30 cells with a density of 5 × 10^6^ cells per mouse were inoculated into the right flank of the female nude mice at 3–4 weeks of age. Detailed information was provided in the Supplemental Methods.

### Detection of MDA level.

Cells were gently collected in PBS solution with a cell spatula. The MDA concentration in cell lysates was assessed with the MDA detection Kit (A003-4–1, Jiancheng) according to the manufacturer’s instructions. Detailed information regarding MDA detection can be found in the Supplemental Methods.

### Detection of cellular lipid ROS level.

The cellular lipid ROS level was detected with the flow cytometer using the BODIPY-581/591 C11 kit (D3861, Thermo Fisher Scientific). Detailed information regarding lipid ROS detection can be found in the Supplemental Methods.

### Iron assay.

The cellular iron was detected with the Iron Assay Kit (BC5415, Solarbio) following the manufacturer’s instructions. The standard samples were diluted based on the protocol, and the absorbance was measured at 593 nm. The standard curves were plotted based on which the cellular iron concentration in each sample was calculated.

### Ubiquitination assay.

RMS cells with or without AURKB or NPM1 knockdown were treated with 10 μM MG132 for 10 hours. Lysates were incubated with anti-SP1 (10306-1-AP, Proteintech) antibody, and each lysate was added with an equivalent amount of beads (Thermo Fisher Scientific) and incubated overnight at 4°C. Reactions were subjected to WB analysis. Whole-cell lysates served as input control, and normal IgG acted as a negative control.

### Statistics.

Data analysis was carried out using GraphPad Prism 8 or SPSS statistics software (version 22). Continuous variables were expressed as mean ± SD, and their differences were compared using 2-tailed Student’s *t* test or 1- or 2-way ANOVA. The differences in categorical variables were compared using χ^2^ test. Survival rates were calculated by the Kaplan-Meier method and compared using log-rank test. All tests were 2-sided, and differences were considered significant if *P* < 0.05.

### Study approval.

This study was approved by the Ethics Committee of Sun Yat-sen University Cancer Center (B2023-187-01). The animal experiments were approved by the Animal Ethics Committee of Sun Yat-sen University (SYSU-IACUC-2022-001988).

### Data availability.

The datasets in the present study are available from the corresponding author under reasonable request. Values for data points in graphs are reported in the Supporting Data Values file.

## Author contributions

HC provided conceptualization; data curation; formal analysis; methodology; project administration; software; validation; visualization; writing of the original draft; and review and editing of the manuscript. ML provided conceptualization; data curation; formal analysis; methodology; project administration; software; supervision; validation; and review and editing of the manuscript. Yu Zhang provided investigation; methodology; supervision; visualization; and review and editing of the manuscript. MS provided investigation; supervision; and visualization. YQ and JW provided software; supervision; and visualization. FS, JZ, and JH provided methodology; software; and visualization. JL and JX provided software and visualization. SL provided funding acquisition; methodology; resources; supervision; validation; and review and editing of the manuscript. Yizhuo Zhang provided conceptualization; funding acquisition; methodology; resources; supervision; validation; and review and editing of the manuscript.

## Supplementary Material

Supplemental data

Unedited blot and gel images

Supporting data values

## Figures and Tables

**Table 1:**
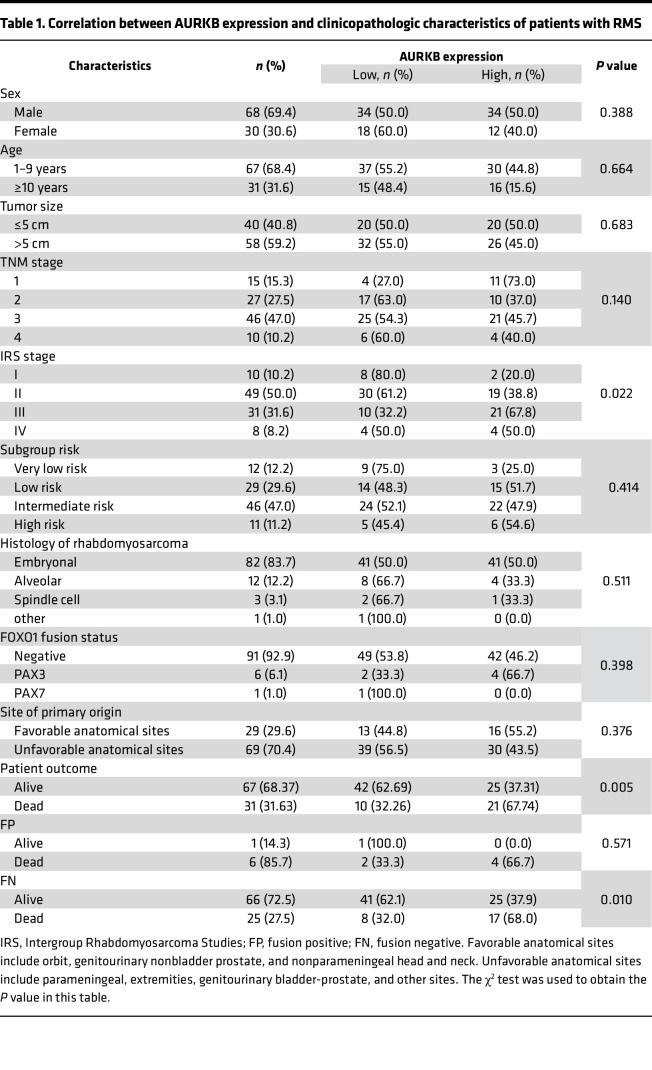
Correlation between AURKB expression and clinicopathologic characteristics of patients with RMS

## Version 1 02/10/2025

Electronic publication

## References

[1] Skapek SX (2019). Rhabdomyosarcoma. Nat Rev Dis Primers.

[2] Yechieli RL (2021). Rhabdomyosarcoma. Pediatr Blood Cancer.

[3] Dziuba I (2018). Rhabdomyosarcoma in children - current pathologic and molecular classification. Pol J Pathol.

[4] Hanahan D, Weinberg RA (2000). The hallmarks of cancer. Cell.

[5] Zhang C (2022). Ferroptosis in cancer therapy: a novel approach to reversing drug resistance. Mol Cancer.

[6] Zhao L (2022). Ferroptosis in cancer and cancer immunotherapy. Cancer Commun (Lond).

[7] Jiang X (2021). Ferroptosis: mechanisms, biology and role in disease. Nat Rev Mol Cell Biol.

[8] Lei G (2022). Targeting ferroptosis as a vulnerability in cancer. Nat Rev Cancer.

[9] Bolanos-Garcia VM (2005). Aurora kinases. Int J Biochem Cell Biol.

[10] Gully CP (2010). Antineoplastic effects of an Aurora B kinase inhibitor in breast cancer. Mol Cancer.

[11] Bertran-Alamillo J (2019). AURKB as a target in non-small cell lung cancer with acquired resistance to anti-EGFR therapy. Nat Commun.

[12] Nie M (2020). AURKB promotes gastric cancer progression via activation of CCND1 expression. Aging (Albany NY).

[13] Wang Z (2020). AURKB promotes the metastasis of gastric cancer, possibly by inducing EMT. Cancer Manag Res.

[14] Nikonova AS (2013). Aurora A kinase (AURKA) in normal and pathological cell division. Cell Mol Life Sci.

[15] Al-Khafaji AS (2017). Aurora B expression modulates paclitaxel response in non-small cell lung cancer. Br J Cancer.

[16] Azzariti A (2011). Aurora B kinase inhibitor AZD1152: determinants of action and ability to enhance chemotherapeutics effectiveness in pancreatic and colon cancer. Br J Cancer.

[17] Portella G (2011). Aurora B: a new prognostic marker and therapeutic target in cancer. Curr Med Chem.

[18] Jiang J (2020). Direct phosphorylation and stabilization of MYC by Aurora B kinase promote T-cell leukemogenesis. Cancer Cell.

[19] Pardieu B (2022). Cystine uptake inhibition potentiates front-line therapies in acute myeloid leukemia. Leukemia.

[20] Pitts HA (2023). SPINK2 protein expression is an independent adverse prognostic marker in AML and is potentially implicated in the regulation of ferroptosis and immune response. Int J Mol Sci.

[21] Xie Y (2022). Cytotoxic effects of the biflavonoids isolated from Selaginella trichoclada on MCF-7 cells and its potential mechanism. Bioorg Med Chem Lett.

[22] Yang WH (2019). The Hippo pathway effector TAZ regulates ferroptosis in renal cell carcinoma. Cell Rep.

[23] Huang H (2022). Effective prediction of potential ferroptosis critical genes in clinical colorectal cancer. Front Oncol.

[24] Bertran-Alamillo J (2019). AURKB as a target in non-small cell lung cancer with acquired resistance to anti-EGFR therapy. Nat Commun.

[25] Zhang J (2020). Aurora B induces epithelial-mesenchymal transition by stabilizing Snail1 to promote basal-like breast cancer metastasis. Oncogene.

[26] Sorrentino R (2005). Aurora B overexpression associates with the thyroid carcinoma undifferentiated phenotype and is required for thyroid carcinoma cell proliferation. J Clin Endocrinol Metab.

[27] Jiang Y (2014). PKM2 phosphorylates MLC2 and regulates cytokinesis of tumour cells. Nat Commun.

[28] Shandilya J (2014). Phosphorylation of multifunctional nucleolar protein nucleophosmin (NPM1) by aurora kinase B is critical for mitotic progression. FEBS Lett.

[29] Beishline K, Azizkhan-Clifford J (2015). Sp1 and the ‘hallmarks of cancer. ’ FEBS J.

[30] Zhang Q (2021). Sp1-mediated upregulation of Prdx6 expression prevents podocyte injury in diabetic nephropathy via mitigation of oxidative stress and ferroptosis. Life Sci.

[31] Ma L (2021). LSD1-Demethylated LINC01134 confers oxaliplatin resistance through SP1-induced p62 transcription in HCC. Hepatology.

[32] Yu SJ (2004). Sp1-mediated transcriptional control of fibroblast growth factor receptor 4 in sarcomas of skeletal muscle lineage. Clin Cancer Res.

[33] Quan J (2021). ACSL family: The regulatory mechanisms and herapeutic implications in cancer. Eur J Pharmacol.

[34] Schwartz GK (2013). Phase I study of barasertib (AZD1152), a selective inhibitor of Aurora B kinase, in patients with advanced solid tumors. Invest New Drugs.

[35] Lowenberg B (2011). Phase 1/2 study to assess the safety, efficacy, and pharmacokinetics of barasertib (AZD1152) in patients with advanced acute myeloid leukemia. Blood.

[36] Ikezoe T (2009). Analysis of Aurora B kinase in non-Hodgkin lymphoma. Lab Invest.

[37] David M (2021). FOXF1 is required for the oncogenic properties of PAX3-FOXO1 in rhabdomyosarcoma. Oncogene.

[38] Johannes O (2020). Aurora A Kinase inhibition destabilizes PAX3-FOXO1 and MYCN and synergizes with navitoclax to induce rhabdomyosarcoma cell death. Cancer Res.

[39] Boss DS (2011). Clinical evaluation of AZD1152, an i.v. inhibitor of Aurora B kinase, in patients with solid malignant tumors. Ann Oncol.

[40] Jing XL, Chen SW (2021). Aurora kinase inhibitors: a patent review (2014–2020). Expert Opin Ther Pat.

[41] Li M (2021). ISL1 promoted tumorigenesis and EMT via Aurora kinase A-induced activation of PI3K/AKT signaling pathway in neuroblastoma. Cell Death Dis.

[42] Silvia P (2021). Interaction between SNAI2 and MYOD enhances oncogenesis and suppresses differentiation in Fusion Negative Rhabdomyosarcoma. Nat Commun.

[43] Jiang X, J et al (2023). Pyruvate dehydrogenase B regulates myogenic differentiation via the FoxP1-Arih2 axis. J Cachexia Sarcopenia Muscle.

