# Supplementary material for: AURKB inhibition induces rhabdomyosarcoma apoptosis and ferroptosis through NPM1/SP1/ACSL5 axis
Source: JCI Insight. 2025 Feb 10;10(3):e182429. doi: 10.1172/jci.insight.182429 (PMC11948576; doi:10.1172/jci.insight.182429)
Supplement: Supplemental data [file jciinsight-10-182429-s221.pdf]

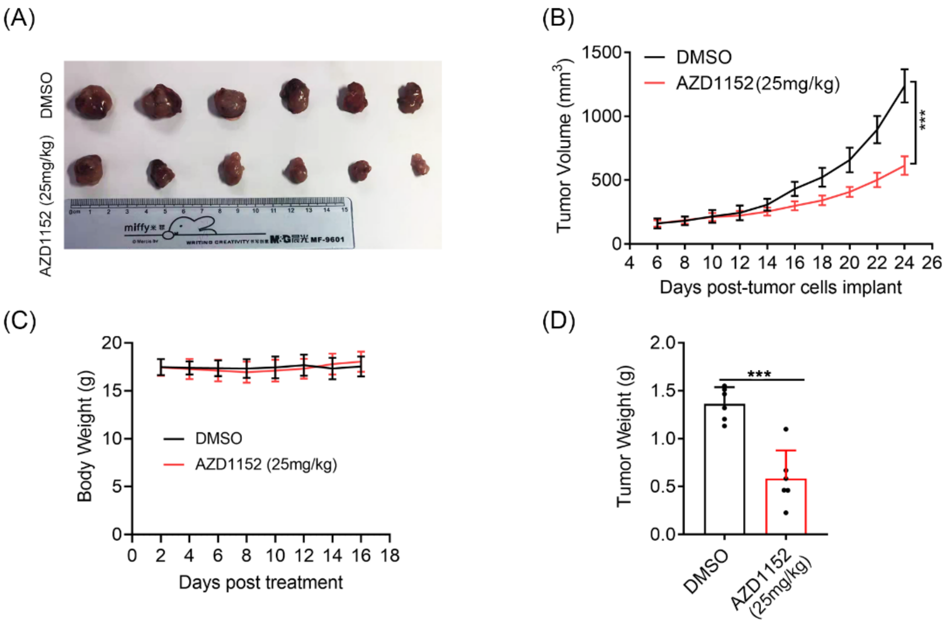

Supplementary Figure 1. Inhibition AURKB with AZD1152 exerted anti-RMS effect in vivo. (A) Photographs of harvested tumors of RH30 tumor-bearing mice. (B) Tumor volumes of RH30 xenograft models with or without AZD1152 treatment. (C) Body weight of mice from different groups measured every two days. (D) Tumor weight of independent groups was recorded at the endpoint of the experiment. Data are shown as mean ± SD of six mice in each group. ***$P < 0.001$.

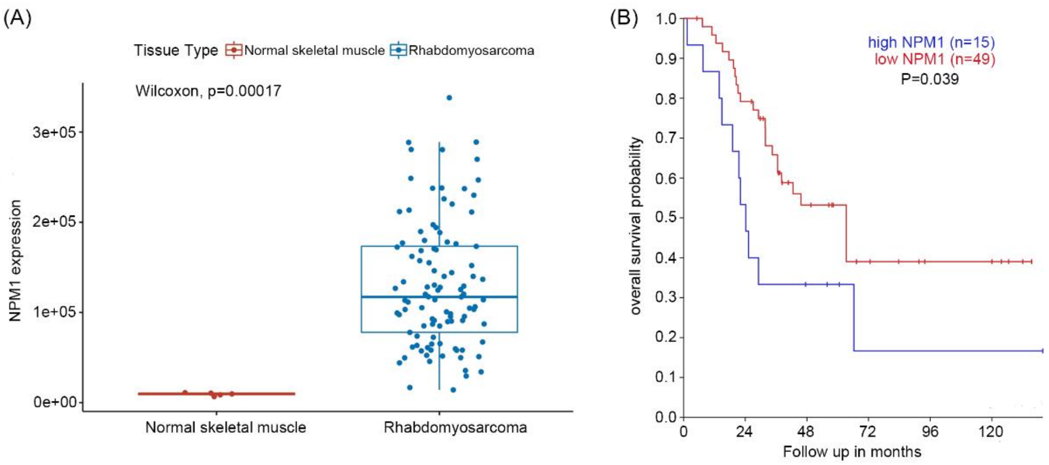

Supplementary Figure 2. NPM1 was upregulated in RMS and associated with poor outcome in RMS patients. (A) NPM1 was upregulated in RMS based on the GSE108022 database. (B) K-M plotting analysis of NPM1 from R2 online platform.

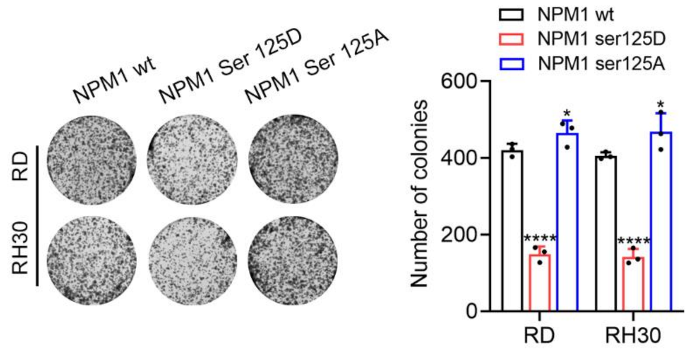

Supplementary Figure 3. The status of NPM1 p-Ser125 was important for RMS cell proliferation. Colony formation assay of RD and RH30 with different status of NPM1 p-Ser125. *P < 0.05, ****P < 0.0001.

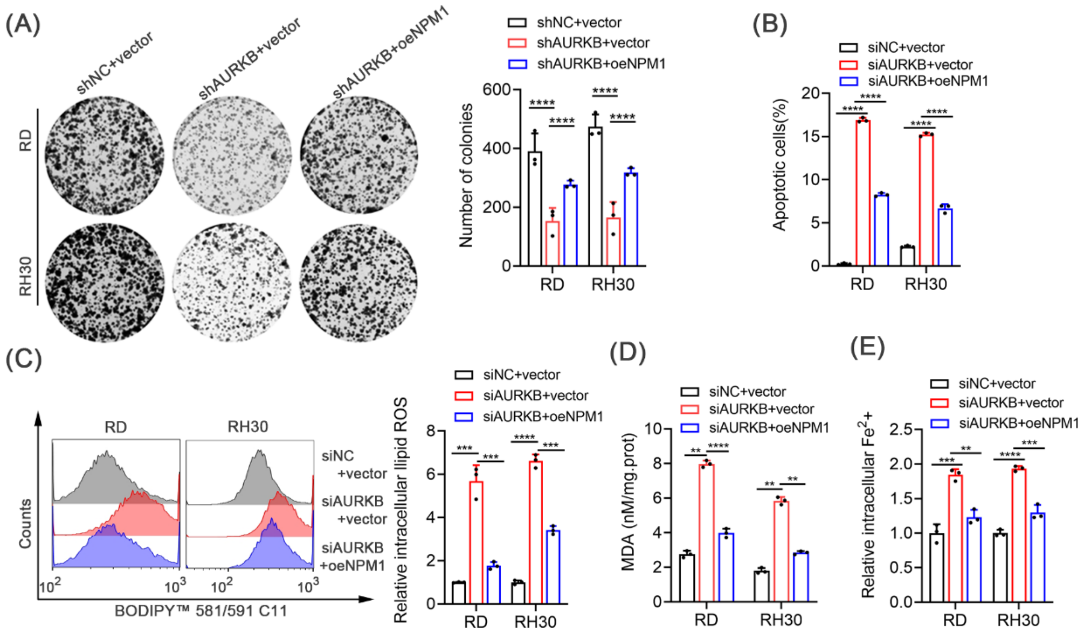

Supplementary Figure 4. The regulation of by AURKB on RMS cell proliferation, apoptosis and ferroptosis was dependent on NPM1. (A) The colony formation assay indicated cell proliferation ability in shAURKB-RMS cells with or without re-introduction of NPM1. (B) Apoptosis assay by flow cytometer in siAURKB-RMS cells with or without re-introduction of NPM1. (C) Intracellular ROS level detected in siAURKB-RMS cells with or without re-introduction of NPM1. (D) Intracellular lipid ROS level detected in siAURKB-RMS cells with or without re-introduction of NPM1. (E) Intracellular Fe2+ level detected in siAURKB-RMS cells with or without re-introduction of NPM1. $^{**}P < 0.01$, $^{***}P < 0.001$, $^{****}P < 0.0001$.

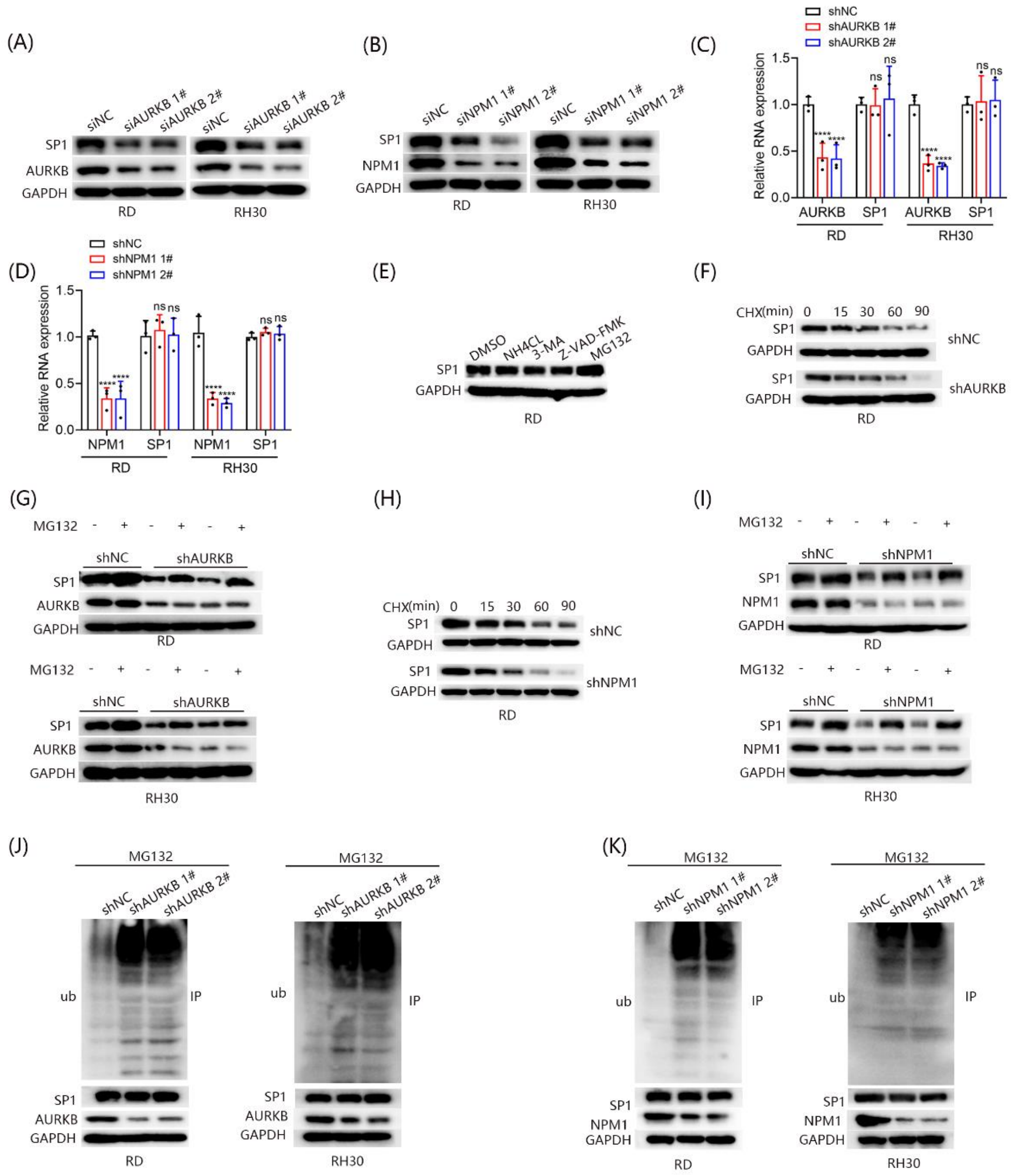

Supplementary Figure 5. AURKB and NPM1 stabilized SP1 through inhibiting its ubiquitination. (A) Detection of SP1 protein levels with AURKB knockdown. (B) Detection of SP1 protein levels with NPM1 knockdown. (C) Detection of SP1 mRNA levels with AURKB knockdown. (D) Detection of SP1 mRNA levels with NPM1 knockdown. (E) The protein level of SP1 after treated with 10 μM of the lysosome inhibitor NH4Cl and 3-MA, the caspase inhibitor Z-VAD-FMK, and the proteasome inhibitor MG132 for 10 h in RD cells, and then cell lysates were analyzed by WB with indicated antibodies. (F) Time-course analysis of SP1 protein levels in AURKB-depleted RD cells. (G) AURKB-depleted RD and RH30 cells were treated with MG132

(10 μM) for 10 h before harvest. AURKB and SP1 were analyzed by immunoblot, with GAPDH as a loading control. (H) Time-course analysis of SP1 protein levels in NPM1-depleted RD cells. (I) NPM1-depleted RD and RH30 cells were treated with MG132 (10 μM) for 10 h before harvest. NPM1 and SP1 were analyzed by immunoblot, with GAPDH as a loading control. (J, K) RD and RH30 cells were co-transfected with the shNC or shAURKB or shNPM1, and cell lysates were subjected to IP with SP1 antibody, followed by WB with indicated antibodies. Cells treated with 10 μM MG132 for 10 h. ns: non significance, ****$P < 0.0001$.

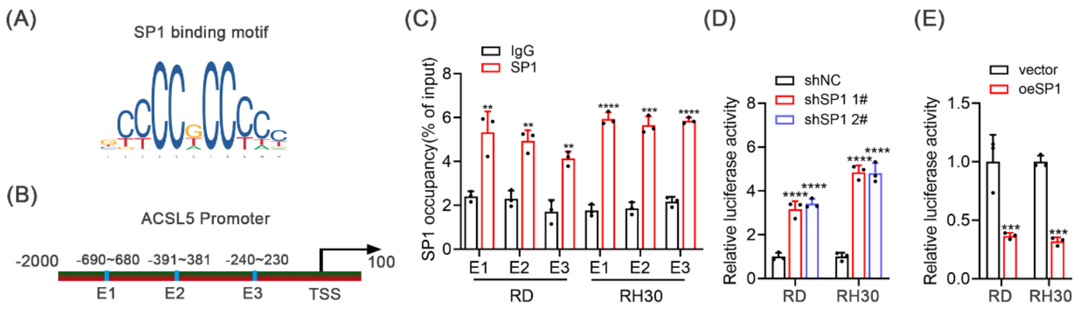

Supplementary Figure 6. The transcriptional regulation of SP1 on ACSL5.

(A) The SP1 binding motif predicted by JASPAR (https://jaspar.genereg.net/analysis).

(B) The transcription initiation site (TSS) site of SP1 at the ACSL5 promoter region.

(C) ChIP-qPCR analysis revealed the enrichment of SP1 at the ACSL5 promoter region.

(D, E) Dual-luciferase reporting experiment confirmed that SP1 could transcriptionally suppress ACSL5. **P < 0.01, ***P < 0.001, ****P < 0.0001.

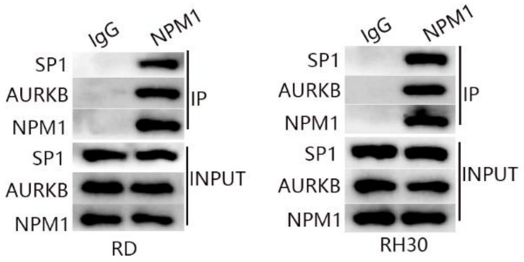

Supplementary Figure 7. NPM1 interacted with AURKB and SP1 in RMS.

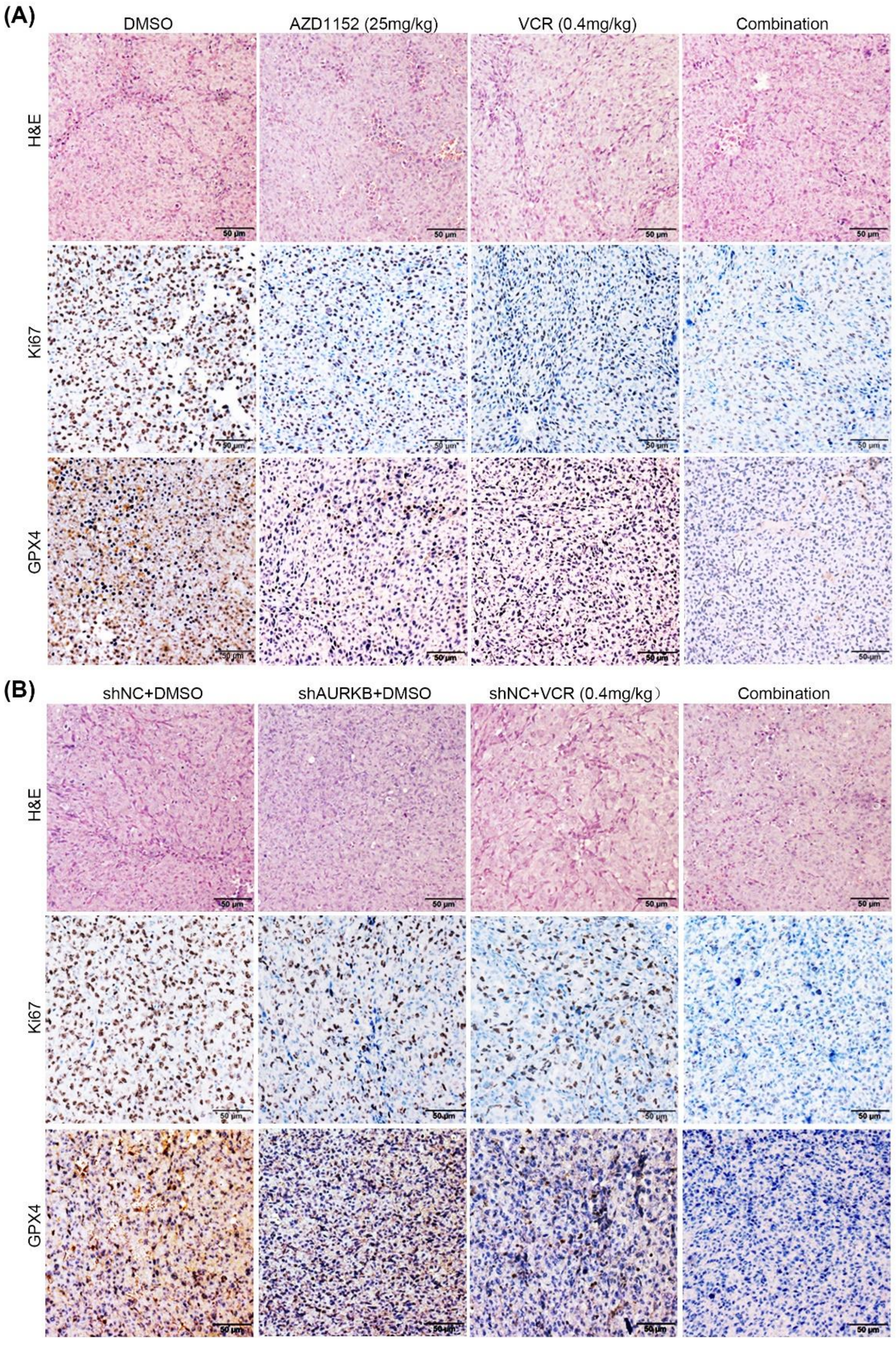

Supplementary Figure 8. H&E and IHC staining of Ki67 and GPX4 with indicated treatment. (A) H&E and IHC staining of Ki67 and GPX4 after treatment with DMSO, AZD1152, VCR and combination. (B) H&E and IHC staining of Ki67 and GPX4 with indicated treatment.

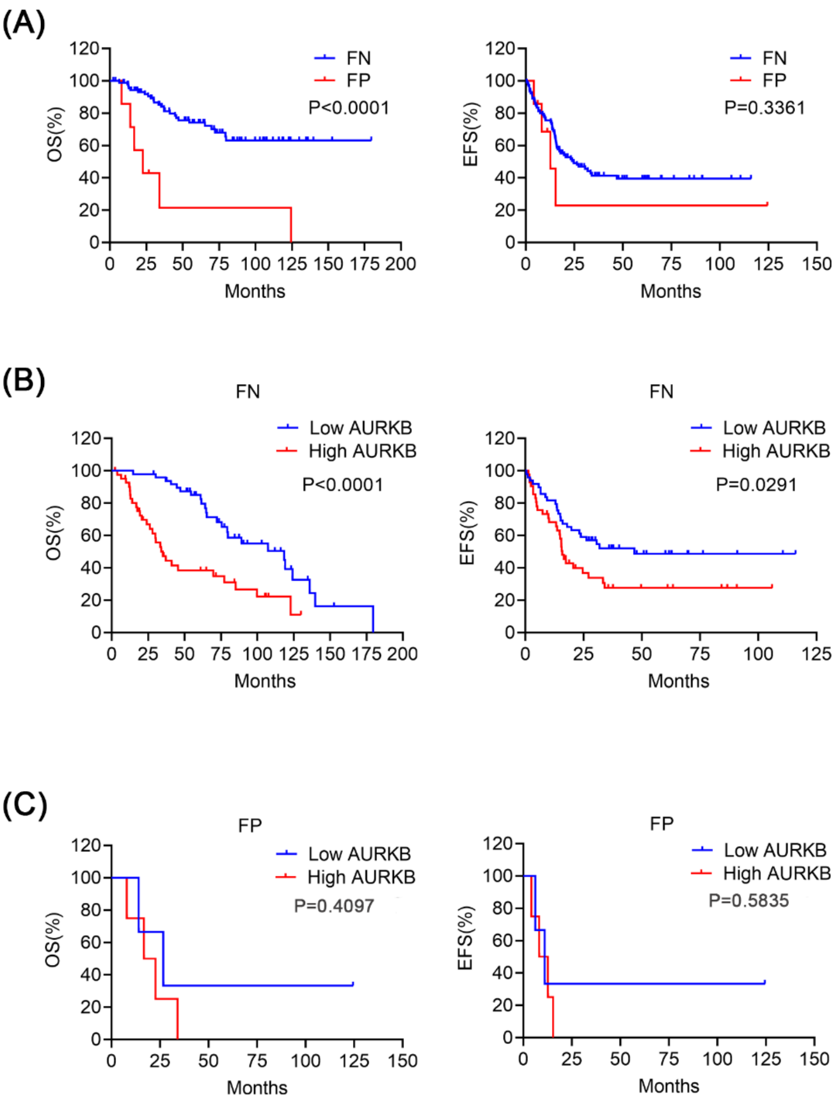

Supplementary Figure 9. Survival analysis of AURKB expression in relation to FOXO1 fusion gene types in RMS patients from our cohort.

(A) Overall survival (OS) and event-free survival (EFS) analysis of fusion-negative (FN) and fusion-positive (FP) RMS patients. (B) OS and EFS analysis specifically for FN RMS patients. (C) OS and EFS survival curves for FP RMS patients.

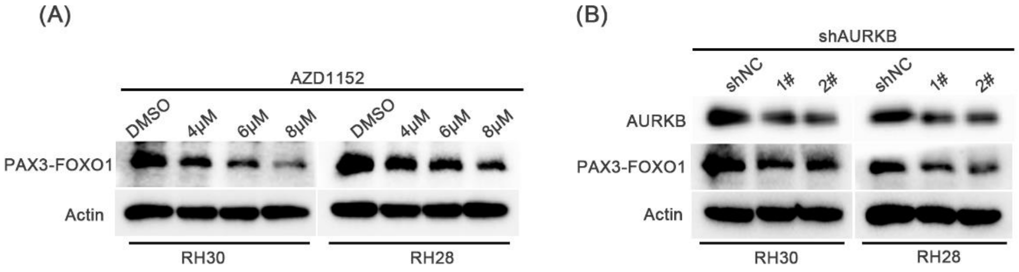

Supplementary Figure 10. Changes in FOXO1 fusion protein levels after AURKB inhibition.

(A) The western blot (WB) analysis performed on two FP RMS cells treated with different concentrations of AZD1152 after 48 h. (B) WB analysis of two FP RMS cell lines with AURKB knockdown using shRNA.

Supplementary Table 1. Primers for qRT-PCR.

Genes	Forward primer (5'->3')	Reverse primer (5'->3')
AURKB	GACCTAAAGTTCCCCGCTTC	GACAGATTGAAGGGCAGAGG
ACSL5	TTTGGAGCTGCCATCTTCTT	ACAGCGAGTCCTCTTTGGAA
EMP1	CCCTTCTGGTCTTGGATGAA	GGAGGTGGACCCCTTATGAT
NPM1	CCCTTCTGGTCTTGGATGAA	GGAGGTGGACCCCTTATGAT
EPAS1	GGCTTTTTGCCATCTGTGAT	GCACTTGAAGGGCTAGCAAC
SP1	AGGGCTGCAAGTAGTGAGGA	TTCACTGGGAGAAAGGGATG

Supplementary Table 2. The 5 overlapped transcription factor regulating ACSL5 predicated by Animal TFDB database.

TF	Score	P-value	Q-value
IRF1	14.2078	7.89E-6	0.00297
JUN	13.0517	3.10E-5	0.12900
BRD4	11.8462	3.28E-5	0.00671
SP1	17.427	5.93E-7	0.00235
SOX2	12	3.26E-5	0.00670

Supplementary Table 3. CI of AZD1152 combined first-line chemotherapy agents for RD.

RD	ED50	ED75	ED90	ED95
AZD1152 + Vincristine	0.37648	0.19517	0.13116	0.11723
AZD1152 + Doxorubicin	0.41881	0.56428	0.76278	0.93823
AZD1152 + Actinomycin D	0.52038	0.39710	0.56084	0.92426

CI: Combination index

Supplementary Table 4. CI of AZD1152 combined first-line chemotherapy agents for RH30.

RH30	ED50	ED75	ED90	ED95
AZD1152+ Vincristine	0.18817	0.19247	0.33707	0.53598
AZD1152 + Doxorubicin	0.50262	0.42150	0.39544	0.38491
AZD1152 + Actinomycin D	0.29951	0.29223	0.46798	0.69212

CI: Combination index

Supplementary Methods

Bioinformatics analysis. The original data were obtained from GSE108022 dataset in GEO (https://www.ncbi.nlm.nih.gov/geo/). A total of 106 samples were screened, including 101 samples of RMS tissues and 5 samples of normal striated muscle tissues. The absolute value of log2 fold change (FC) was set to > 3, and the cutoff value was adjusted to a P value of < 0.001. The FDR and P values were screened to obtain all differentially expressed genes (DEGs). The clusterProfiler package in R software was used to perform KEGG analysis. The STRING (https://string-db.org/) tool and Cytoscape software were used for filtering hub genes to obtain genes of high connectivity.

Tissue sample collection and ethics. A total of 98 paraffin-embedded RMS samples were collected for this study. The tissue specimens were obtained prior to the initial treatment. RMS was diagnosed consistently by two certified pathologists. Patients who received systematic treatment and completed follow-up were enrolled. OS was defined as the time interval between diagnosis and death from any cause, and event-free survival (EFS) was defined as the time interval between diagnosis and disease progression, relapse, or death from any cause. This study was approved by the Ethics Committee of Sun Yat-sen University Cancer Center (Number: B2023-187-01).

Immunohistochemistry (IHC) assay. The IHC assay were performed in the 98 tissue samples following the operation in the previous study [18]. Anti-AURKB mouse monoclonal antibody (Santa Cruz) and goat anti-mouse secondary antibody (ZSGB BIO, China) were used. The results were evaluated based on the staining intensity and the proportion of positive-staining tumor cells. The staining intensity was scored as follows: 0 points - negative; 1 point - weak; 2 points - moderate; 3 points - strong. The positive cells were scored as follows: 0 points - less than 25%; 1 point - 25%-50%; 2 points - 50%-75%; 3 points - 75%-100%. The expression level was assessed based on

the composite score (the product of the above two scores): 0-1 point as low expression, 2-3 points as high expression.

Cell apoptosis and cell cycle analysis. The cells were harvested 48 h after different treatments. Then cell apoptosis was analyzed using the Annexin V-Alexa Fluor 647/7-AAD Apoptosis Detection Kit (4A Biotech Co., Ltd.). Some of these cells were fixed in 70% ethanol at 4°C overnight. Then they were washed, and stained with 50 mg/mL propidium iodide (PI) in solution containing 2 mg/mL RNase. Finally, flow cytometry was performed on the cells (SP6800, Sony, Japan).

Western blotting (WB) and Mass Spectrometry Analysis. The cells were washed in PBS and lysed for 30 min on ice with RIPA buffer (Beyotime, Shanghai, China) containing protease inhibitor cocktail (TargetMol, USA). BCA Assay Kit (Thermo Scientific, USA) was used for calculating the protein concentration. An equal volume of total protein was resuspended in loading buffer and denatured at 98°C for 10 min. The protein was separated by SDS-PAGE, and transferred onto a polyvinylidene fluoride membrane (Merck, USA). Then the membrane was blocked in 5% fat-free milk, and incubated with primary antibodies and goat anti-rabbit secondary antibodies subsequently. In immunoprecipitation analysis, the cells were lysed in buffer containing EDTA-free protease inhibitor cocktail. Then, 1 mg of the cell lysates were incubated with the corresponding AURKB (Cell Signaling Technology, #3094), NPM1 (Proteintech, 60096-1-Ig), SP1 (Proteintech,10306-1-AP), and homologous immunoglobulin G (IgG, Cell Signaling Technology, #3900) antibodies overnight at 4°C. Finally, each lysate was added with an equivalent amount of beads (ThermoFisher Scientific) and incubated overnight at 4°C. Next, beads were washed with lysis buffer for three times. Reactions were subjected to WB analysis. Whole cell lysates served as input control, and normal IgG acted as a negative control. Proteins were separated by sodium dodecyl sulfate-polyacrylamide gel electrophoresis (SDS-PAGE), then the gel was silver staining. The distinct bands were cut for mass spectrometry analysis subsequently.

Detection of cellular ROS level. The cellular ROS level was quantified by a flow cytometer using DCFH-DA kit (Beyotime, S0033S) according to the manufacturer's instructions. To be brief, RMS cells were incubated with DCFH-DA at a working concentration of 10 μM for 20 min at 37°C.Then they were washed with FBS-free medium and detected by flow cytometry (SP6800, Sony, Japan).

Detection of cellular lipid ROS level. The cellular lipid ROS level was detected with the flow cytometer using BODIPY-581/591 C11 kit (D3861, Thermo Fisher Scientific). The cells were incubated with medium containing BODIPY-581/591 C11 at a working concentration of 10 μM for 30 min at 37 °C and 5% CO_2. Then they were collected by trypsin without EDTA and washed with PBS, followed by flow cytometry (SP6800, Sony, Japan).

ChIP assay

Indicated cells were fixed with 1% formalin and sonicated to shear the DNA into fragments at 200-1000 bp, and then sheared DNA was incubated with antibodies against SP1 (Cell Signaling Technology) or IgG control (Santa Cruz Biotechnology, USA). Then Protein A/G magnetic beads were added into and incubated with the DNA-protein-antibody complexes. Subsequently, they were washed with gradient salt buffer and eluted with 1% SDS/NaHCO3. Finally, the enriched DNA fragments were subjected to qPCR analysis.

Luciferase assay

Indicated cells were pre-seeded in a 24-well plate. The empty pGL3 luciferase vector, or the pGL3 expressing ACSL5 plasmid was transiently co-transfected into HEK293T cells with the Renilla luciferase plasmid (pRL-TK) as a control. The ratio for the test plasmid: control plasmid was used at 50:1. 48 h later, luciferase activities were measured under instructions of the Dual-Luciferase Reporter Gene Assay Kit (Vazyme, Nanjing, China, DL101-01). Firefly luciferase activities were described by the average of triplicates.

Xenograft tumorigenesis in vivo. RH30 cells ($5*10^6$ cells per mouse) were inoculated into the right flank of the female nude mice aged at 3-4 weeks. When the volume of the tumor reached about 100 mm^3 (the tumor volume was calculated: length*width*width/2), the mice were randomly grouped for treatment. Tumor size and body weight of each mouse were recorded every 2 days. After the mice were euthanized, the transplanted tumors were completely isolated and immediately weighed. The animal experiment was approved by the Animal Ethics Committee of Sun Yat-sen University (SYSU-IACUC-2022-001988).
