# Supplementary material for: AURKB inhibition induces rhabdomyosarcoma apoptosis and ferroptosis through NPM1/SP1/ACSL5 axis
Source: JCI Insight. 2025 Feb 10;10(3):e182429. doi: 10.1172/jci.insight.182429 (PMC11948576; doi:10.1172/jci.insight.182429)

Full unedited blots for Figure 1F

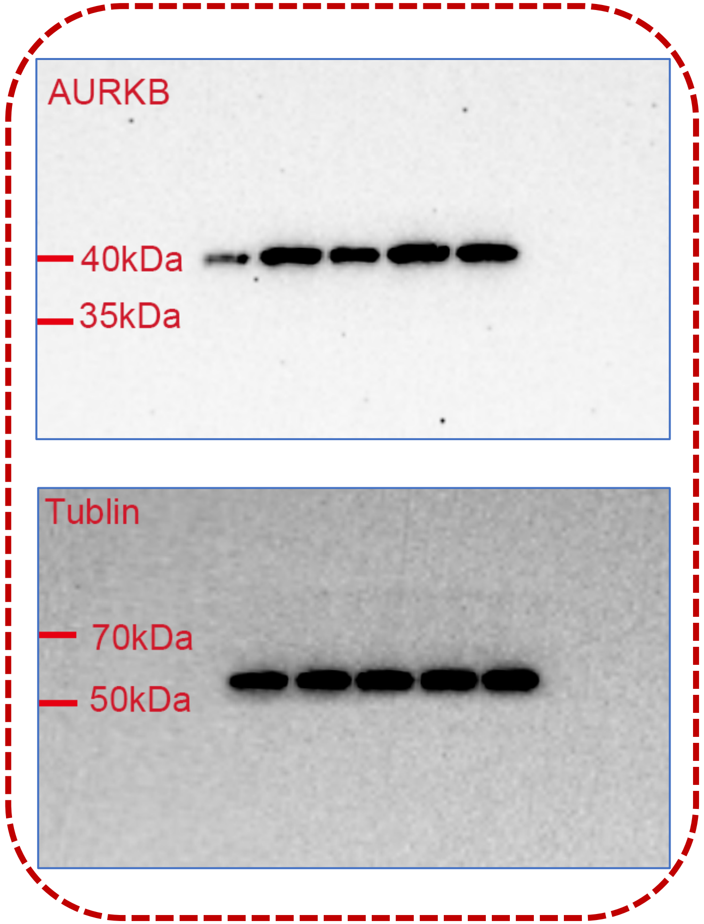

Full unedited blots for Figure 3I-RD

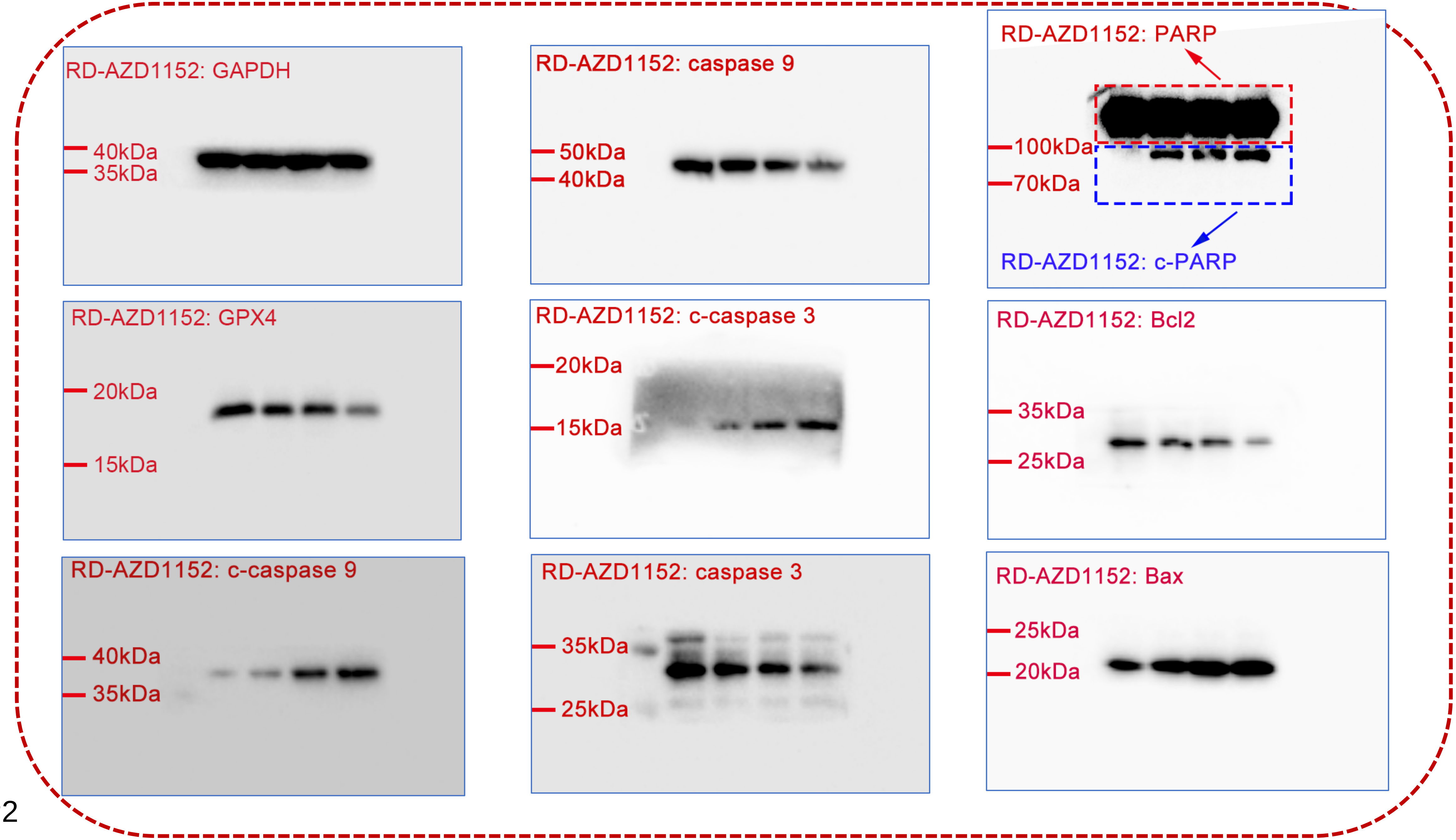

Full unedited blots for Figure 3I-RH30

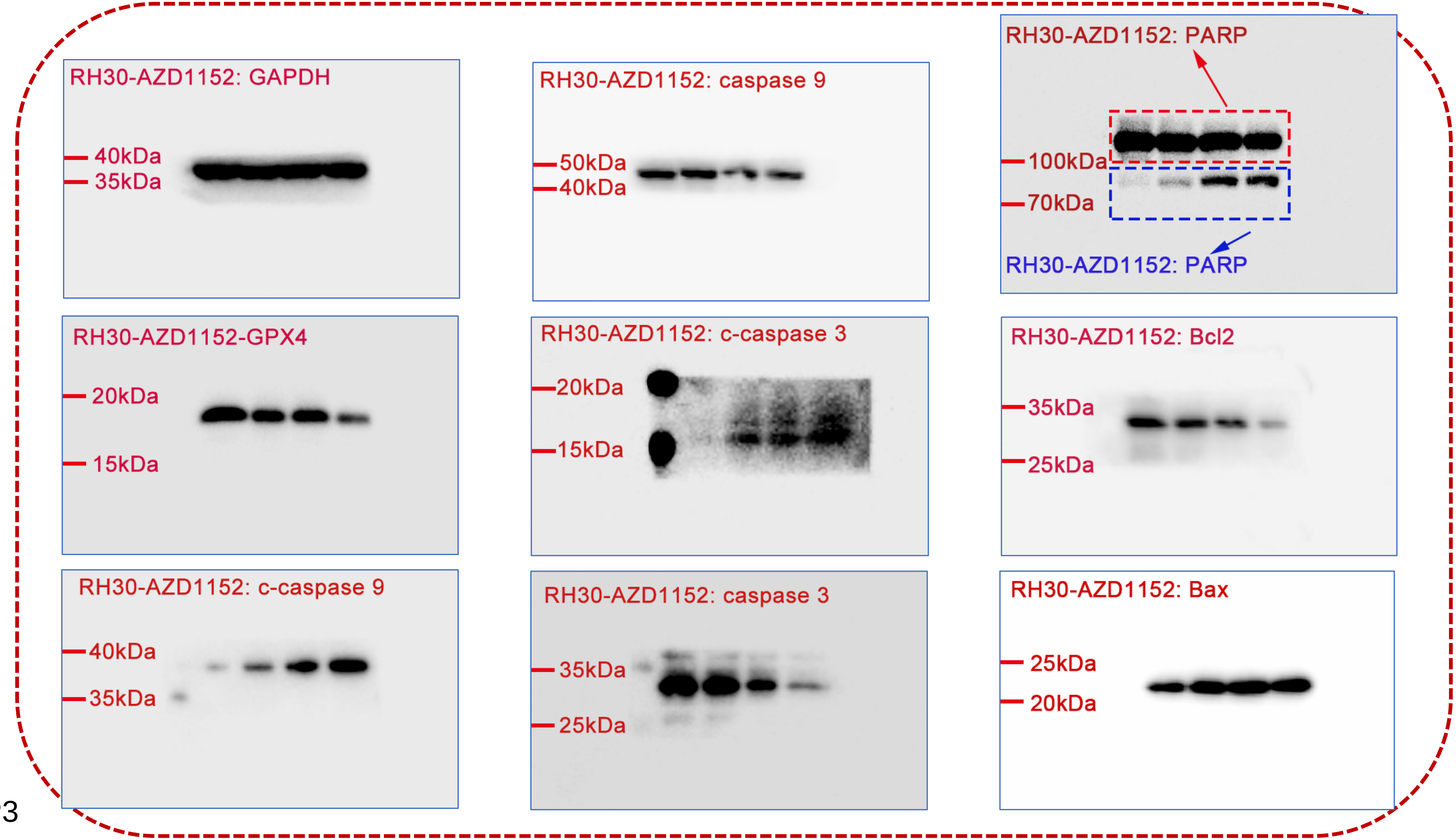

Full unedited blots for Figure 3J-RD

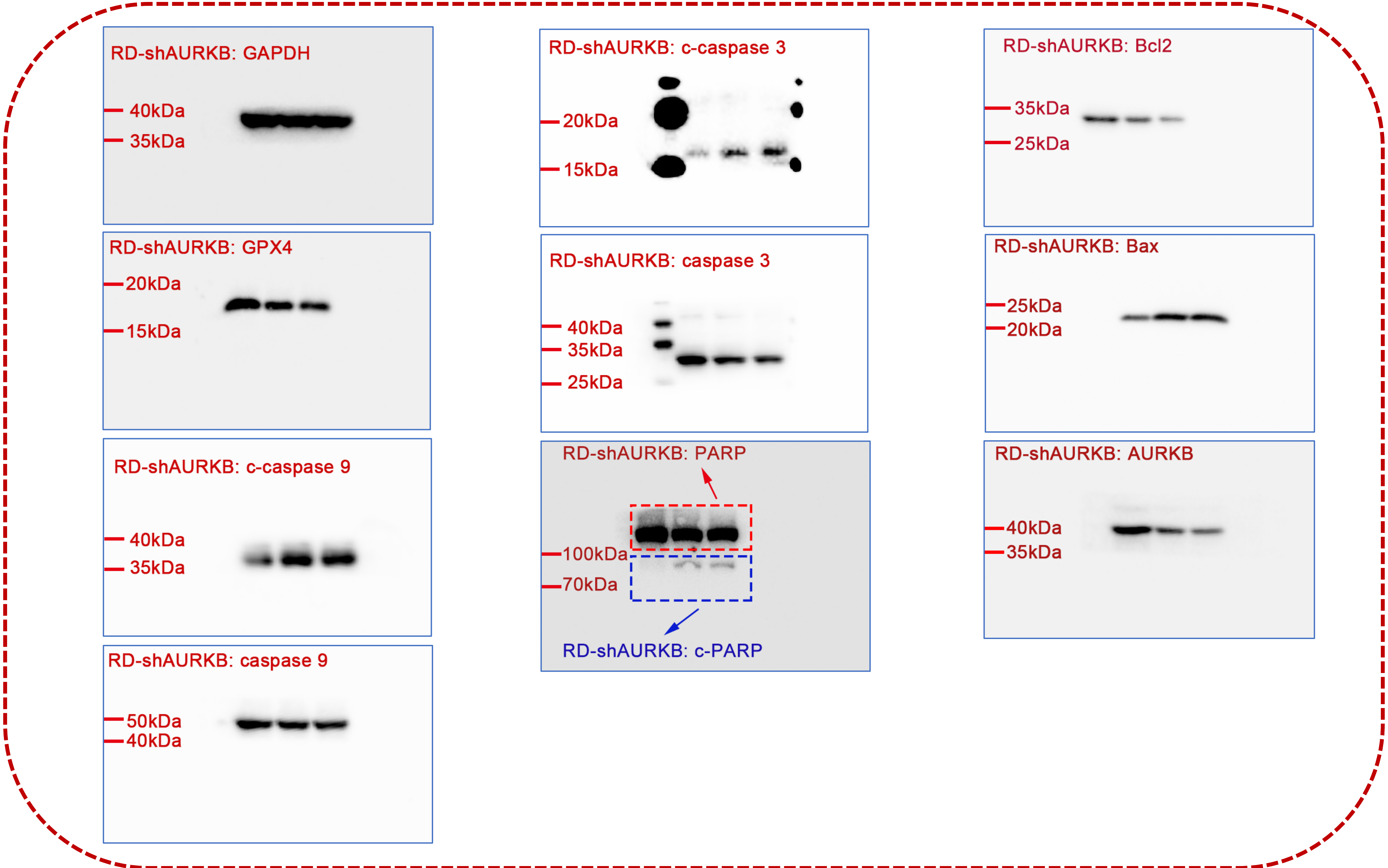

P4

Full unedited blots for Figure 3J-RH30

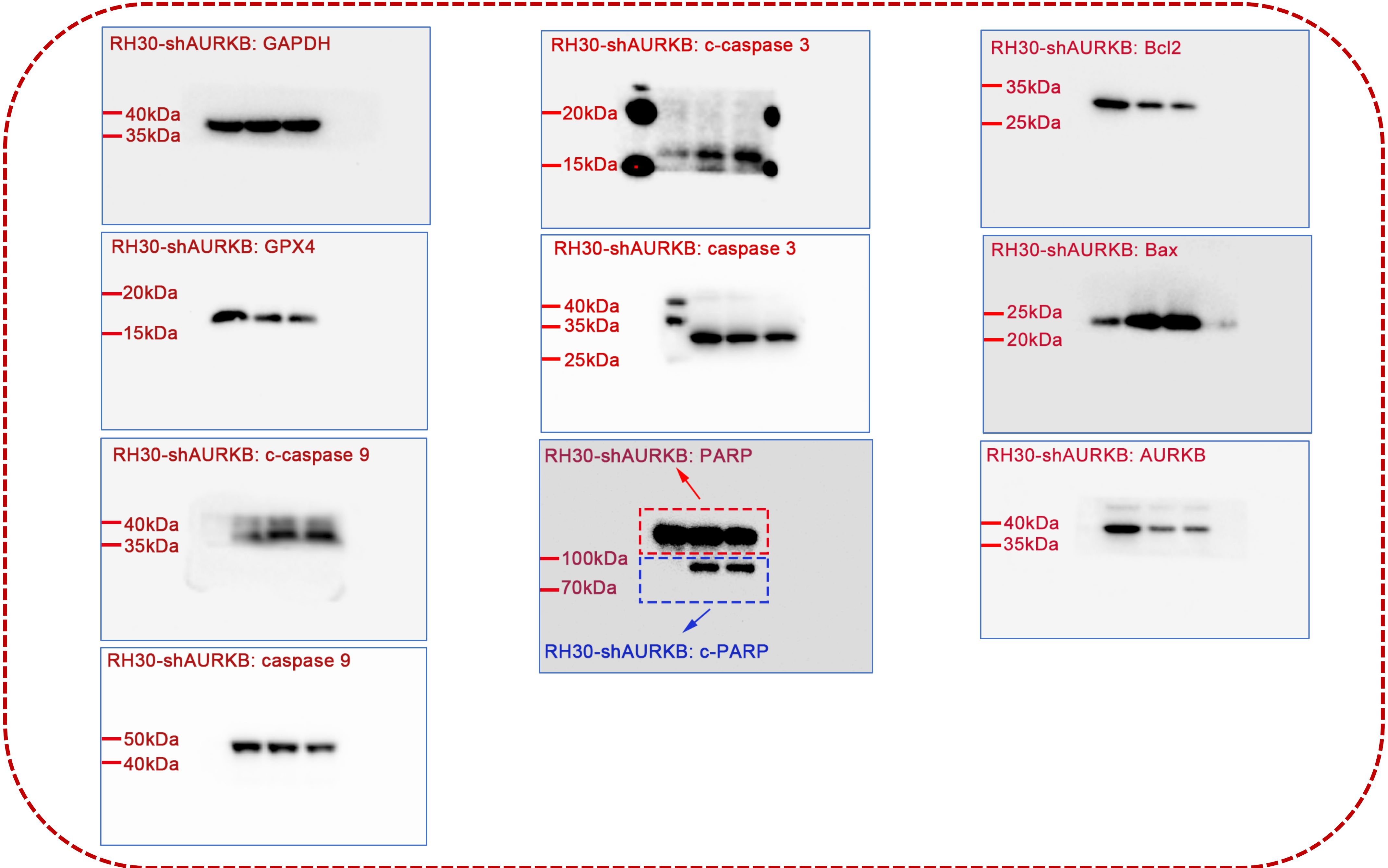

Full unedited blots for Figure 5C

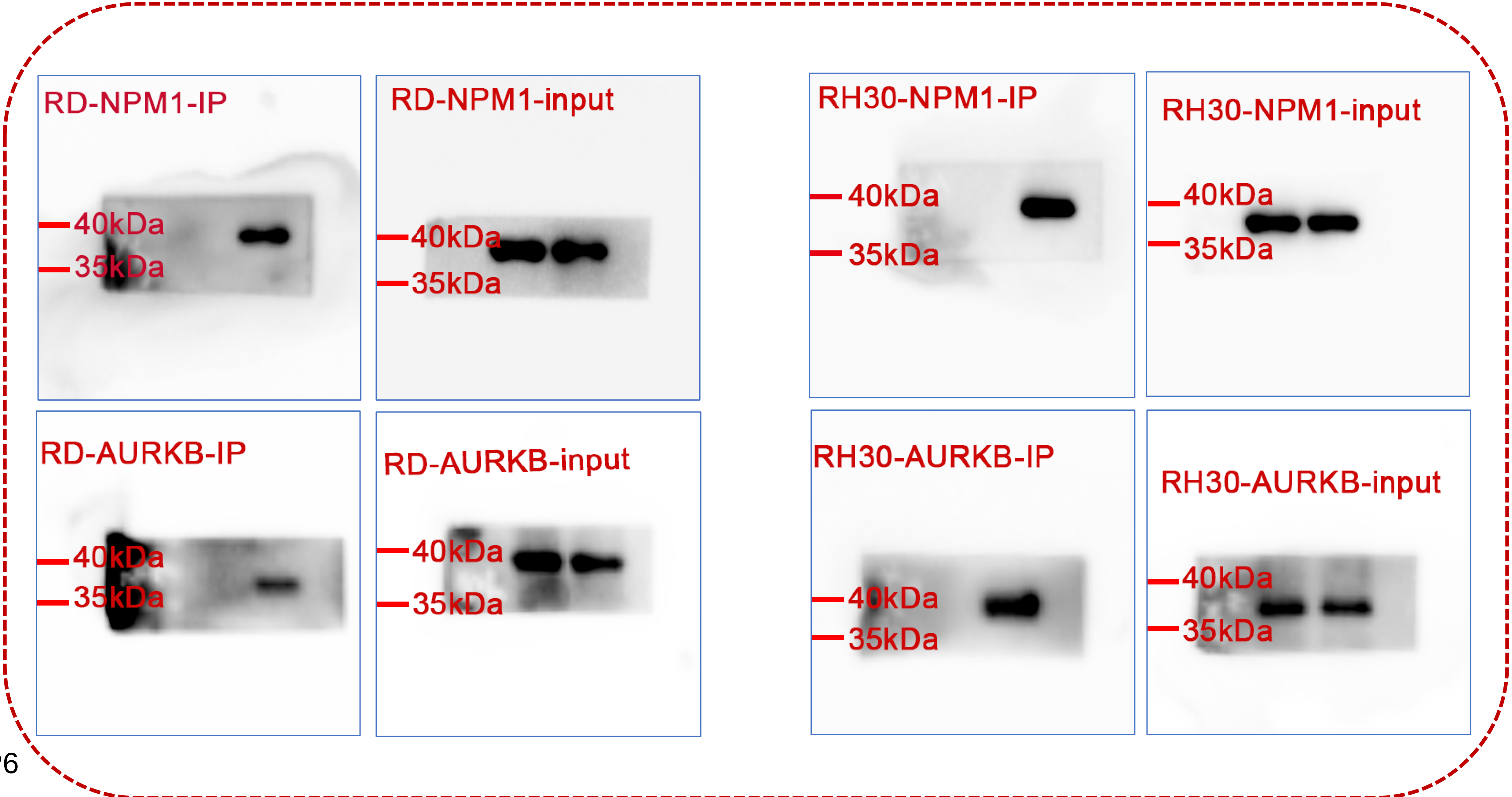

Full unedited blots for Figure 5D：RD

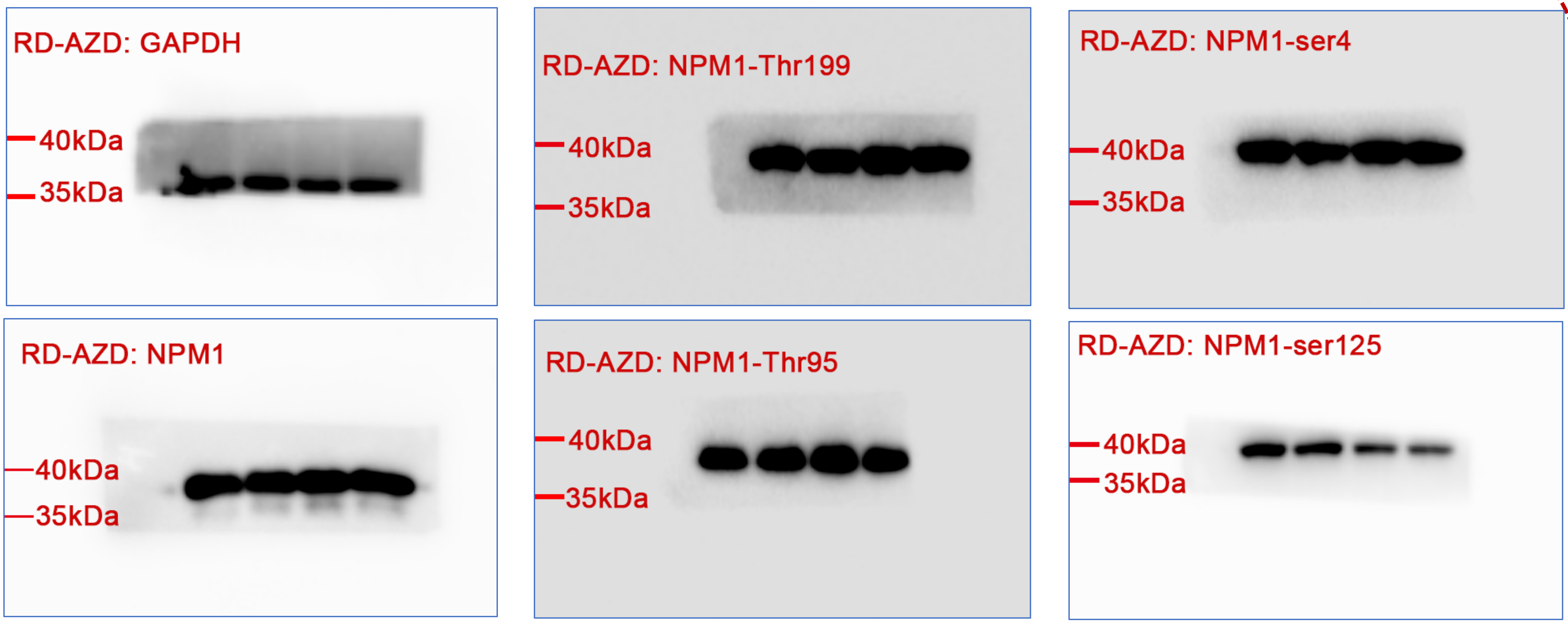

Full unedited blots for Figure 5D: RH30

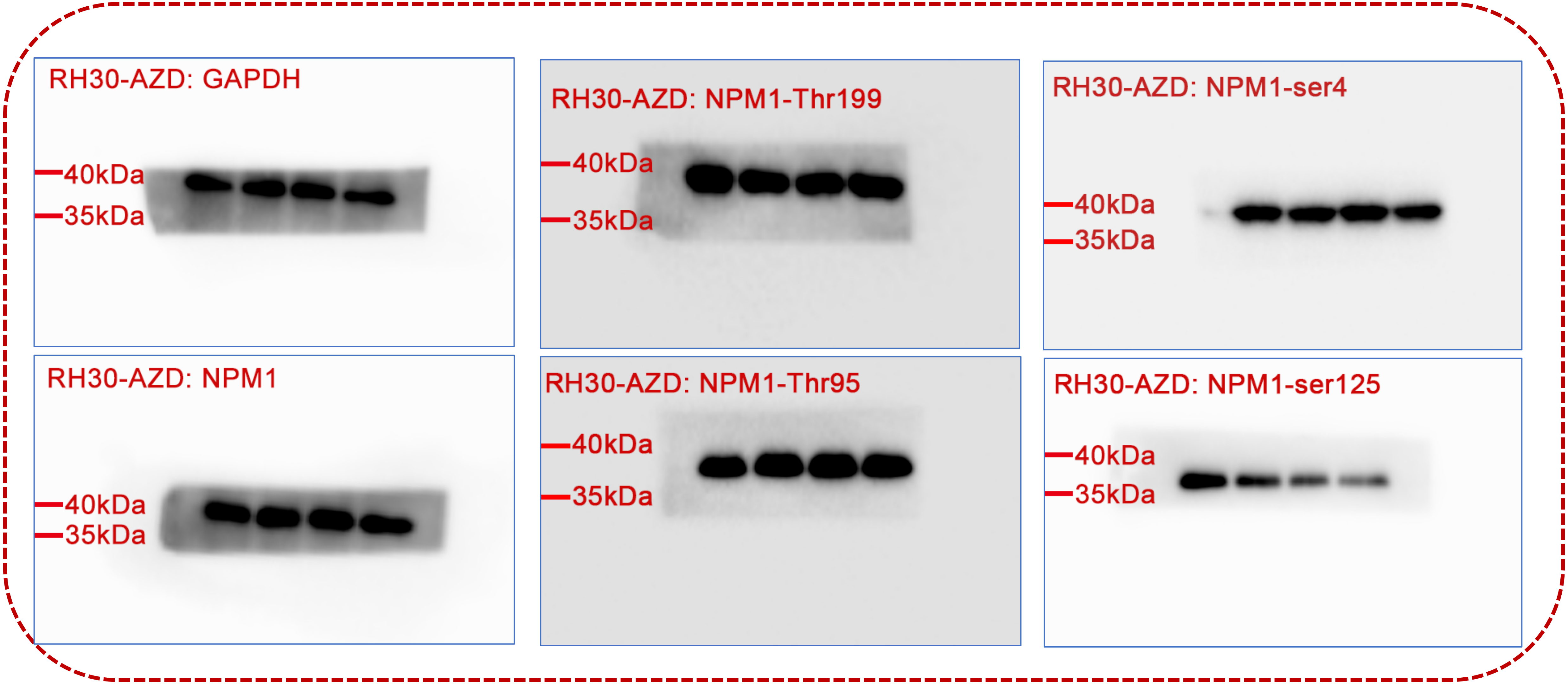

Full unedited blots for Figure 5E

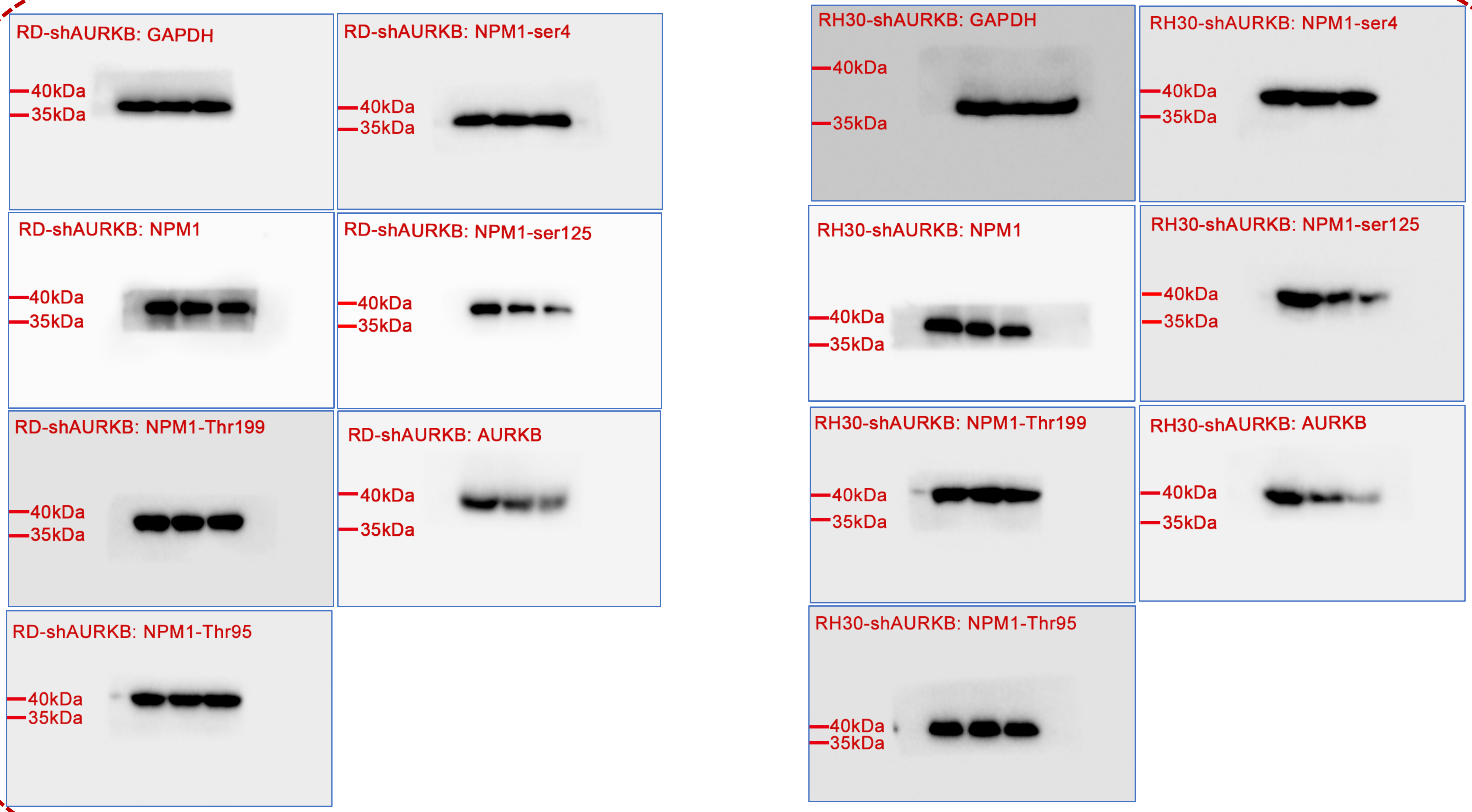

Full unedited blots for Figure 5M: RD

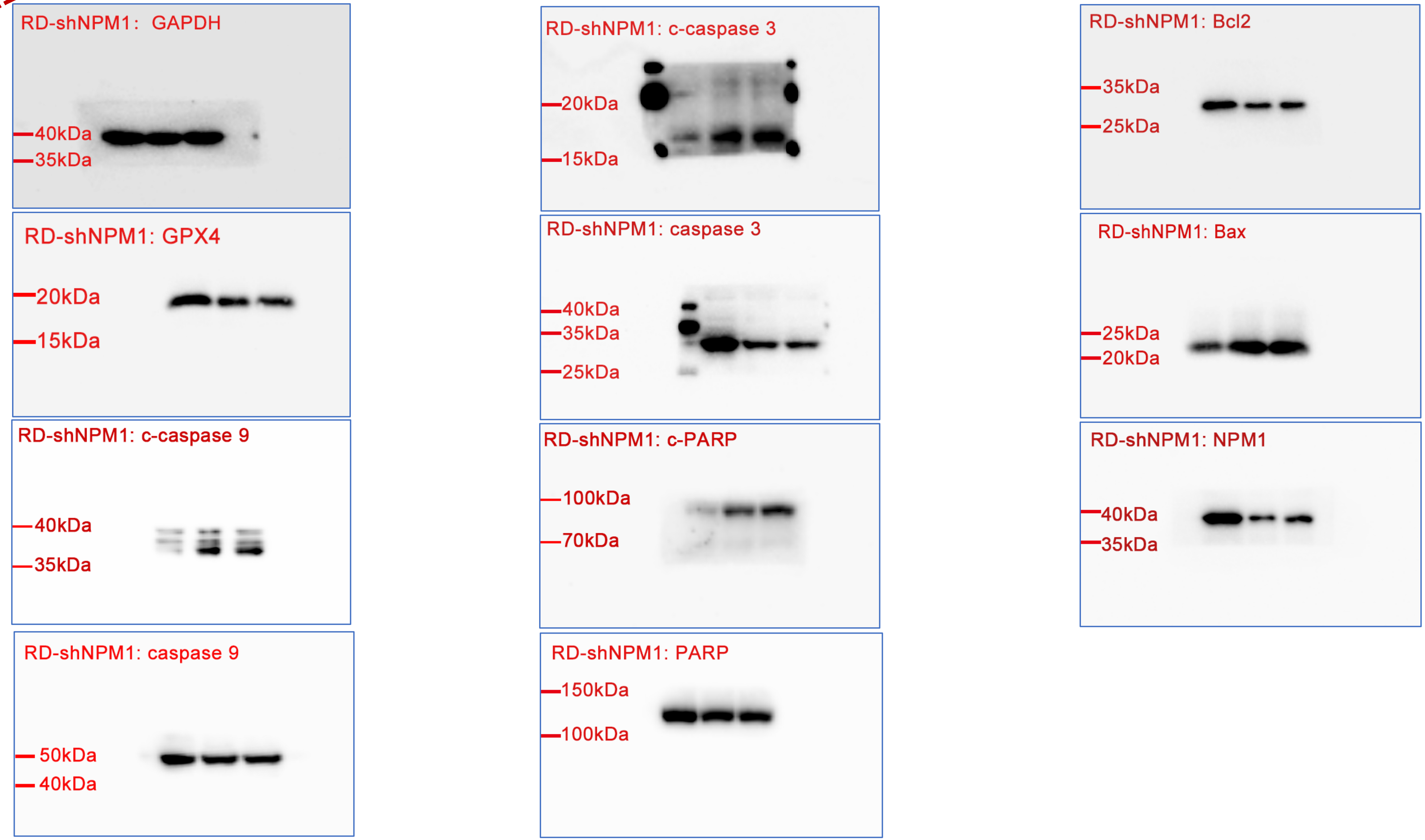

Full unedited blots for Figure 5M: RH30

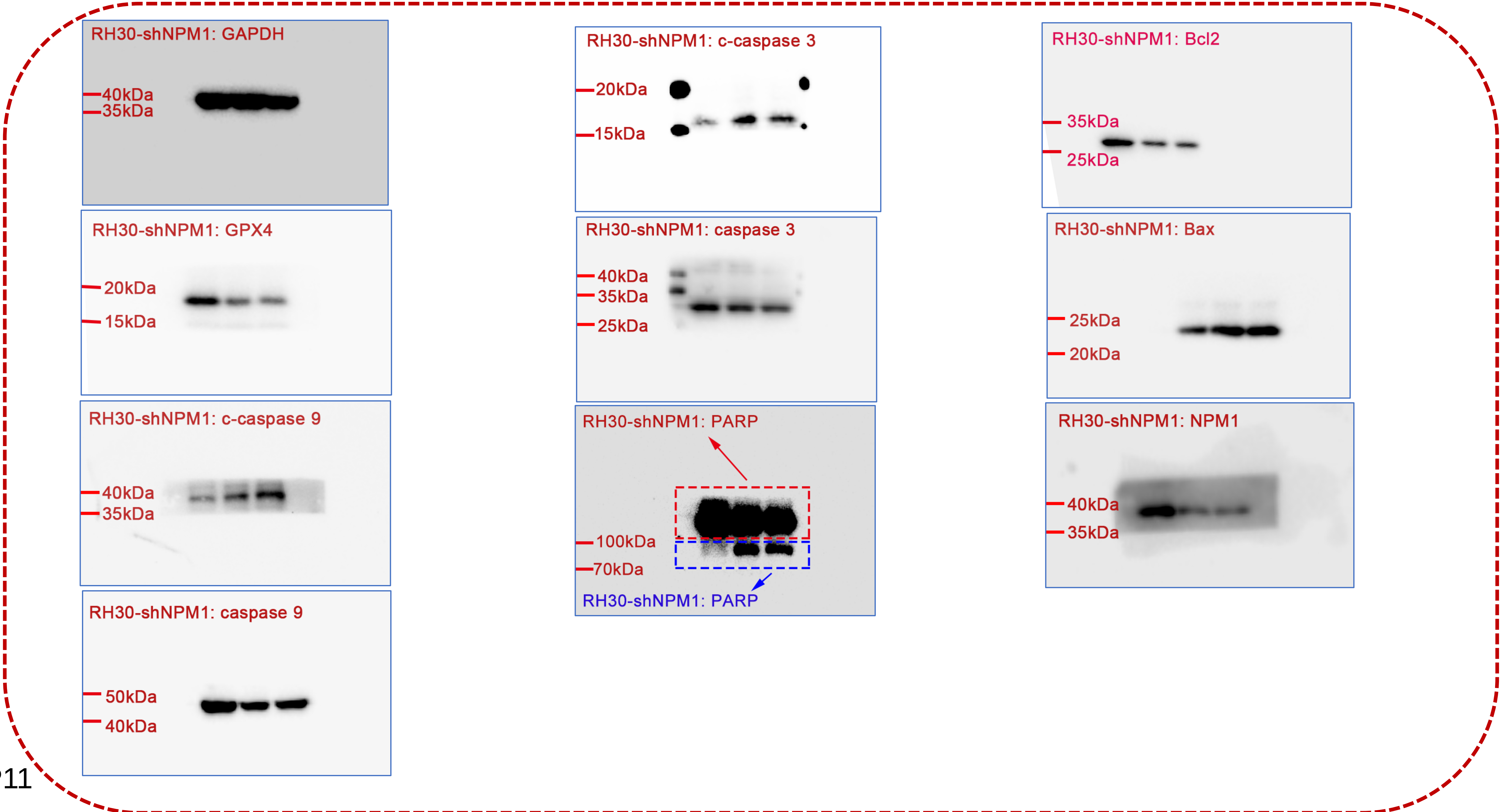

Full unedited blots for Figure 6F

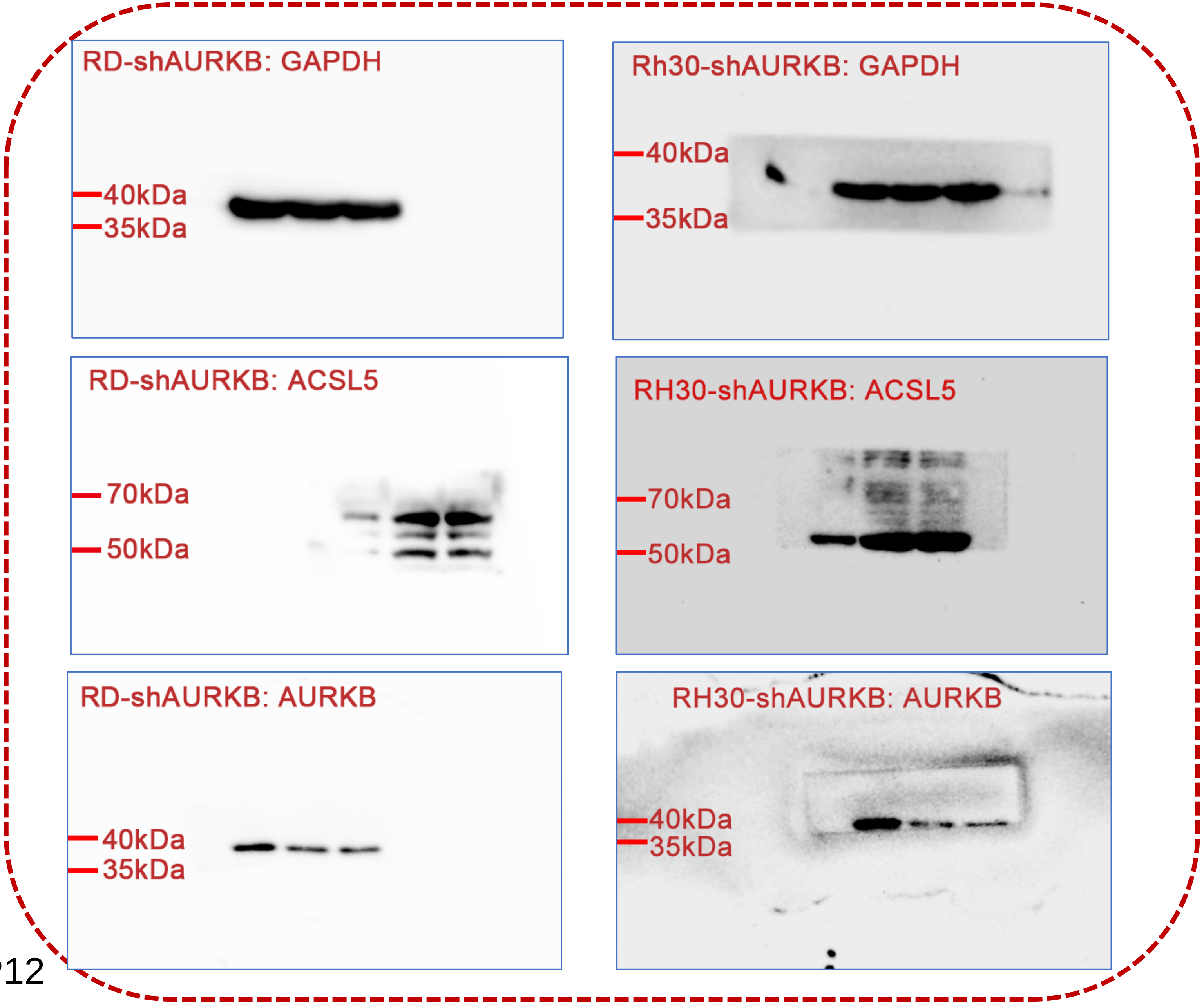

Full unedited gel for Figure 6G

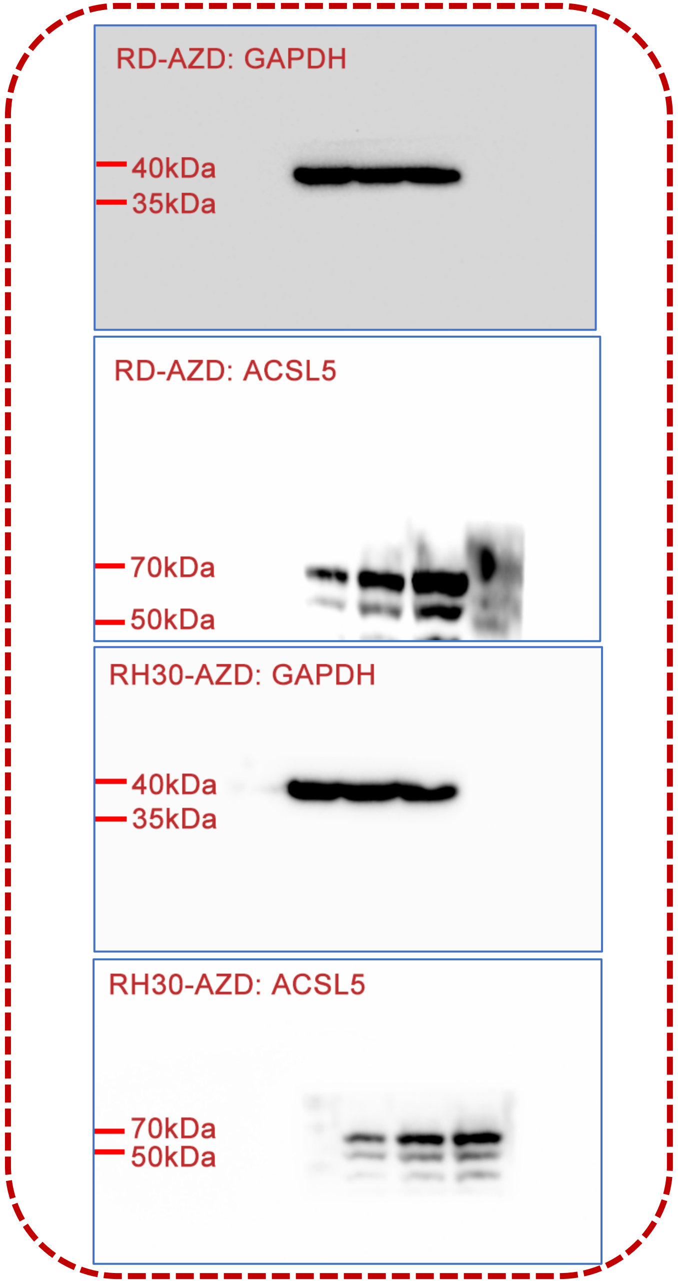

Full unedited blots for Figure 6M

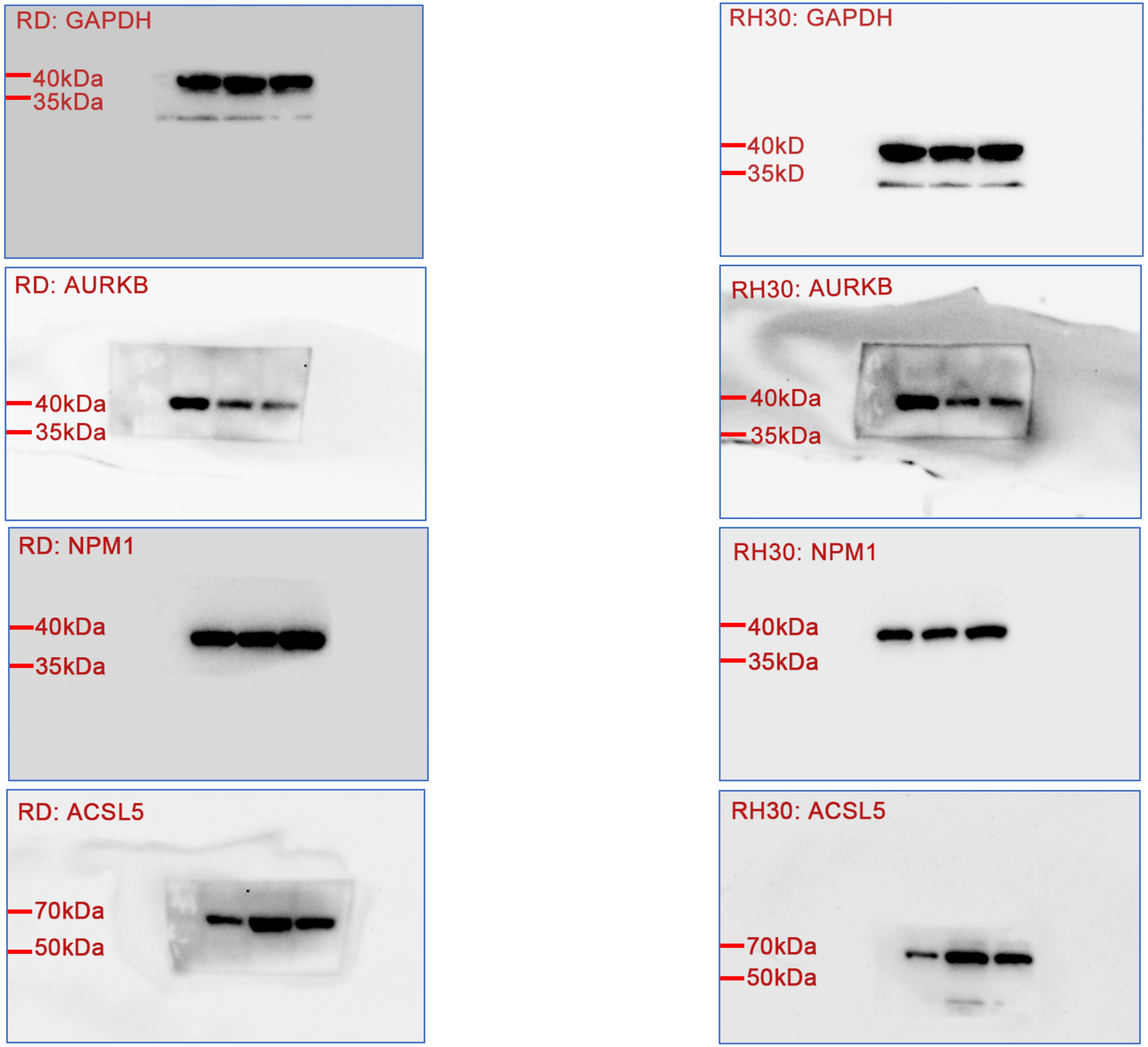

Full unedited blots for Figure 7B

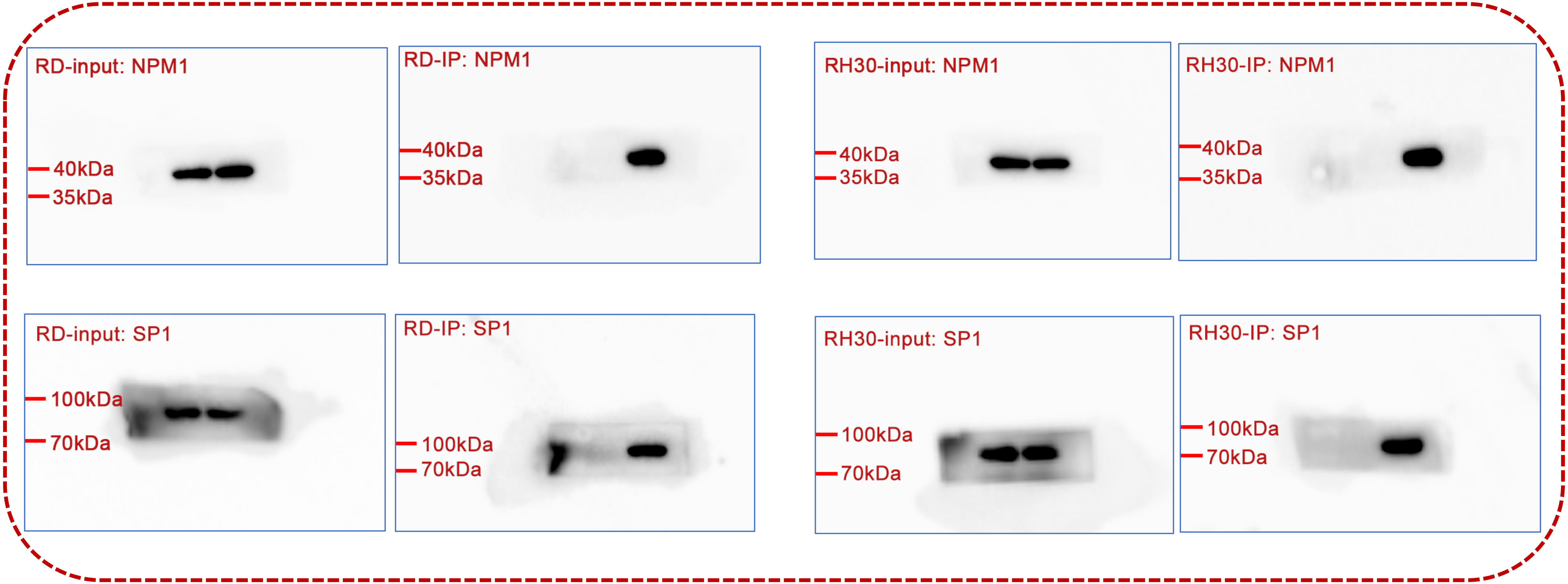

Full unedited blots for Figure 7D

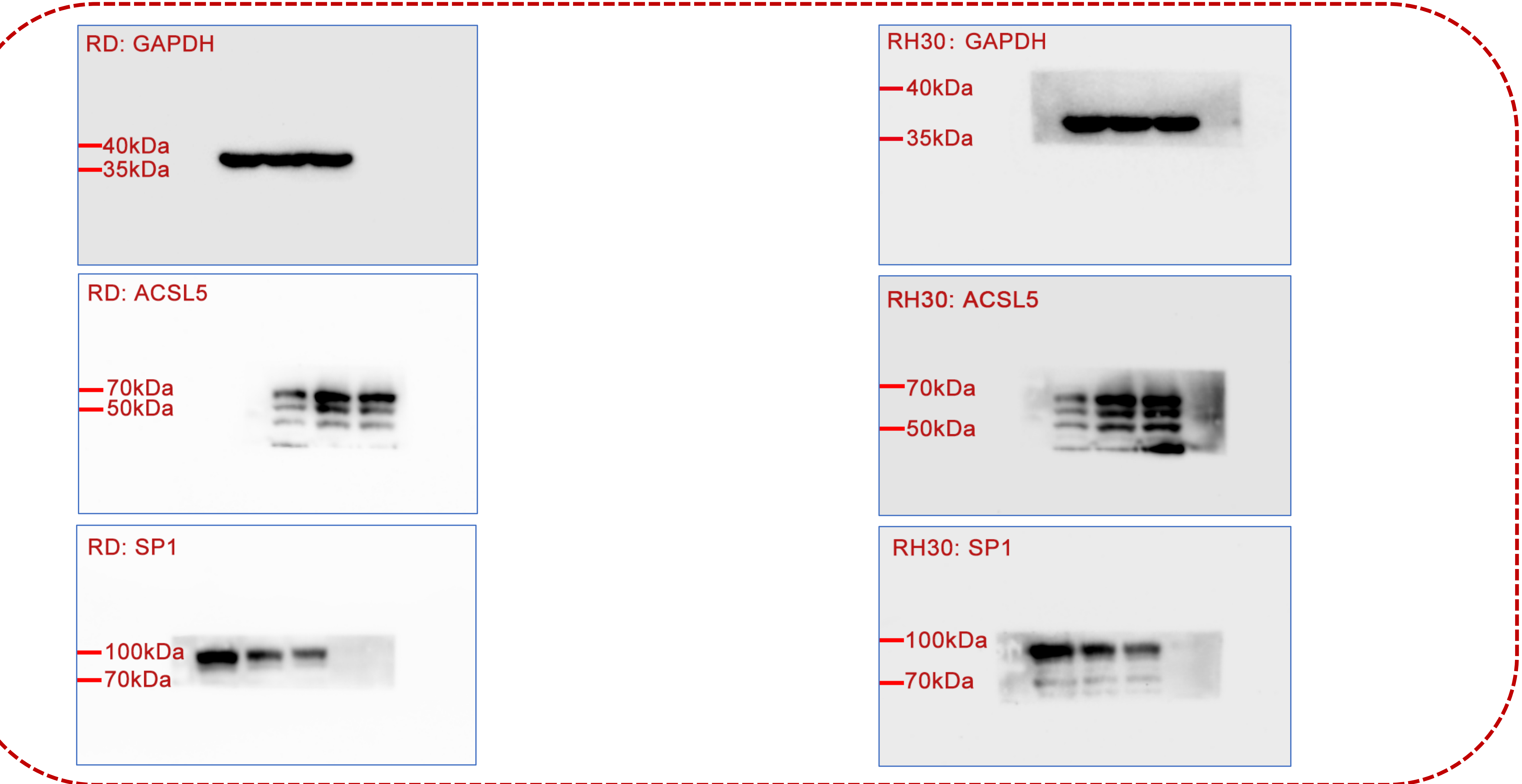

Full unedited blots for Figure 7L：RD

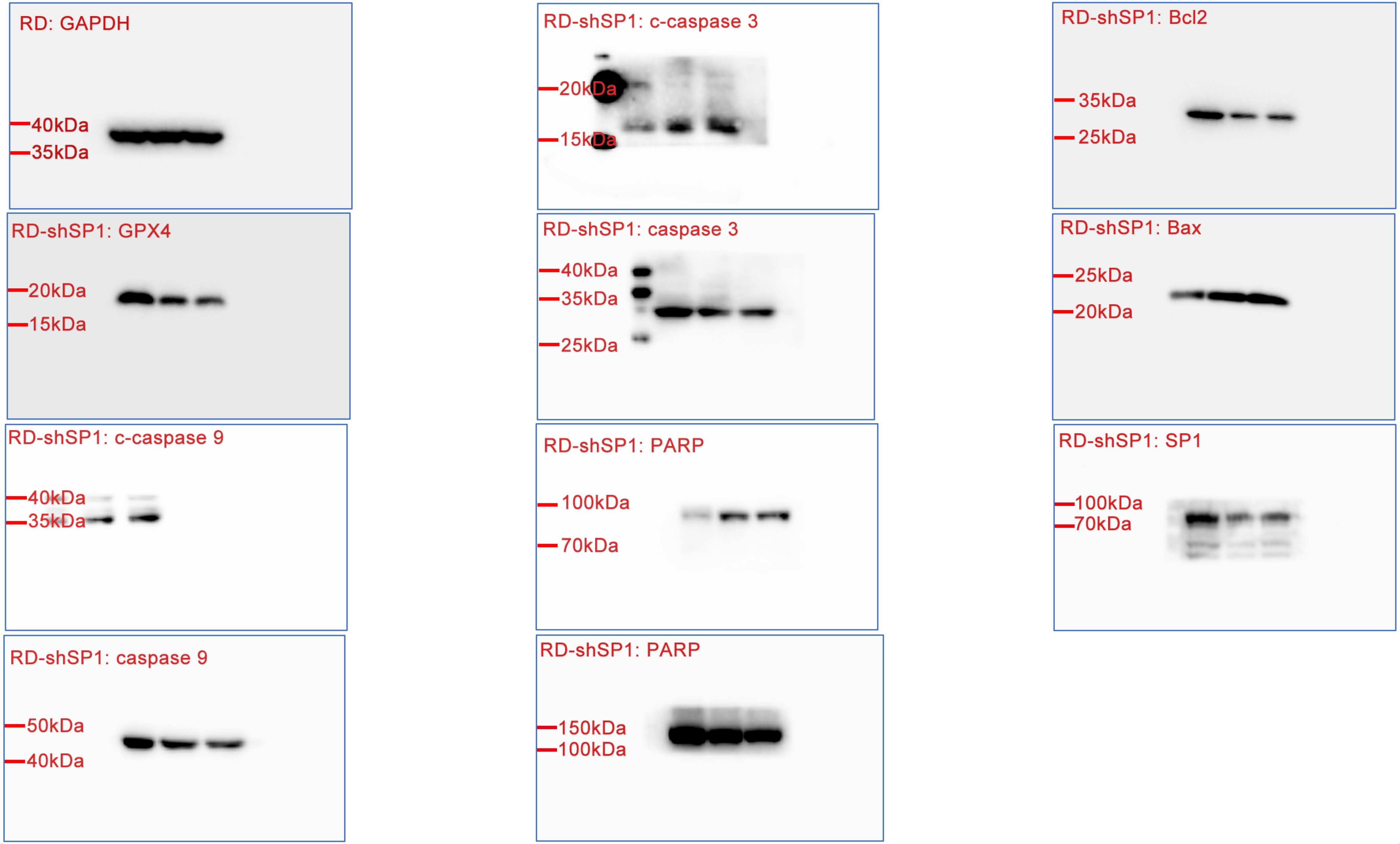

Full unedited blots for Figure 7L: RH30

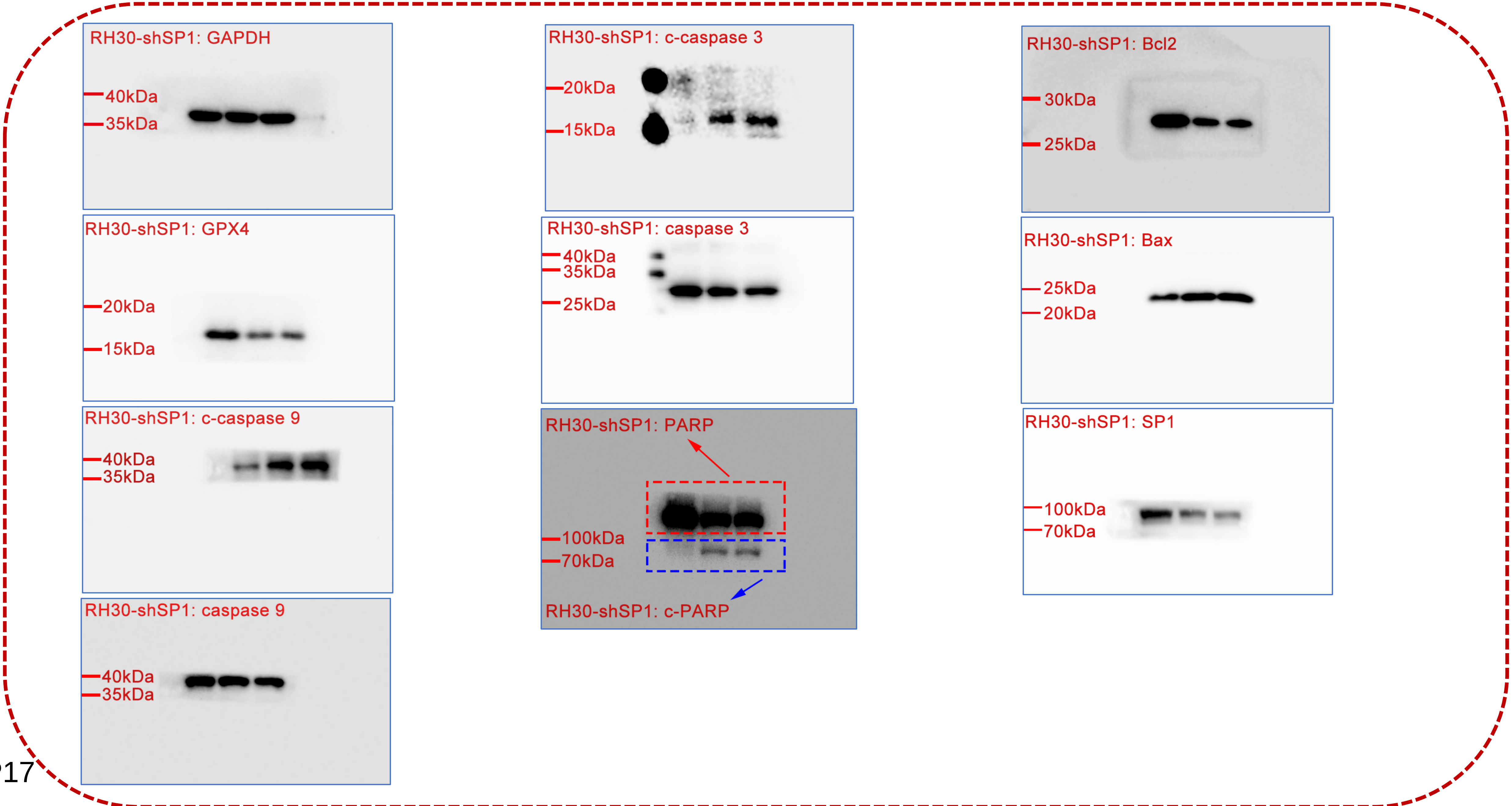

Full unedited blots for Figure 7M

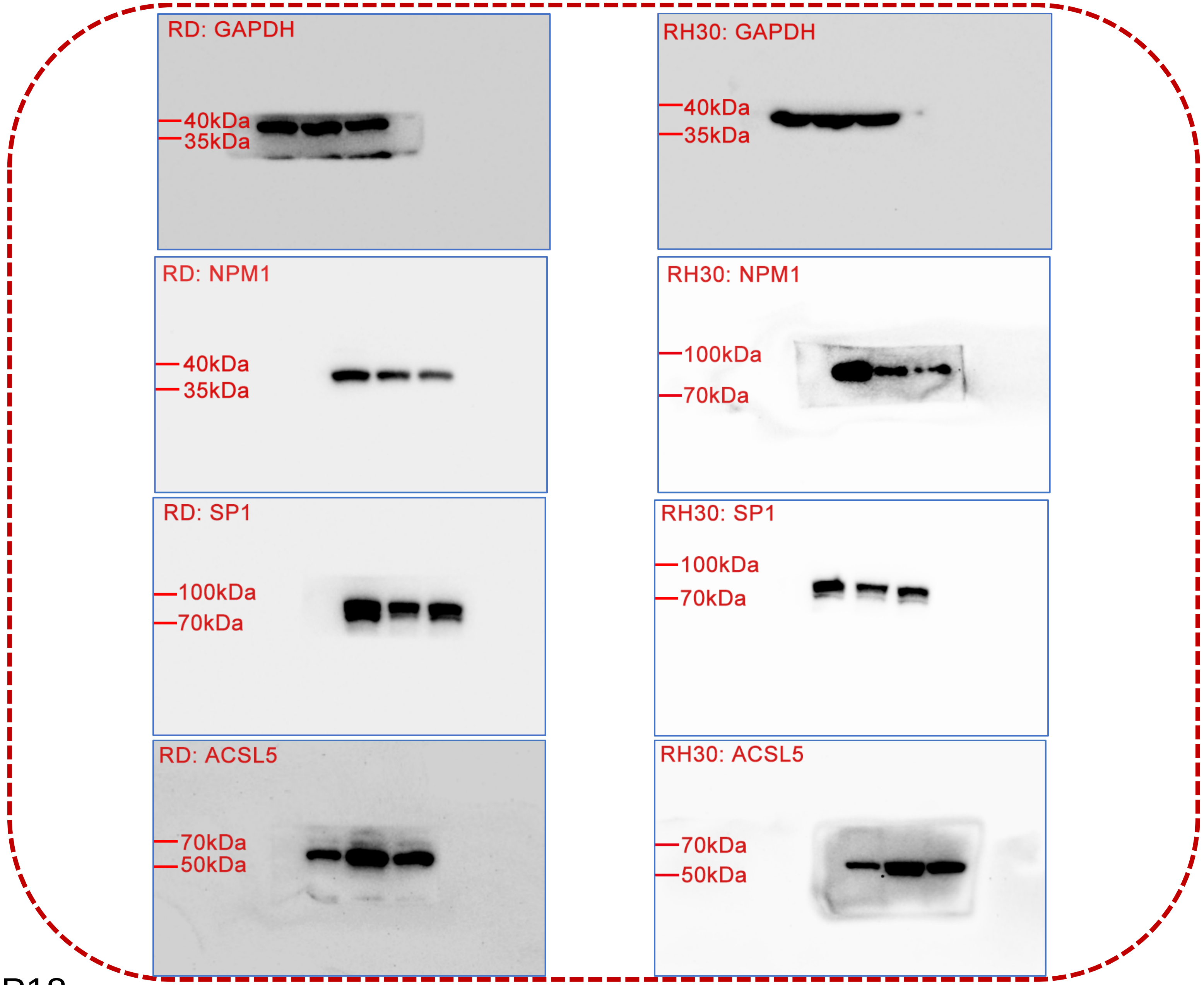

Full unedited blots for Figure 7N

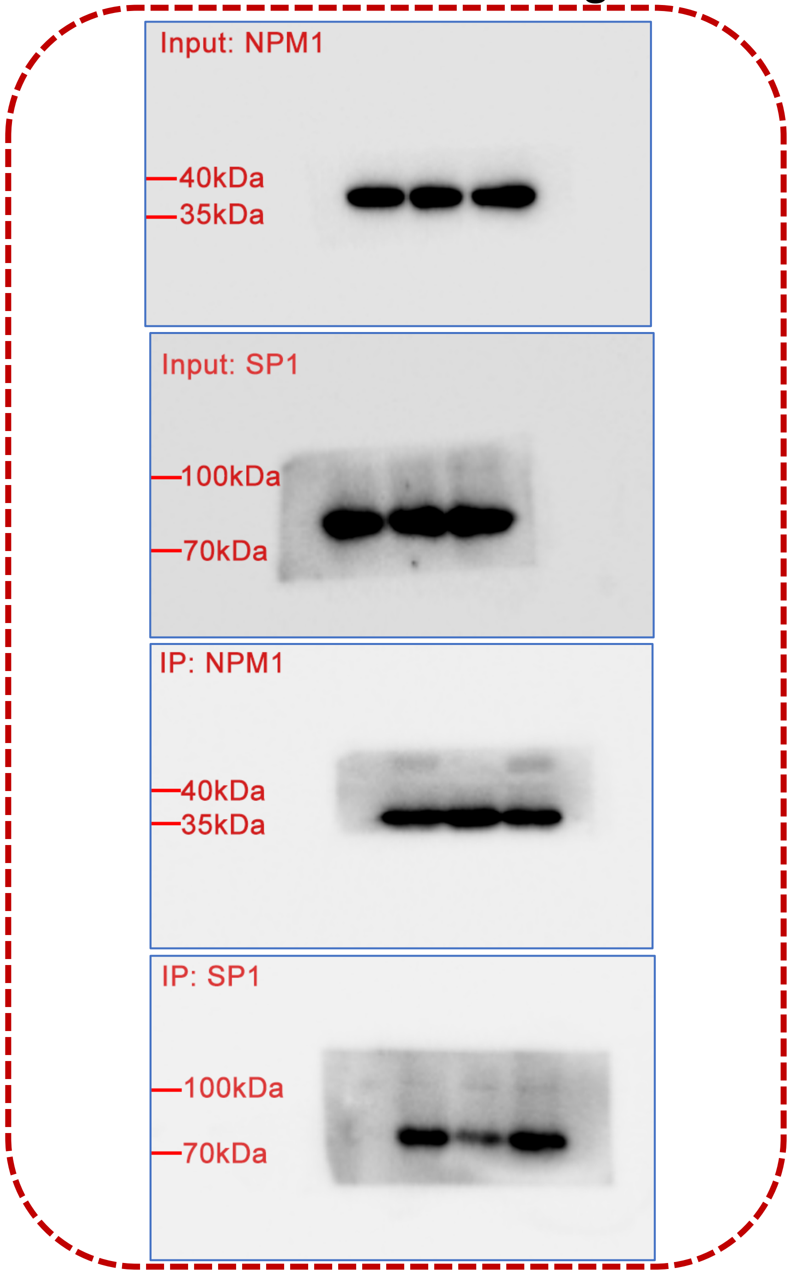

Full unedited blots for Figure 7O

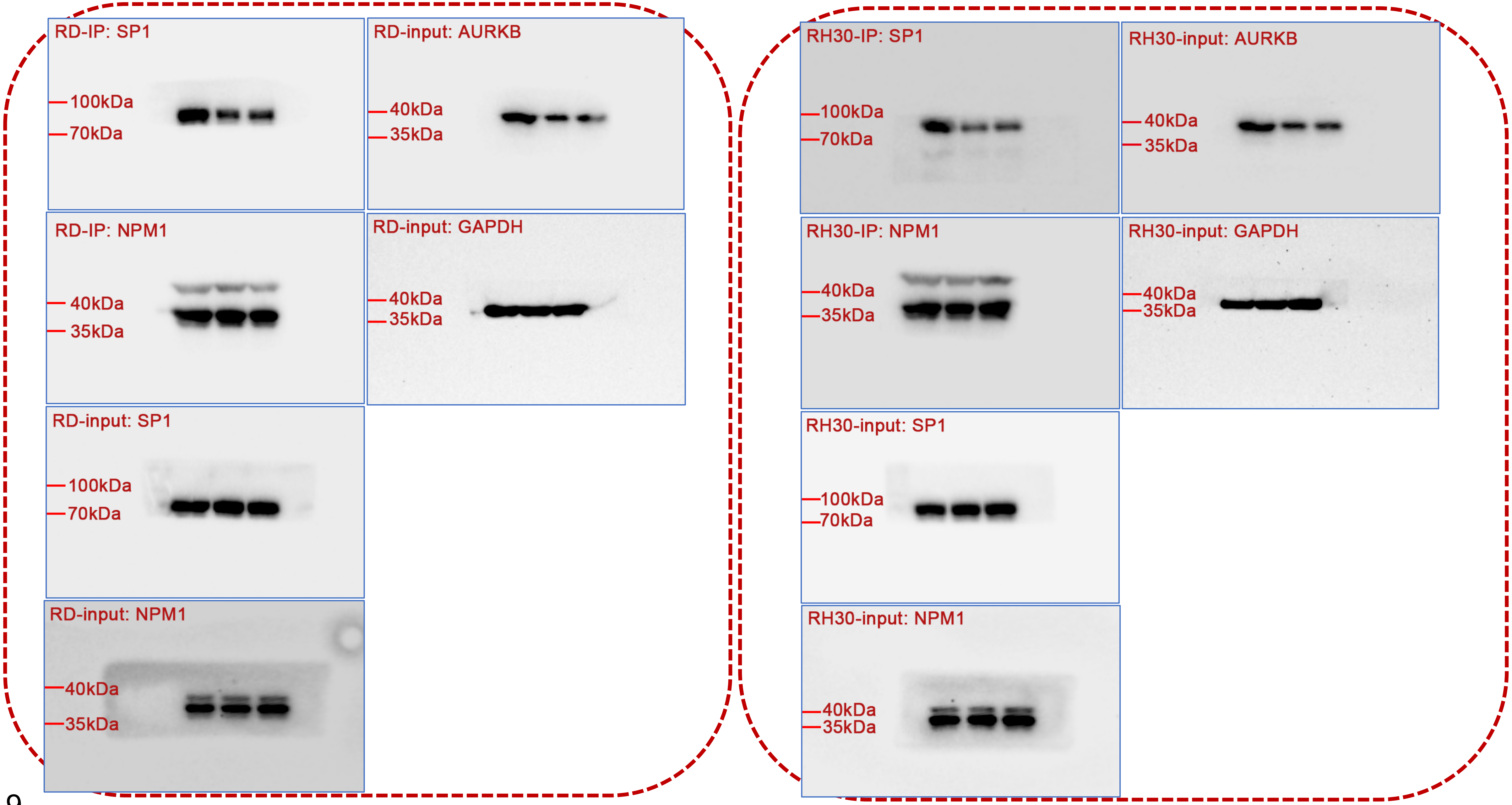

Full unedited blots for Figure 8C

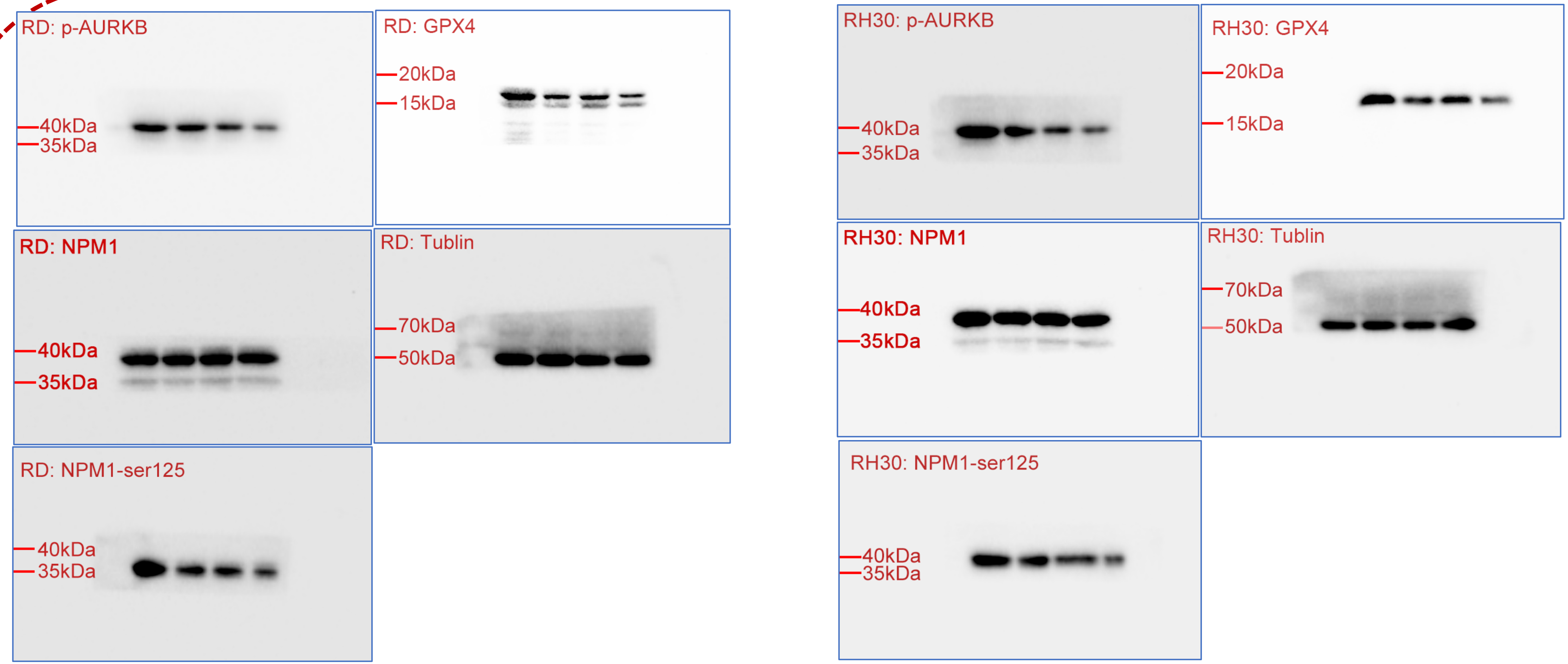

Full unedited blots for Supplementary Figure 5A

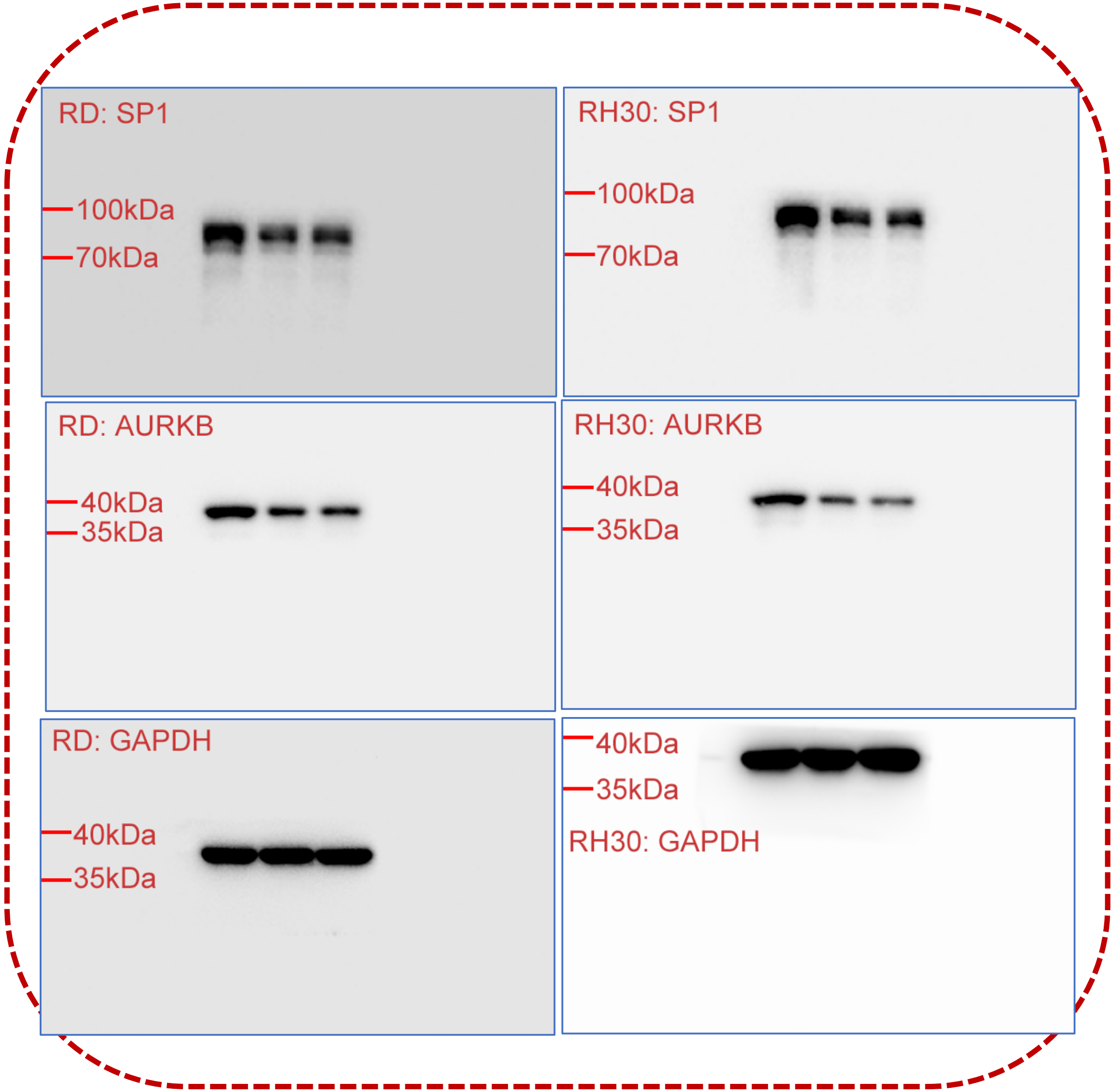

Full unedited blots for Supplementary Figure 5B

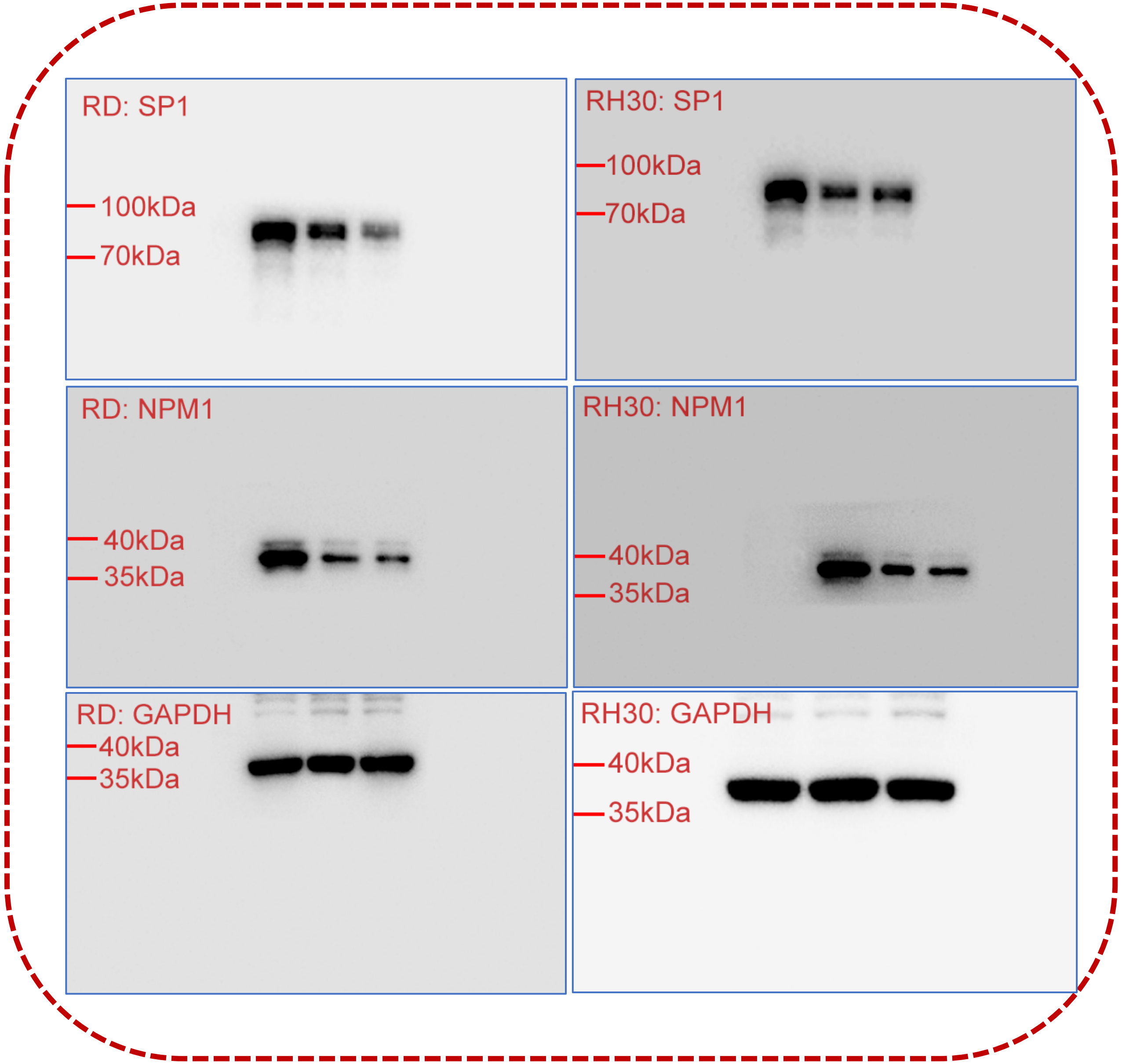

Full unedited blots for Supplementary Figure 5E

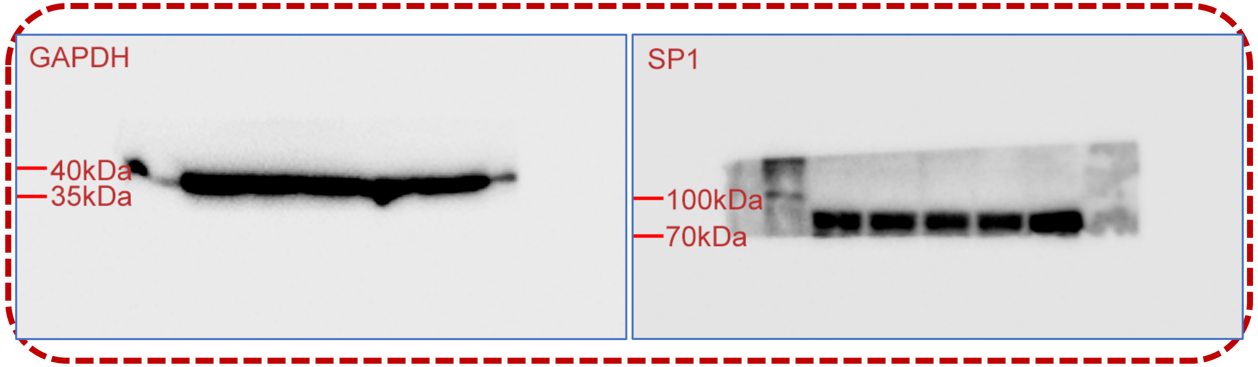

Full unedited blots for Supplementary Figure 5F

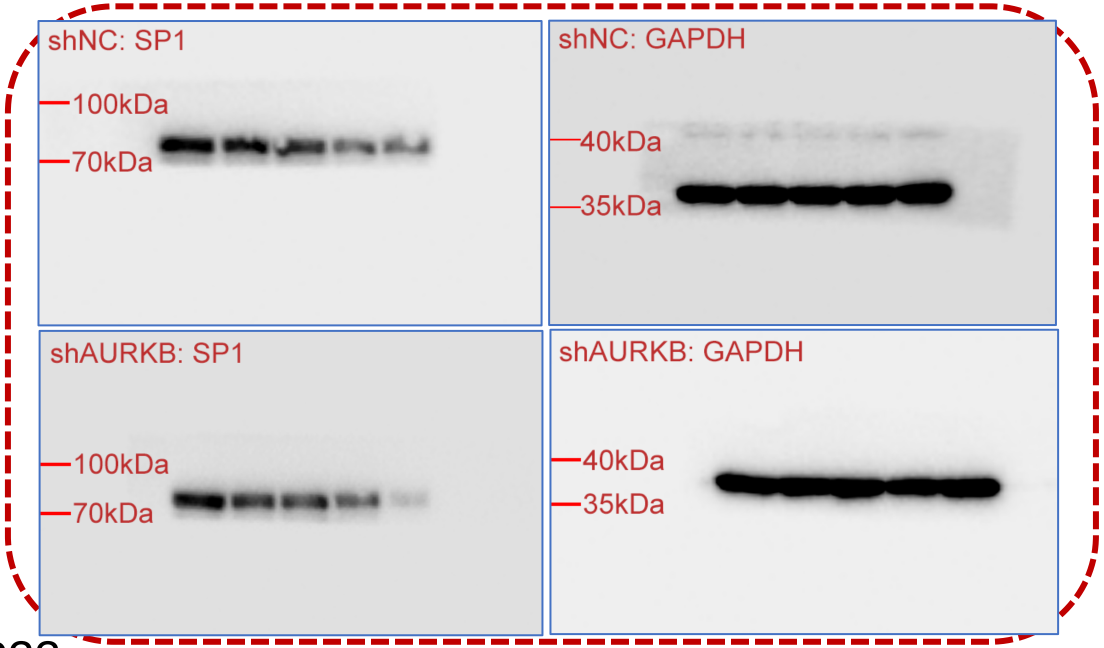

Full unedited blots for Supplementary Figure 5G

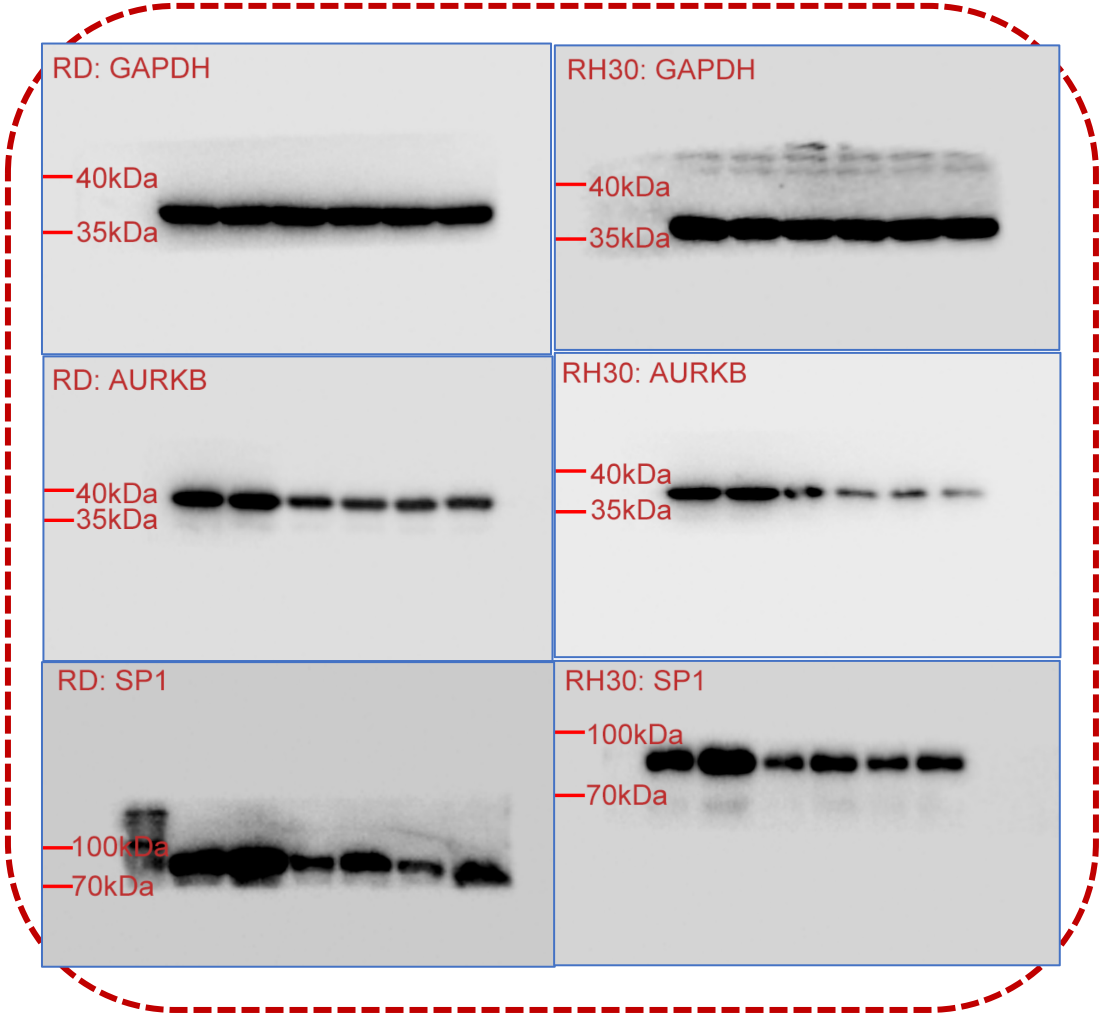

Full unedited blots for Supplementary Figure 5H

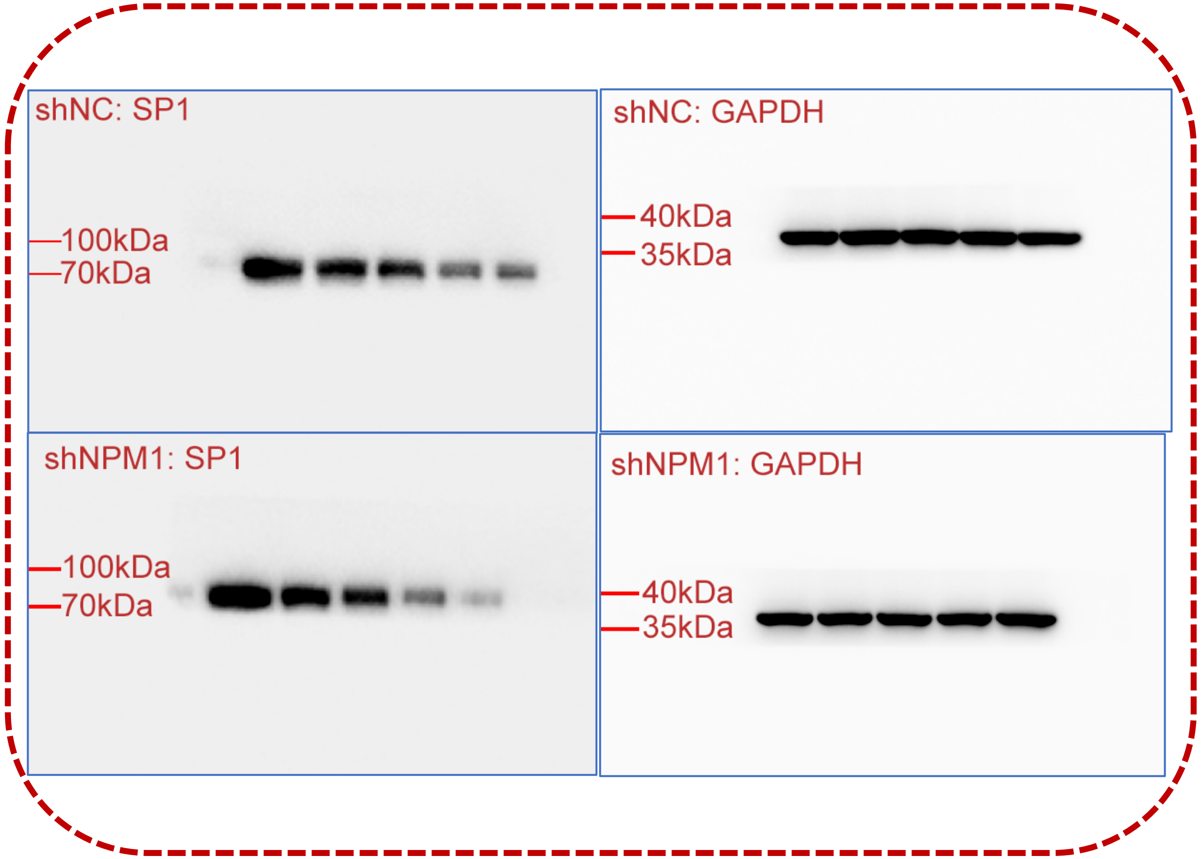

Full unedited blots for Supplementary Figure 5I

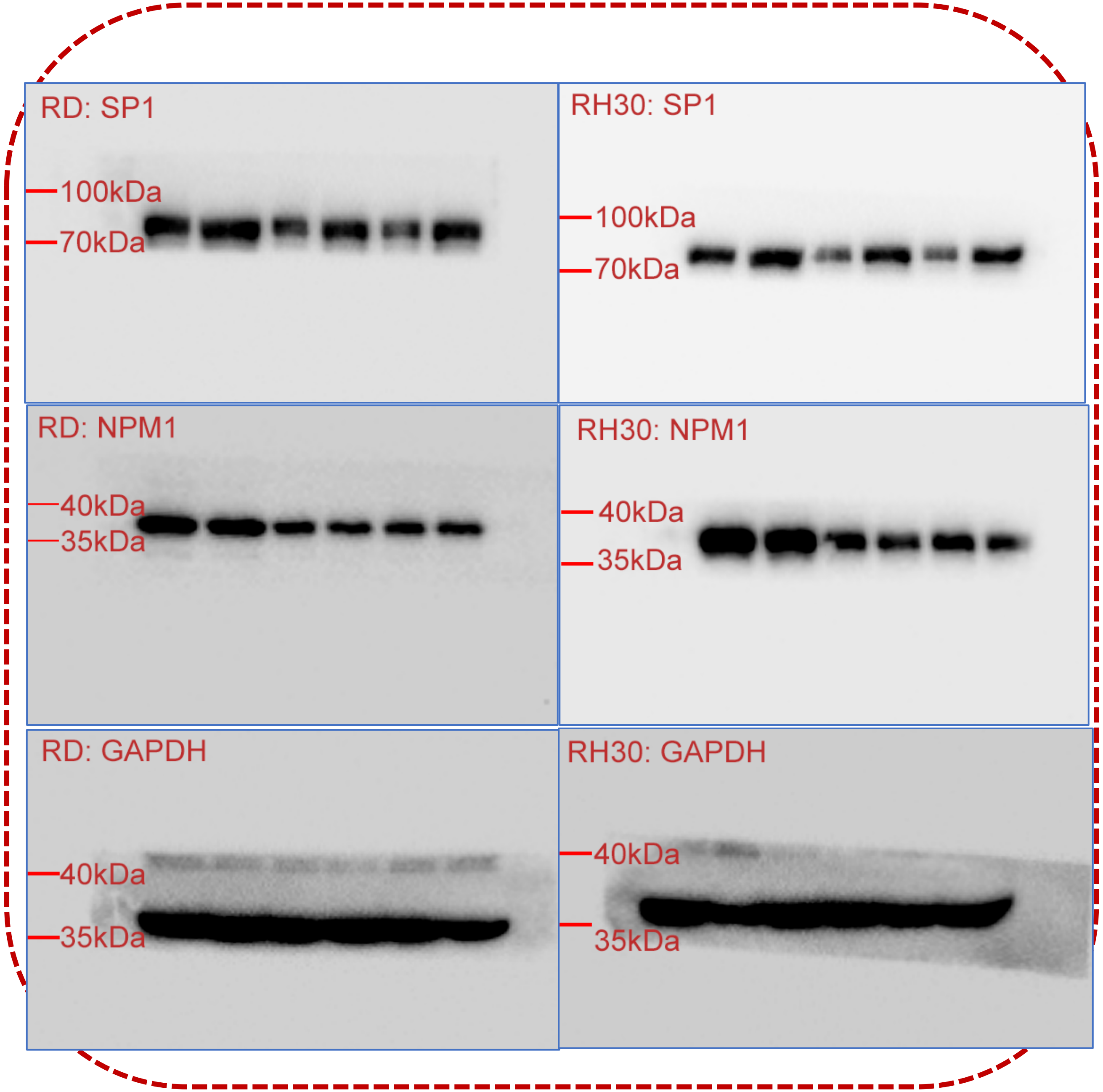

Full unedited blots for Supplementary Figure 5J

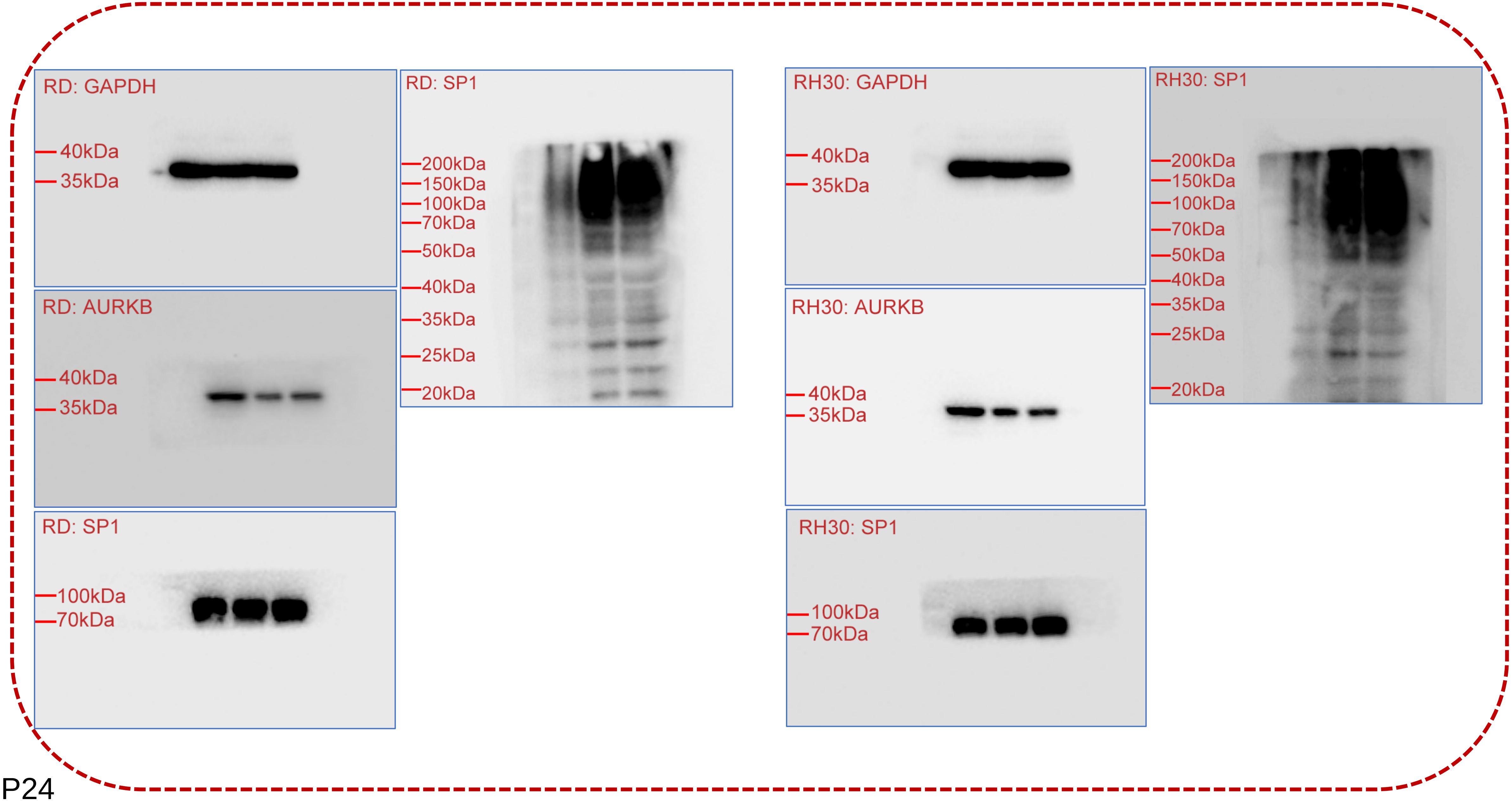

Full unedited blots for Supplementary Figure 5K

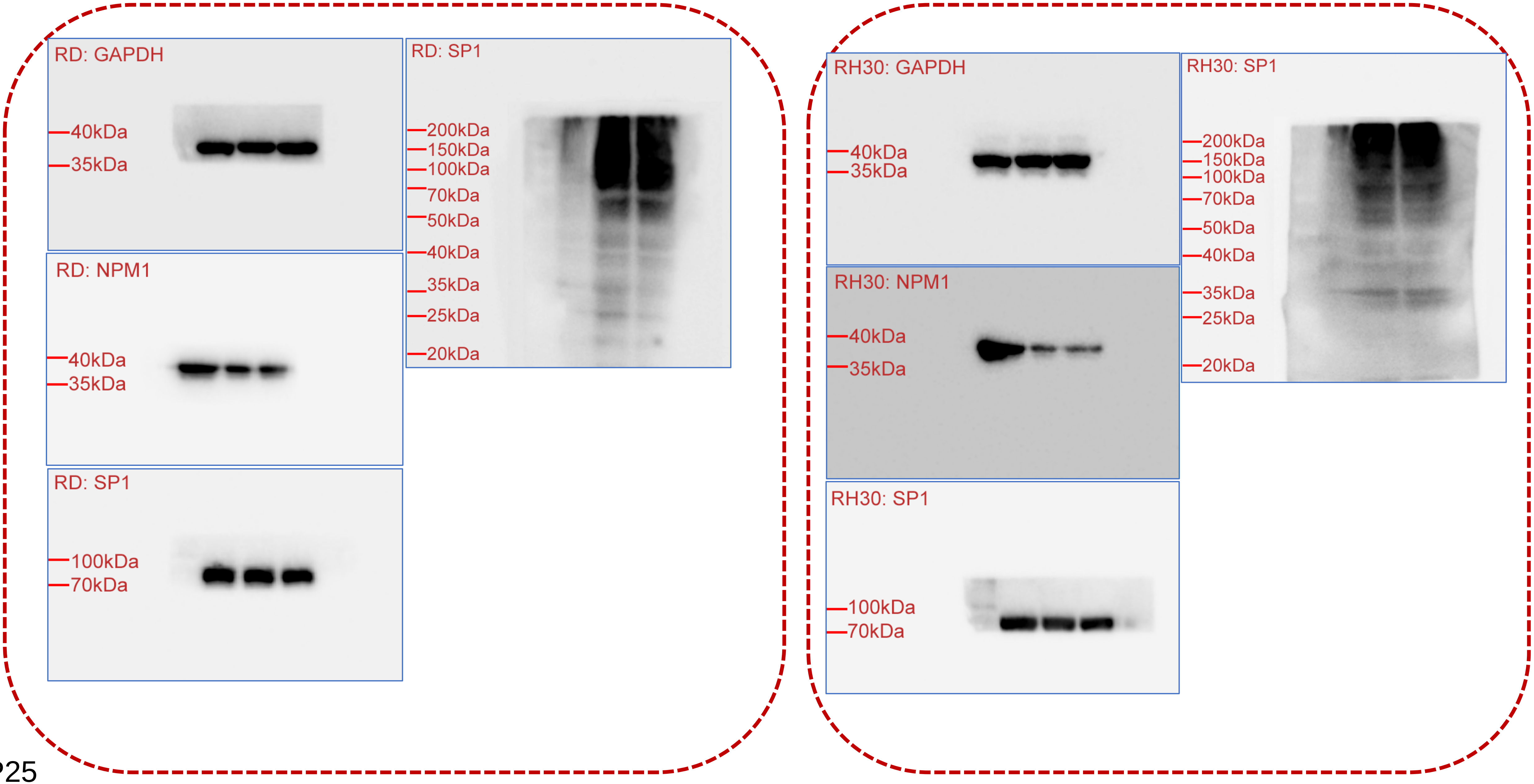

Full unedited blots for Supplementary Figure 7

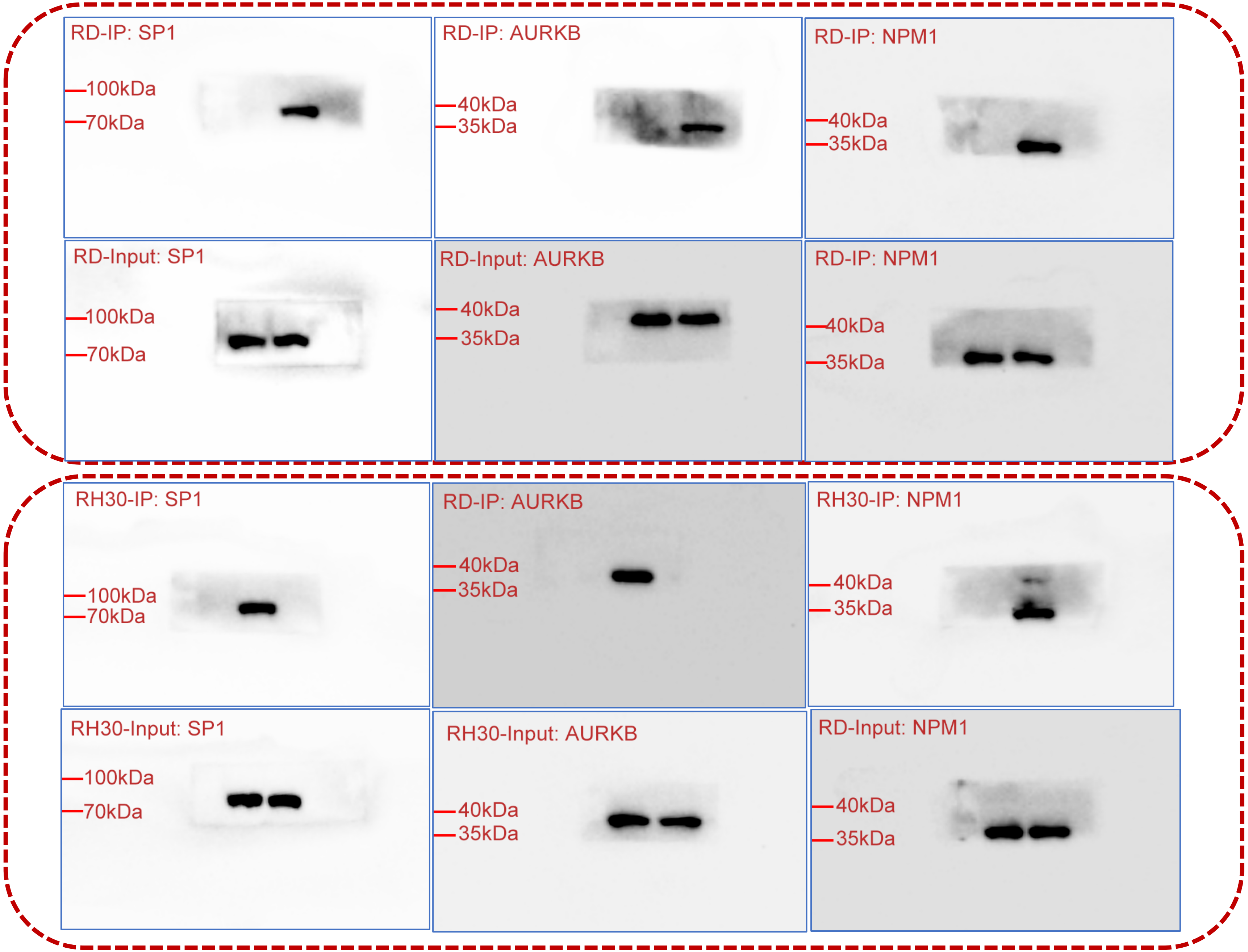

Full unedited blots for Supplementary Figure 10A

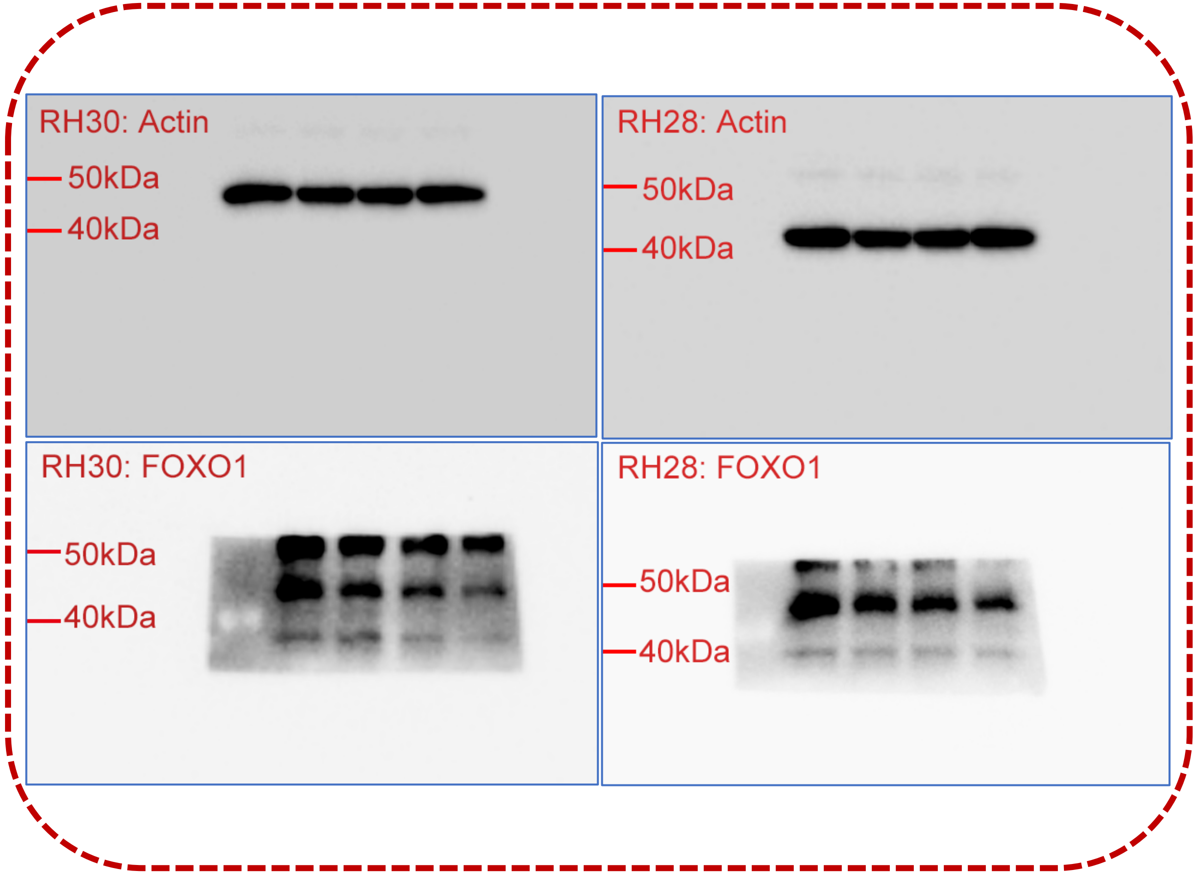

Full unedited blots for Supplementary Figure 10B

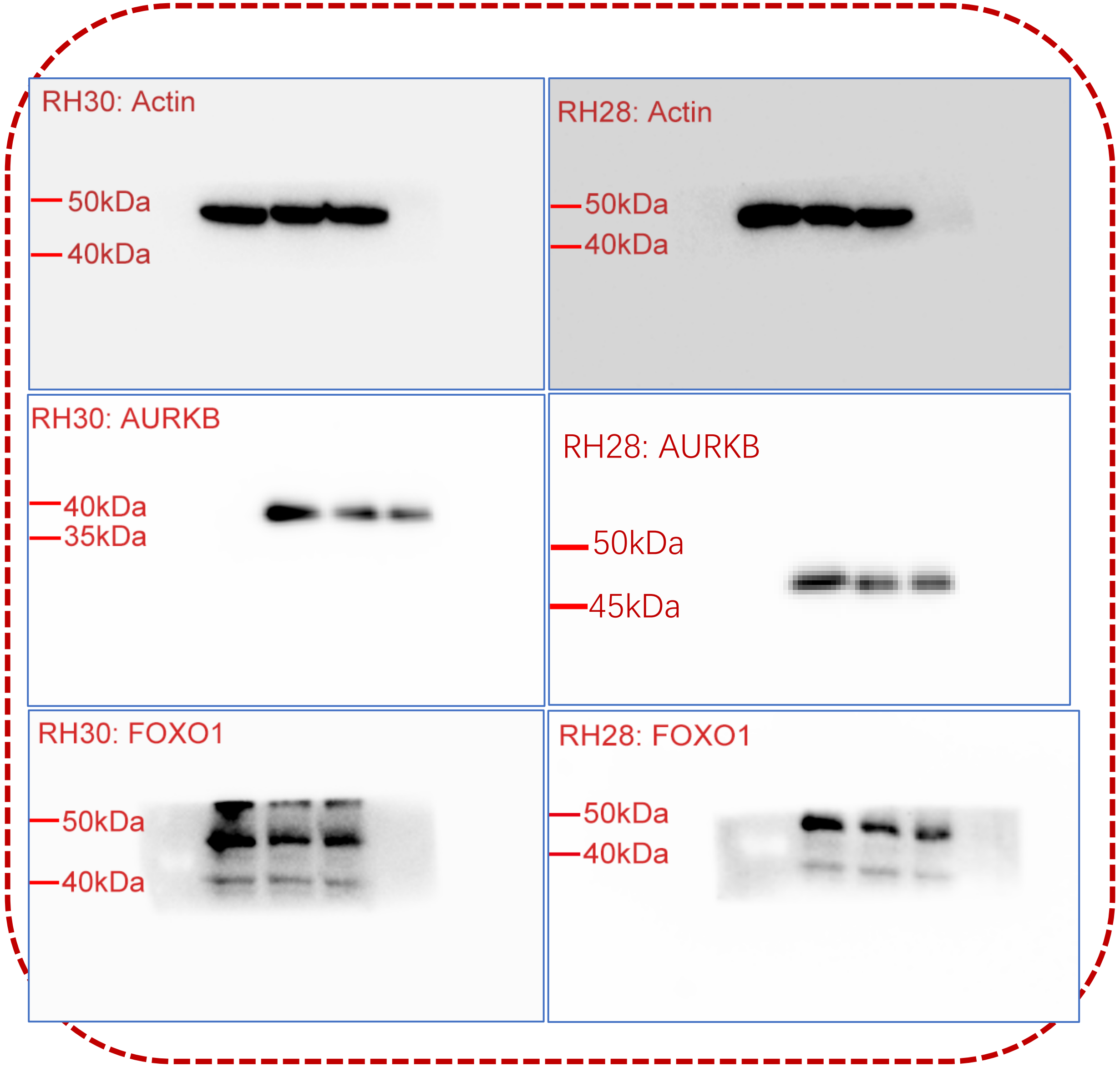

Supplement: Unedited blot and gel images [file jciinsight-10-182429-s222.pdf]
